# Deep immune profiling of patients treated with lenalidomide and dexamethasone with or without daratumumab

**DOI:** 10.1038/s41375-020-0855-4

**Published:** 2020-05-26

**Authors:** Tineke Casneuf, Homer C. Adams, Niels W.C.J. van de Donk, Yann Abraham, Jaime Bald, Greet Vanhoof, Koen Van der Borght, Tina Smets, Brad Foulk, Karl C. Nielsen, Joshua Rusbuldt, Amy Axel, Andrew Lysaght, Hugo Ceulemans, Frederik Stevenaert, Saad Z. Usmani, Torben Plesner, Herve Avet-Loiseau, Inger Nijhof, Tuna Mutis, Jordan M. Schecter, Christopher Chiu, Nizar J. Bahlis

**Affiliations:** 1 Janssen Research & Development, Beerse, Belgium; 2 Janssen Research & Development, LLC, Spring House, PA USA; 3 Department of Hematology, Amsterdam University Medical Center, Vrije Universiteit Amsterdam, Amsterdam, The Netherlands; 4 Immuneering Corp, Cambridge, MA USA; 5 Levine Cancer Institute/Carolinas Healthcare System, Charlotte, NC USA; 6 Vejle Hospital and University of Southern Denmark, Vejle, Denmark; 7 Unite de Genomique du Myelome, CHU Rangueil, Toulouse, France; 8 Janssen Research & Development, LLC, Raritan, NJ USA; 9 Arnie Charbonneau Cancer Research Institute, University of Calgary, Calgary, AB Canada

**Keywords:** Immunology, Cancer therapy

## Abstract

CD38-targeted antibody, daratumumab, is approved for the treatment of multiple myeloma (MM). Phase 1/2 studies GEN501/SIRIUS revealed a novel immunomodulatory mechanism of action (MOA) of daratumumab that enhanced the immune response, reducing natural killer (NK) cells without affecting efficacy or safety. We further evaluated daratumumab’s effects on immune cells in whole blood samples of relapsed/refractory MM patients from both treatment arms of the phase 3 POLLUX study (lenalidomide/dexamethasone [Rd] or daratumumab plus Rd [D-Rd]) at baseline (D-Rd, 40; Rd, 45) and after 2 months on treatment (D-Rd, 31; Rd, 33) using cytometry by time-of-flight. We confirmed previous reports of NK cell reduction with D-Rd. Persisting NK cells were phenotypically distinct, with increased expression of HLA-DR, CD69, CD127, and CD27. The proportion of T cells increased preferentially in deep responders to D-Rd, with a higher proportion of CD8^+^ versus CD4^+^ T cells. The expansion of CD8^+^ T cells correlated with clonality, indicating generation of adaptive immune response with D-Rd. D-Rd resulted in a higher proportion of effector memory T cells versus Rd. D-Rd reduced immunosuppressive CD38^+^ regulatory T cells. This study confirms daratumumab’s immunomodulatory MOA in combination with immunomodulatory drugs and provides further insight into immune cell changes and activation status following daratumumab-based therapy.

## Introduction

Daratumumab is a CD38-targeted monoclonal antibody that has demonstrated activity as monotherapy [1, 2] and in combination with standard-of-care regimens in patients with relapsed/refractory multiple myeloma (MM) and newly diagnosed MM [3–6]. The ability of daratumumab to induce deep and durable responses in MM is thought to be in part due to its direct on-tumor mechanism of action (MOA), including complement-dependent cytotoxicity [7], antibody-dependent cellular cytotoxicity (ADCC) [7], antibody-dependent cellular phagocytosis [8], apoptosis [9], and direct enzymatic inhibition [10]. Recently, we revealed an immunomodulatory MOA of daratumumab, including the expansion of cytotoxic T cells, increase in T-cell repertoire clonality, and elimination of CD38^+^ myeloid-derived suppressor cells, regulatory B cells, and a subpopulation of regulatory T cells (T_regs_; CD4^+^CD25^+^CD127^dim^) [11]. We showed a rapid reduction of natural killer (NK) cells in whole blood (WB) and bone marrow of MM patients after the first dose of daratumumab, to a low level that remained over the course of treatment, and a recovery of these cells after treatment discontinuation [12]. Interestingly, this reduction in NK cells was not complete, and peripheral blood mononuclear cells (PBMCs) from the patients were still capable of inducing lysis by ADCC of CD38^+^ tumor cells in vitro, suggesting that persisting NK cells retained cytotoxic functionality [12].

To further investigate the immunomodulatory effects of daratumumab, we recently utilized mass cytometry by time-of-flight (CyTOF) on WB and/or bone marrow samples from 32 patients treated with daratumumab monotherapy in SIRIUS or GEN501 at baseline and after 2 months of treatment [13]. CyTOF utilizes next-generation, high-parameter, single-cell analysis platforms, overcoming the challenges of classical cytometry by allowing for the simultaneous analysis of cellular composition and marker expression across a wide range of immune cell subpopulations [14–16]. Our analysis revealed the downregulation of CD38 expression across a large number of immune cell types, and the selective depletion of the strongly immunosuppressive fraction of T_regs_ that highly express CD38 [11, 13]. We confirmed NK cell reduction, and CyTOF profiling revealed that daratumumab induced distinct phenotypic changes in the persisting NK cells, with increased expression of CD27, HLA-DR, CD69, and CD137, suggesting that these cells may be capable of daratumumab-mediated ADCC. In addition, we demonstrated a shift to CD8^+^ prevalence with higher granzyme B (GrB) positivity in the T-cell population of responders, supporting monotherapy-induced CD8^+^ T-cell activation [13]. Collectively, these findings supported a broad immunomodulatory role for daratumumab that may contribute to the observed depth of response.

To further expand upon these findings, we conducted CyTOF immune cell profiling of daratumumab in a large patient population and in the presence of a randomized controlled standard-of-care comparator arm on patient samples collected in the phase 3 POLLUX study (ClinicalTrials.gov Identifier: NCT02076009). POLLUX evaluated daratumumab plus lenalidomide and dexamethasone (D-Rd) versus the comparator control regimen (lenalidomide and dexamethasone alone [Rd]) in relapsed/refractory MM [3]. Immune responses in these patients were also evaluated by monitoring the T-cell repertoire by T-cell receptor (TCR) sequencing and gene expression profiling of >700 genes from 24 different immune cell types (NanoString® PanCancer Immune Cell Profiling Panel, NanoString Technologies, Seattle, WA, USA) at baseline and in response to treatment.

## Methods

Samples for CyTOF were analyzed as described previously [13]. Additional details on sample preparation, staining, CyTOF, TCR, and NanoString analyses can be found in Supplementary Methods.

### CyTOF sample sources and staining

The study design and patient population included in the POLLUX study have been previously published [3]. Briefly, WB samples were collected from 40 patients receiving D-Rd and 45 patients receiving Rd at baseline, and matched samples after 2 months of treatment were collected from 31 patients receiving D-Rd and 33 patients receiving Rd. Paired baseline and on-treatment samples were available for 31 D-Rd and 33 Rd patients; samples were available at baseline only for 9 D-Rd and 12 Rd patients. Clinical response data of the POLLUX study were from patients who received D-Rd or Rd at the clinical cutoff date of March 6, 2017. Responders were defined as patients with a best clinical response of partial response or better. Of patients profiled with CyTOF, 7.5% were nonresponders and 92.5% were responders in the D-Rd group, and 22.2% were nonresponders and 77.8% were responders in the Rd group. This was reflective of the intent-to-treat population, with 7.1% nonresponders and 92.9% responders in the D-Rd group and 23.6% nonresponders and 76.5% responders in the Rd group. Samples were processed and antibody staining was performed using a panel of metal-conjugated antibodies specific for lineage markers and other markers of interest (Table S1).

This study was conducted according to the Declaration of Helsinki and the International Conference on Harmonization Good Clinical Practice guidelines, study protocols and analysis plans were approved by ethics committees or institutional review boards at each study site, and all patients provided written informed consent.

### Data preprocessing and quality control

Stained samples were analyzed using a CyTOF C5 system. Gating was performed using Cytobank (www.cytobank.org; Cytobank, Inc, Santa Clara, CA, USA), and data were further processed via custom scripts based on the flowCore package. Channel intensities were normalized with calibration beads following data acquisition, and the arcsinh function with a cofactor of five was used to transform the measured intensities for each channel. Quality control of this sample set was performed using clustering via the Earth Mover’s Distance algorithm [7] on the percentage of cells and marker enrichment modeling [17]. These analyses revealed the absence of technical batch effects and the expected clustering of control samples (Fig. S1a, b).

### Identification and differential analysis of immune cell population counts and marker intensities

Sample groups were compared per population fraction between time points (before and during treatment) and between response groups (responders and nonresponders). In total, 201 patient samples were acquired by CyTOF; 43 samples did not meet the quality criteria and were excluded from the analysis, along with nine corresponding cycle (C) 3 day (D) 1 samples of excluded C1D1 samples (i.e., paired samples from the same patients). After quality assessment and selection of samples with >10,000 live singlet events of lymphocyte/monocyte count, 149 patient samples and 38 control samples were clustered into nodes of similar cellular events using the spanning-tree progression analysis of density-normalized events (SPADE) algorithm [16, 18], and subsequently gated into immune populations (Table S2) via Cytobank software [5, 6]. The SPADE algorithm was run using 150 nodes with a downsampling rate of 10% to maximize the number of cell populations while minimizing the number of empty nodes. Cell fraction was calculated for the total number of cells (%total) for each node or bubble of each sample and relative to the “parent” populations in the SPADE tree hierarchy (%parent). Differential analysis of population fractions and marker intensity over time and between treatment groups was conducted by calculating the mean marker intensity (MMI) for each cluster in the SPADE tree and performing a two-sided two-sample *t*-test, allowing for unequal group variance to compare MMI values for each group (time/response). Raw *P* values for all cluster and marker combinations were generated by this differential analysis, and bootstrap-adjusted *P* values were calculated to correct for multiple dependent hypothesis testing. In the event that repeated observations over time occurred for the same subject, response group means were calculated for the fold changes (MMI difference between two time points).

### Bin analysis

To quantify changes in the distribution of signal intensity of a given marker, centiles of the single-cell data across all conditions were calculated for each given population and channel of interest. Those values were then used to define bins; bins with overlapping values were combined in the event that >1% of cells had similar signal intensity. The fraction of cells in each bin for each condition of interest was compared with the total number of cells within the entire bin to enable comparisons across conditions. The effect of treatment over time was visualized by plotting the cell fraction and the lower limit of intensity of each bin.

To estimate the significance of differences observed using centile bins, the empirical cumulative distribution function of the signal intensity for each condition was calculated. To control for differences in number of cells per sample, each point was weighted according to the total number of cells in the corresponding sample. The test statistic corresponds to the difference between empirical cumulative distribution functions. To estimate significance, a null distribution was constructed in which conditions are assumed to be identical by computing the empirical cumulative distribution functions difference after condition labels were randomly permuted. The *P* value was computed by comparing the true empirical cumulative distribution functions difference with the null distribution.

### Visualization

MMI differential testing results were visualized in a SPADE-blend tree by coloring each SPADE tree cluster using a combination of raw *P* values and fold changes computed after changes in marker intensities or population fractions. Numbers (nodes) grayed out in SPADE trees were not included in the analysis due to a restricted parent–child population comparison or the existence of an empty node for one patient sample in the respective data set. Radviz projections [19] allow for the comparison of populations and conditions while preserving the relation to original dimensions. We used this new method to visualize single-cell level trends. Treatment effects on specific subsets of cells were visualized using appropriate channels representing different phenotypic and transitional markers, and Radviz shifts were used to direct manual gating and downstream statistical analysis. Fan charts developed by the Bank of England [20] were used to examine the individual contributions of each channel and assess the homogeneity of the response across a given cell population. In brief, the centiles for each marker and each condition were calculated, and the corresponding values were visualized as stacked area plots color-coordinated to their corresponding centiles. The color intensity is greatest at the center of each fan chart (centered on the 50th centile) and decreases symmetrically across the spectrum of higher and lower centiles.

### NanoString analysis

Paired PBMC samples (collected on D1 of C1 and C3) were prepared for profiling on the nCounter PanCancer Immune Profiling for Human cells (http://www.nanostring.com/products/gene_expression_panels) to probe a panel of >700 genes involved in immune processes such as activation response, evasion of immune recognition, and suppression of immune activity (Fig. S2).

Filtration and normalization were performed on all samples that passed quality control. Sample pairing was accounted for using a random-effect term and correlation estimation using a limma::duplicateCorrelation function. Here, an expression matrix with 292 samples × 490 genes was used in a limma-based differential expression analysis pipeline. Sample numbers per analysis group were: D-Rd C1D1, 75; D-Rd C3D1, 77 (71 paired); Rd C1D1, 70; Rd C3D1, 70 (65 paired).

NanoString data analyses were also conducted on patient samples for which both NanoString and CyTOF data were available, using CyTOF-derived cellular abundance estimates as covariates in the model matrix, in addition to standard response-based differential expression tests to remove the contribution of bulk cell type differences from the differential expression signal and allow for the focus on altered transcriptional behavior. This NanoString-CyTOF matched profiled data set consisted of: D-Rd C1D1, 22; D-Rd C3D1, 18 (16 paired); Rd C1D1, 23; Rd C3D1, 15 (15 paired).

### T-cell clonality and richness analyses

TCRβ sequencing was conducted on PBMCs from paired WB samples using the ImmunoSEQ™ assay (Adaptive Biotechnologies, Seattle, WA, USA). The main metrics evaluated at baseline and upon treatment include T-cell clonality, defined as the extent of mono- or oligoclonal expansion, and T-cell richness, defined as the number of clones with unique TCRβ rearrangements.

### Data sharing statement

The data sharing policy of Janssen Pharmaceutical Companies of Johnson & Johnson is available at https://www.janssen.com/clinical-trials/transparency. As noted on this site, requests for access to the study data can be submitted through Yale Open Data Access Project site at http://yoda.yale.edu.

## Results

### Extensive and broad downregulation of CD38 expression and changes in immune cell composition by daratumumab

**Fig. 1:**
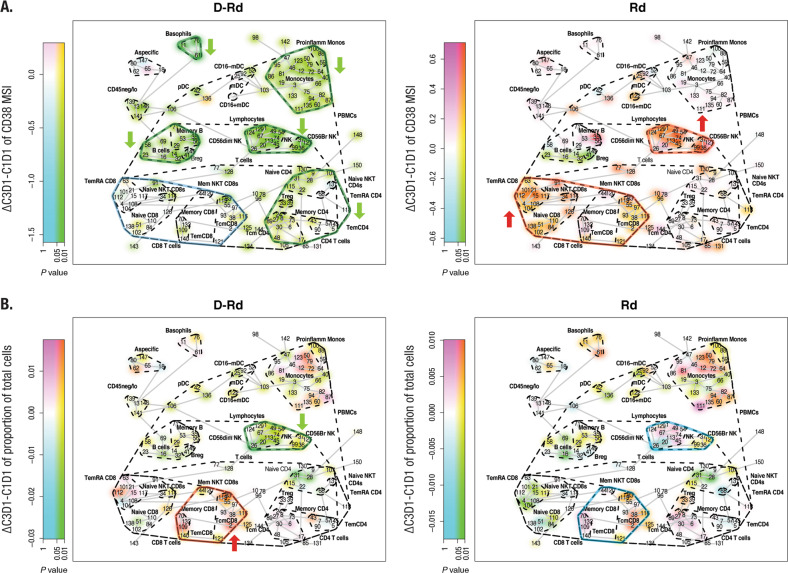
D-Rd and Rd mediated changes of mean CD38 expression on immune cell populations and mean total immune cell populations after 2 months of therapy. Panel **a** shows the mean CD38 expression on immune cell populations and panel **b** shows the mean total immune cell populations after 2 months of therapy for D-Rd treatment (left) and Rd treatment (right). The visualization method used is the SPADE‐blend tree with which the differential testing results (here, contrasting pre‐ versus on‐treatment sample data) are projected as colored highlighting on the SPADE tree to maximize exploration of the statistical analysis results, simultaneously, in the samples under investigation. Br bright, B_reg_ regulatory B cell, D-Rd daratumumab plus lenalidomide and dexamethasone, mDC myeloid dendritic cell, Monos monocytes, NK natural killer, NKT natural killer T cell, PBMC peripheral blood mononuclear cell, pDC plasmacytoid dendritic cell, Rd lenalidomide and dexamethasone, T_em_ effector memory T cell, T_EM_RA effector memory CD45RA^+^ T cell, T_reg_ regulatory T cell. Nodes are colored by decrease (cyan; green, if significant [raw *P* value]) or increase (magenta; red, if significant [raw *P* value]).

Across the majority of immune cell types investigated (Table S2), CD38 expression was downregulated with D-Rd (Fig. 1a, left). In particular, significant downregulation was observed for NK cells, B cells, basophils, monocytes, and CD4^+^ T cells and no substantial change for CD8^+^ T cells. In contrast, Rd alone caused an upregulation of CD38 in NK cells and CD8^+^ T cells with a significant upregulation in naive CD8^+^ T cells. Except for the nonmemory B cells, Rd did not downregulate CD38 expression in any of the cell subsets (Fig. 1a, right). Furthermore, D-Rd treatment showed a significant reduction in the CD56^dim^ NK cells and, to a lesser extent, a reduction in the CD56^bright^ NK cells, compared with no substantial changes in the NK cells or their subpopulations in the control arm. A significant expansion of various memory CD8^+^ T-cell subtypes was observed with D-Rd after 2 months of therapy (Fig. 1b, left), whereas the expansion in memory CD8^+^ T cells with Rd was not significant (Fig. 1b, right). Immune cell gene expression profiling with NanoString strengthened this finding (Fig. S3a; Table S3a, b). Significantly, decreased expression levels were observed with D-Rd for genes such as SH2D1B (EAT2) and NK-expressed lectin-like receptors KLRF1, KLRC1, and KLRC2 [21]. In addition, expression levels of killer cell immunoglobulin-like activating receptor (KIR) genes, which suppress the killing function of NK cells through recognition of major histocompatibility complex molecules, were also decreased with D-Rd (Fig. S3a, left; Table S3a). Increased RNA expression for genes including CD8A and CD8B, which play a role in T-cell activation [22], and LAG3, a checkpoint molecule expressed on the surface of activated lymphocytes [23], was observed with D-Rd treatment. Gene expression changes in the immune cell compartment were observed with Rd, but these were moderate and not confined to a specific cell type (Fig. S3a, right, c; Table S3b).

### Phenotype of persistent NK cells

**Fig. 2:**
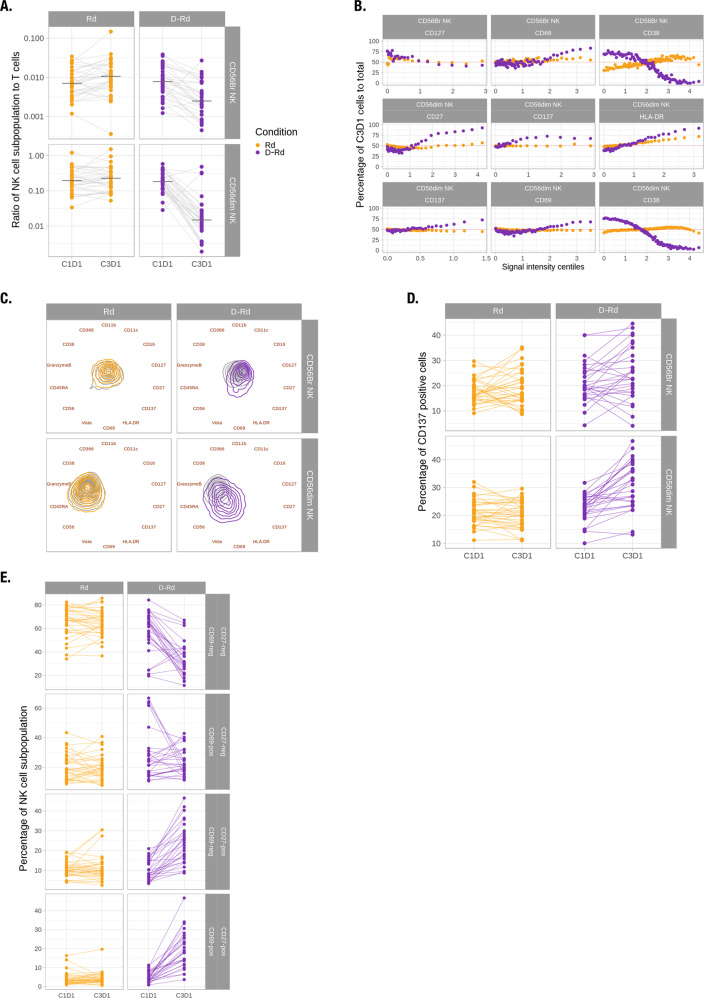
Persistent NK cells were shifted toward an activated, less mature phenotype upon D-Rd treatment. **a** Ratio of CD56^bright^ and CD56^dim^ cells relative to the total number of NK cells at baseline and upon treatment, normalized to the total number of T cells. **b** On-treatment to baseline ratio of positive cell fractions in D-Rd and Rd in function of the signal intensity centiles for a subset of markers across CD56^bright^ and CD56^dim^ NK cells. **c** Radviz projection with contour plots representing marker expression levels of CD56^bright^ and CD56^dim^ cells at baseline and upon D-Rd and Rd treatment. **d** Percentages of CD137^+^CD56^dim^ and CD56^bright^ NK cells relative to total CD56^dim^ and CD56^bright^ NK cells, respectively. **e** Percentages of subtypes of CD27 and CD69 NK cells relative to total NK cells. Br bright, D-Rd daratumumab plus lenalidomide and dexamethasone, NK natural killer, Rd lenalidomide and dexamethasone.

Consistent with previous findings in daratumumab monotherapy studies GEN501 and SIRIUS [13], the number of NK cells was significantly reduced in patients treated with D-Rd compared with Rd. Interestingly, this decrease induced by D-Rd was more pronounced in CD56^dim^ than in CD56^bright^ NK cells (Fig. 2a). The ratio of CD56^dim^ to CD56^bright^ is significantly decreased upon treatment in patients treated with D-Rd (*P* < 0.01; Table S4a). Three patients treated with D-Rd had NK cell levels that remained stable or increased and also had NK cells that were phenotypically distinct from those of the rest of the patient population (Fig. S4a). Because the treatment-induced changes observed were dominated by these three patients, we focused our in-depth analyses of NK cell subtypes, excluding corresponding samples (Fig. 2b-e), though the same analyses were conducted on all patients (Fig. S4b–e). We first evaluated changes in the expression of NK cell markers using BinAnalysis. While changes in median CD38 expression suggest a strong and significant decrease (Fig. 1a), when considering the shape of the distribution D-Rd induces a shift toward cells expressing higher levels of CD38 in both CD56^dim^ and CD56^bright^ NK cells (Fig. 2b). D-Rd also induced increased expression of CD27, HLA-DR, CD69, and CD137 in persisting CD56^dim^ NK cells that were of a larger magnitude than with Rd (*P* < 0.05; absolute effect ≥ 0.005 for each marker). Persistent CD56^bright^ NK cells had increased CD69 and decreased CD127 expression upon D-Rd treatment, with magnitudes greater than observed with Rd (*P* < 0.05; absolute effect ≥ 0.04 for both markers, Fig. 2b). Visualizing the overall difference between treated and untreated samples over these markers, we observed that Rd has virtually no effect on NK cell phenotype, while D-Rd induced a shift in both subpopulations, with CD56^dim^ NK cells being more affected than CD56^bright^ cells (Fig. 2c). CD27 and CD127 have been suggested as maturation markers for CD56^dim^ and CD56^bright^ NK cell subsets, respectively [24–26]. CD69 and HLA-DR are known markers of the activation of CD56^dim^ NK cells, while CD137 expression has been associated with the activation of ADCC in CD56^dim^ NK cells [27–29]. We therefore investigated CD69^+^, CD27^+^, CD127^+^, and CD137^+^ NK cell subpopulations in greater detail. The percentage of CD137^+^CD56^dim^ NK cells was significantly higher with D-Rd (mean signal intesity [MSI] > 0.25 as CD137^+^; *P* < 0.01; Table S4b), but not with Rd (Fig. 2d). Similar increases in the percentage of CD27^+^, CD69^+^, and HLA-DR^+^ populations were observed with D-Rd, while the percentage of CD127^+^CD56^bright^ NK cells was decreased (data not shown). To investigate the relationship between activation and the maturation status of NK cells, we compared populations expressing CD27 and CD69. While the proportion of cells remained constant with Rd treatment, the percentage of CD27^−^CD69^−^ cells was significantly decreased and CD27^+^CD69^−^ and CD27^+^CD69^+^ populations significantly increased (MSI > 1 as CD69^+^ and CD27^+^; *P* < 0.01; Fig. 2e; Table S4c). Cells corresponding to CD27^−^CD69^+^ were not significantly modulated upon D-Rd treatment. Altogether, these findings suggest that NK cells are reduced, while undergoing a selection process in response to D-Rd, resulting in a relative increase in cells of an immature activated phenotype.

Upon correction of the NanoString immune cell gene expression data for the cell abundances measured with CyTOF, *P* values for NK cell- and CD8^+^ T-cell–related gene expression were no longer significant for D-Rd (Fig. S3b, left; Table S3c). This implies that the decreased expression of NK cell genes (SH2D1B, KLRF1, KLRC1, KLRC2, and NCAM1; Table S3a) observed with uncorrected NanoString data is best explained by a decrease in the numbers of NK cells and not transcriptional downregulation. Similarly, the increase in the expression of CD8A, CD8B, and LAG3 is better explained by CD8^+^ T-cell expansion (see “T-cell phenotyping” below), not increased transcription (Table S3a versus c).

Upon correction of cell abundance changes measured with CyTOF for Rd (Fig. S3b, right; Table S3d), NK and T-cell genes did not differ significantly in expression levels, with the exception of KIR activating genes with short cytoplasmic domain that transduce activating signals (KIR_activating_subgroup_2; KIR2DS1, KIR2DS2, KIR2DS3, KIR2DS4, and KIR2DS5).

### T-cell phenotyping

**Fig. 3:**
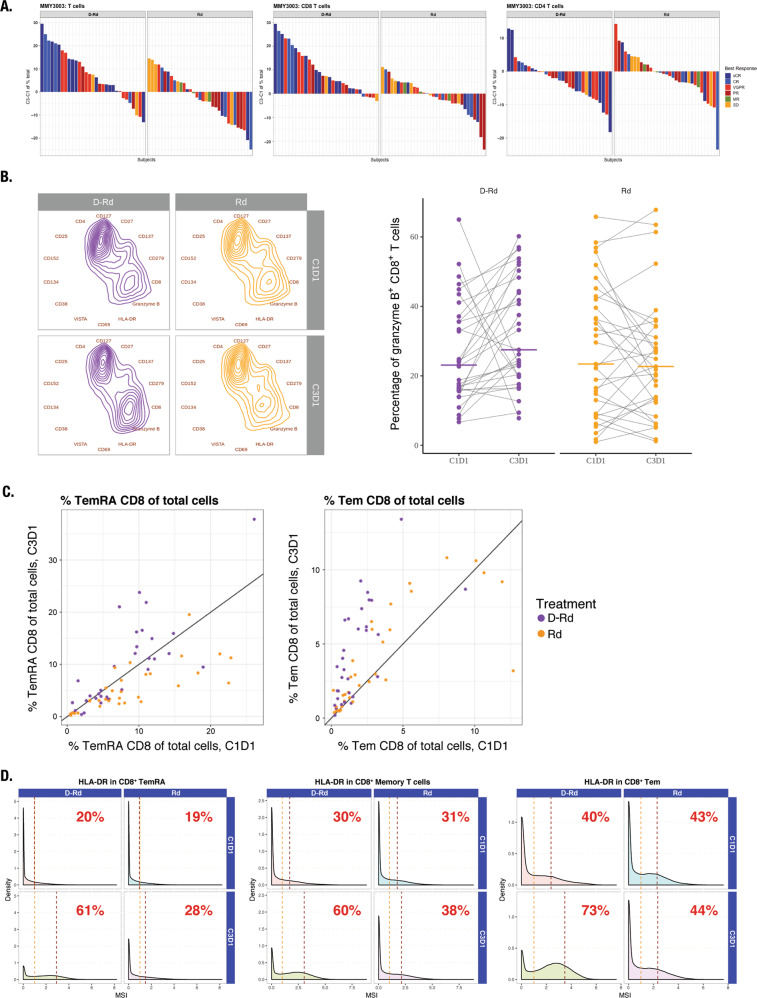
D-Rd increased the proportion of T cells preferentially in deep responders, shifted CD3^+^ T cells toward a CD8^+^GrB^+^ phenotype, increased the proportion of effector memory T cells, and increased HLA-DR expression on T-cell subtypes compared with Rd. Panel **a** shows increases in the proportion of T cells preferentially in deep responders in response to D-Rd treatment; panel **b** shows the shift of CD3^+^ T cells toward a CD8^+^GrB^+^ phenotype in response to D-Rd treatment; panel **c** shows an increase in the proportion of effector memory T cells in response to D-Rd treatment*; and panel **d** shows increased HLA-DR expression on T-cell subtypes in response to D-Rd treatment, compared with Rd. C cycle, CR complete response, D day, D-Rd daratumumab plus lenalidomide and dexamethasone, MR minimal response, MSI mean signal intensity, PR partial response, Rd lenalidomide and dexamethasone, sCR stringent complete response, SD stable disease, T_em_ effector memory T cells, T_EM_RA effector memory CD45RA^+^ T cells, VGPR very good partial response. *Line represents 1:1 diagonal.

Significant increases were observed in the proportion of total T cells and various T-cell subsets with D-Rd, but not Rd (Fig. 3; Table S5). Interestingly, the proportion of T cells increased preferentially in deep responders (complete response or better) receiving D-Rd and correlated with a higher proportion of CD8^+^ versus CD4^+^ T cells (Fig. 3a): CD3^+^ T cells evaluated for several markers of activation and exhaustion revealed a shift in composition toward CD8^+^GrB^+^ T cells in response to D-Rd (CD8^+^ T cells, *P* < 0.01; CD8^+^GrB^+^ T cells, *P* < 0.05; Fig. 3b). We corroborated this observation by paired-sample analysis of the percentage of CD8^+^ cells in which this increase was distinct for D-Rd–treated patients (*P* < 0.01; Fig. 3b). D-Rd led to a higher proportion of effector memory (T_EM_) and effector memory CD45RA^+^ (T_EM_RA) cells versus Rd (Fig. 3c; Table S5). Greater increases in HLA-DR expression were observed in D-Rd–treated versus Rd-treated patients, particularly for memory and CD8^+^ T_EM_ (Fig. 3d).

**Fig. 4:**
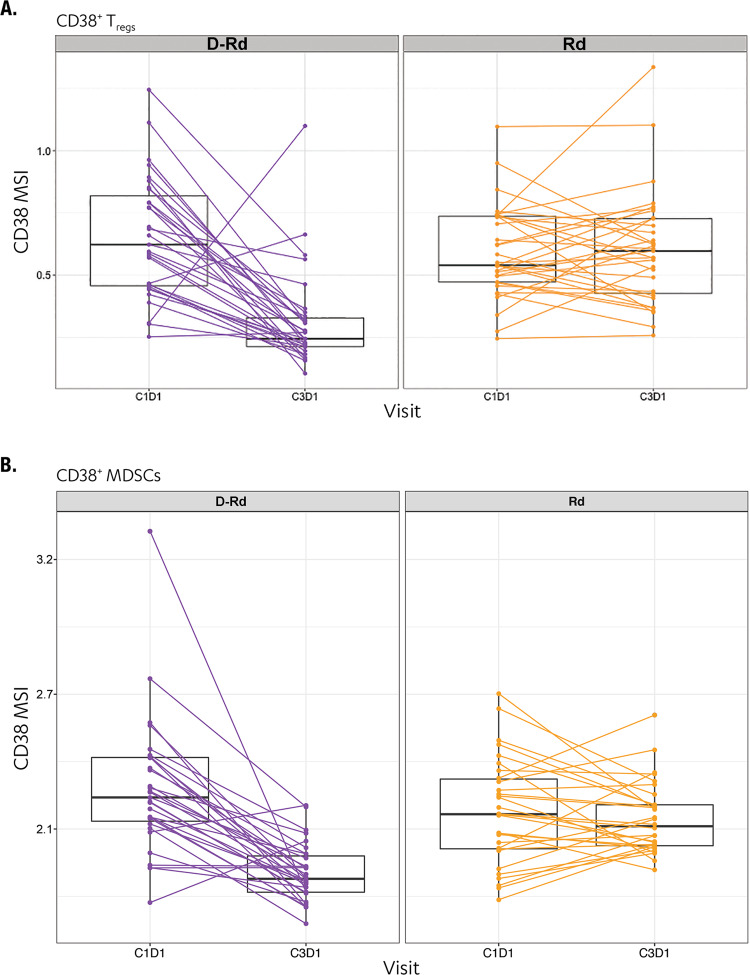
D-Rd treatment reduces immunosuppressive CD38^+^ T_regs_ and MDSCs compared with Rd. Panel **a** shows T_reg_ population; panel **b** shows MDSC population. C cycle, D day, D-Rd daratumumab plus lenalidomide and dexamethasone, MDSC myeloid-derived suppressor cell, MSI mean signal intensity, Rd lenalidomide and dexamethasone, T_reg_ regulatory T cell.

Consistent with previous reports of daratumumab monotherapy studies [13], we observed the depletion of immunosuppressive CD38^+^ T_regs_ (CD4^+^CD25^+^CD127^−^) in patients who received D-Rd for 2 months (Figs. 4a and S5; Table S6). Interestingly, this depletion was not observed in patients treated with Rd alone (Figs. 4a and S5). WB samples were analyzed to assess rare CD38^+^ myeloid-derived suppressor cell populations, and we detected a selective reduction of these monocytic myeloid-derived suppressor cells with D-Rd versus Rd (Fig. 4b; Table S6).

### D-Rd promotes adaptive T-cell responses

**Fig. 5:**
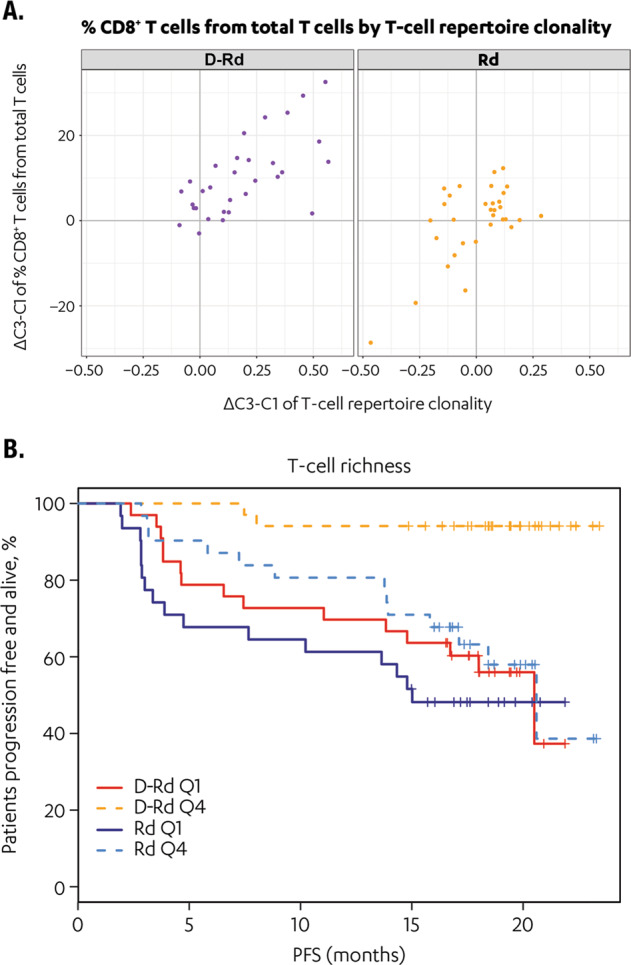
T-cell repertoire clonality increases in patients receiving D-Rd versus Rd and baseline TCR richness is predictive of improved PFS in patients treated with D-Rd. Panel **a** compares TCR clonality in patients receiving D-Rd versus Rd treatment; panel **b** shows the correlation of baseline TCR richness and improved PFS in patients treated with D-Rd versus Rd. D-Rd daratumumab plus lenalidomide and dexamethasone, PFS progression-free survival, Q quartile, Rd lenalidomide and dexamethasone, TCR T-cell receptor.

TCR sequencing revealed a significant increase in median clonality of the T-cell repertoire with D-Rd (median at baseline, 0.166 to median following treatment, 0.263; *P* = 3.0e^−12^), but not with Rd (*P* = 0.66, baseline levels not different between treatment arms; Fig. S6a, b). Importantly, this clonality increase of the T-cell repertoire was correlated significantly with the expansion of CD8^+^ T cells observed by CyTOF, suggesting daratumumab induced adaptive T-cell responses (Fig. 5a; Table S7). This effect was not observed with Rd alone, supporting the hypothesis that clonal expansion of T cells can be specifically attributed to daratumumab. Moreover, high baseline TCR richness is determined to be predictive of improved progression-free survival in patients treated with D-Rd (*P* = 0.034) but not Rd (*P* = 0.239; Fig. 5b). T-cell clonality (D-Rd: *P* = 0.12; Rd: *P* = 0.76) was not predictive of progression-free survival for either treatment arm (data not shown). In addition to observed increased clonality in D-Rd–treated patients, T-cell richness decreased significantly with D-Rd (*P* = 9.2e^−5^) but not with Rd (*P* = 0.52; Fig. S6c).

## Discussion

The current study presents the results of comprehensive omics profiling conducted to further elucidate and support the mode of action of the CD38-targeted monoclonal antibody daratumumab. We recently reported results in a small patient population treated with monotherapy, which demonstrated the immunomodulatory effects of daratumumab: (1) expansion of cytotoxic T cells; (2) reduction of immune suppressive cells, including CD38^+^ myeloid-derived suppressor cells, regulatory B cells, and a subpopulation of regulatory T_regs_ (CD4^+^CD25^+^CD127^dim^), to promote T-cell activity against myeloma cells; and (3) increase in T-cell repertoire clonality, reduction of NK cells, and downregulation of CD38 on target cells [11, 30]. To explore these effects in a larger patient population, in greater depth, and in respect to a comparator treatment, we conducted comprehensive omics profiling in the phase 3 POLLUX study evaluating D-Rd versus Rd alone in relapsed/refractory MM. Broad and deep profiling was conducted through combined TCR sequencing, mass cytometry, and immune cell transcriptomic profiling to investigate the effect of daratumumab combination therapy on the T-cell repertoire, immune cell composition, and gene expression status. Our objective was to correlate immune cell phenotypic changes with efficacy and depth of response observed in patients receiving D-Rd versus Rd and to explore whether D-Rd promotes adaptive T-cell responses.

Our data show that D-Rd specifically downregulates the target CD38 on various immune cell types. Notably, transfer of daratumumab-CD38 complexes from tumor or normal cells to immune effector cells (trogocytosis) results in reduced levels of CD38 on the cell surface [31]. Furthermore, several immune cell composition shifts were induced by D-Rd versus Rd alone, including increases in total T cells, as well as subsets of effector and memory CD8^+^ T cells, with a preferential increase in CD8^+^ T cells in deep responders. Importantly, these effector and CD8^+^ T_EM_ cells show an activated phenotype, with the upregulation of activation markers such as GrB and HLA-DR. Consistent with previous reports [12, 13], we observed a rapid reduction in NK cells in response to treatment with D-Rd, and levels remained low during the course of treatment and recovered after treatment ended. Utilization of high-resolution CyTOF revealed a shift of CD56^dim^ and CD56^bright^ NK subpopulations after treatment, with several markers showing differences in expression levels between D-Rd and Rd alone. D-Rd increased the proportion of CD27^+^, CD69^+^, and CD137^+^CD56^dim^ NK cells, suggesting that the persisting NK cells are immature and activated with increased cytotoxic potential [32]. We showed a significant increase in the proportion of immature NK cells with increased expression of maturation marker CD27 on CD56^dim^ NK cells. Importantly, PBMCs from daratumumab-treated patients still induced lysis by ADCC of CD38^+^ tumor cells in vitro [33, 34]. These results, together with observations from this current study, suggest consumption and maturation of CD56^bright^ NK cells with retained cytotoxic functionality of the persisting NK cells, and an influx of less mature NK cells [35]. No changes were observed in either the CD56^dim^ or CD56^bright^ NK cell proportions in the Rd treatment arm. However, CD38 expression was substantially upregulated in NK cells and some T-cell subsets.

TCR sequencing revealed robust increases in T-cell clonality and decreases in T-cell richness by D-Rd treatment that were not observed with Rd. Of particular interest is the finding that high TCR richness at baseline was predictive of improved progression-free survival with D-Rd, analogous to the reported findings with immune checkpoint inhibitors [36]. Interestingly, TCR repertoire richness has been previously associated with favorable outcomes in patients with breast cancer [37]. Preserved immunocompetence may be an important prerequisite to obtain the optimal effect of boosting the immune response with daratumumab and suggests that the use of daratumumab in early lines of therapy before the immune system has been exhausted by other treatments may result in the greatest benefit.

Together with the immunophenotyping data, our TCR sequencing results demonstrated that T-cell clonality increases are correlated with the expansion of CD8^+^ T cells, suggesting an adaptive immune response in D-Rd–treated patients. Such increases in T-cell clonality have been previously observed in settings that include vaccination studies, clearance of viral infection, and the expansion of tumor-specific T cells [38].

In conclusion, the immune modifications induced by the addition of daratumumab to Rd in this study are largely consistent with the observations seen in daratumumab monotherapy studies and confirm that daratumumab’s immunomodulatory MOA remains operational when daratumumab is used in combination with lenalidomide. This study provides additional insights into changes in relative numbers and activation status of NK cells and T-cell subtypes following daratumumab-based therapy.

## Supplementary information

Supplemental Material

## References

[1] Lonial S, Weiss BM, Usmani S, Singhal S, Chari A, Bahlis N (2016). Daratumumab monotherapy in patients with treatment-refractory multiple myeloma (SIRIUS): an open-label, randomised, phase 2 trial. Lancet..

[2] Lokhorst HM, Plesner T, Laubach JP, Nahi H, Gimsing P, Hansson M (2015). Targeting CD38 with daratumumab monotherapy in multiple myeloma. N Engl J Med.

[3] Dimopoulos MA, Oriol A, Nahi H, San-Miguel J, Bahlis N, Usmani S (2016). Daratumumab, lenalidomide, and dexamethasone for multiple myeloma. N Engl J Med.

[4] Palumbo A, Chanan-Khan A, Weisel K, Nooka AK, Masszi T, Beksac M (2016). Daratumumab, bortezomib, and dexamethasone for multiple myeloma. N Engl J Med.

[5] Chari A, Suvannasankha A, Fay JW, Arnulf B, Kaufman JL, Ifthikharuddin JJ (2017). Daratumumab plus pomalidomide and dexamethasone in relapsed and/or refractory multiple myeloma. Blood..

[6] Mateos MV, Dimopoulos MA, Cavo M, Suzuki K, Jakubowiak A, Knop S (2018). Daratumumab plus bortezomib, melphalan, and prednisone for untreated myeloma. N Engl J Med.

[7] de Weers M, Tai YT, van der Veer MS, Bakker JM, Vink T, Jacobs DC (2011). Daratumumab, a novel therapeutic human CD38 monoclonal antibody, induces killing of multiple myeloma and other hematological tumors. J Immunol..

[8] Overdijk MB, Verploegen S, Bogels M, van Egmond M, Lammerts van Bueren JJ, Mutis T (2015). Antibody-mediated phagocytosis contributes to the anti-tumor activity of the therapeutic antibody daratumumab in lymphoma and multiple myeloma. MAbs..

[9] Overdijk MB, Jansen JH, Nederend M, Lammerts van Bueren JJ, Groen RW, Parren PW (2016). The therapeutic CD38 monoclonal antibody daratumumab induces programmed cell death via Fc gamma receptor-mediated cross-linking. J Immunol.

[10] Lammerts van Bueren J, Jakobs D, Kaldenhoven N, Roza M, Hiddingh S, Meesters J (2014). Direct in vitro comparison of daratumumab with surrogate analogs of CD38 antibodies MOR03087, SAR650984 and Ab79. Blood..

[11] Krejcik J, Casneuf T, Nijhof IS, Verbist B, Bald J, Plesner T (2016). Daratumumab depletes CD38^+^ immune-regulatory cells, promotes T-cell expansion, and skews T-cell repertoire in multiple myeloma. Blood..

[12] Casneuf T, Xu XS, Adams HC, Axel AE, Chiu C, Khan I (2017). Effects of daratumumab on natural killer cells and impact on clinical outcomes in relapsed refractory multiple myeloma. Blood Adv.

[13] Adams HC, Stevenaert F, Krejcik J, Van der Borght K, Smets T, Bald J (2019). High-parameter mass cytometry evaluation of relapsed/refractory multiple myeloma patients treated with daratumumab demonstrates immune modulation as a novel mechanism of action. Cytometry A..

[14] Bandura DR, Baranov VI, Ornatsky OI, Antonov A, Kinach R, Lou X (2009). Mass cytometry: technique for real time single cell multitarget immunoassay based on inductively coupled plasma time-of-flight mass spectrometry. Anal Chem.

[15] Autissier P, Soulas C, Burdo TH, Williams KC (2010). Evaluation of a 12-color flow cytometry panel to study lymphocyte, monocyte, and dendritic cell subsets in humans. Cytometry A.

[16] Bendall SC, Simonds EF, Qiu P, Ae AD, Krutzik PO, Finck R (2011). Single-cell mass cytometry of differential immune and drug responses across a human hematopoietic continuum. Science.

[17] Diggins KE, Greenplate AR, Leelatian N, Wogsland CE, Irish JM (2017). Characterizing cell subsets using marker enrichment modeling. Nat Methods..

[18] Qiu P, Simonds EF, Bendall SC, Gibbs KD, Bruggner RV, Linderman MD (2011). Extracting a cellular hierarchy from high-dimensional cytometry data with SPADE. Nat Biotechnol.

[19] Abraham Y, Gerrits B, Ludwig MG, Rebhan M, Gubser Keller C (2017). Exploring glucocorticoid receptor agonists mechanism of action through mass cytometry and radial visualizations. Cytom B Clin Cytom.

[20] Britton E, Fisher P, Whitley J. The inflation report projections: understanding the fan chart. The Bank of England quarterly bulletin Q1. 1998:30–7.

[21] Middleton D, Curran M, Maxwell L (2002). Natural killer cells and their receptors. Transpl Immunol..

[22] Konno A, Okada K, Mizuno K, Nishida M, Nagaoki S, Toma T (2002). CD8alpha alpha memory effector T cells descend directly from clonally expanded CD8alpha +beta high TCRalpha beta T cells in vivo. Blood..

[23] Goldberg MV, Drake CG (2011). LAG-3 in cancer immunotherapy. Curr Top Microbiol Immunol.

[24] Vukicevic M, Chalandon Y, Helg C, Matthes T, Dantin C, Huard B (2010). CD56bright NK cells after hematopoietic stem cell transplantation are activated mature NK cells that expand in patients with low numbers of T cells. Eur J Immunol.

[25] Gasteiger G, Hemmers S, Bos PD, Sun JC, Rudensky AY (2013). IL-2-dependent adaptive control of NK cell homeostasis. J Exp Med.

[26] Melsen JE, Lugthart G, Lankester AC, Schilham MW (2016). Human circulating and tissue-resident CD56(bright) natural killer cell populations. Front Immunol..

[27] Masu T, Atsukawa M, Nakatsuka K, Shimizu M, Miura D, Arai T (2018). Anti-CD137 monoclonal antibody enhances trastuzumab-induced, natural killer cell-mediated cytotoxicity against pancreatic cancer cell lines with low human epidermal growth factor-like receptor 2 expression. PLoS One.

[28] Lin W, Voskens CJ, Zhang X, Schindler DG, Wood A, Burch E (2008). Fc-dependent expression of CD137 on human NK cells: insights into “agonistic” effects of anti-CD137 monoclonal antibodies. Blood..

[29] Srivastava RM, Trivedi S, Concha-Benavente F, Gibson SP, Reeder C, Ferrone S (2017). CD137 stimulation enhances cetuximab-induced natural killer: dendritic cell priming of antitumor T-cell immunity in patients with head and neck cancer. Clin Cancer Res.

[30] Nijhof IS, Casneuf T, van Velzen JF, van Kessel B, Axel AE, Syed K (2016). CD38 expression and complement inhibitors affect response and resistance to daratumumab therapy in myeloma. Blood..

[31] Krejcik J, Frerichs KA, Nijhof IS, van Kessel B, van Velzen JF, Bloem AC (2017). Monocytes and granulocytes reduce CD38 expression levels on myeloma cells in patients treated with daratumumab. Clin Cancer Res.

[32] Wang Y, Zhang Y, Hughes T, Zhang J, Caligiuri MA, Benson DM (2018). Fratricide of NK cells in daratumumab therapy for multiple myeloma overcome by ex vivo expanded autologous NK cells. Clin Cancer Res.

[33] Debets JM, Van der Linden CJ, Dieteren IE, Leeuwenberg JF, Buurman WA (1988). Fc-receptor cross-linking induces rapid secretion of tumor necrosis factor (cachectin) by human peripheral blood monocytes. J Immunol.

[34] Nijhof IS, Lammerts-van Bueren JJ, van Kessel B, Andre P, Morel Y, Lokhorst HM (2015). Daratumumab-mediated lysis of primary multiple myeloma cells is enhanced in combination with the human anti-KIR antibody IPH2102 and lenalidomide. Haematologica..

[35] Fu B, Wang F, Sun R, Ling B, Tian Z, Wei H (2011). CD11b and CD27 reflect distinct population and functional specialization in human natural killer cells. Immunology..

[36] Postow MA, Manuel M, Wong P, Yuan J, Dong Z, Liu C (2015). Peripheral T cell receptor diversity is associated with clinical outcomes following ipilimumab treatment in metastatic melanoma. J Immunother Cancer..

[37] Manuel M, Tredan O, Bachelot T, Clapisson G, Courtier A, Parmentier G (2012). Lymphopenia combined with low TCR diversity (divpenia) predicts poor overall survival in metastatic breast cancer patients. Oncoimmunology..

[38] Kirsch I, Vignali M, Robins H (2015). T-cell receptor profiling in cancer. Mol Oncol.

