# Supplementary material for: Deep immune profiling of patients treated with lenalidomide and dexamethasone with or without daratumumab
Source: Leukemia. 2020 May 26;35(2):573–84. doi: 10.1038/s41375-020-0855-4 (PMC7862054; doi:10.1038/s41375-020-0855-4)
Supplement: Supplementary file 1 — Supplemental Material [file 41375_2020_855_MOESM1_ESM.pdf]

SUPPLEMENTAL MATERIALS

This supplementary file contains additional methods, tables, and figures that are relevant to the methods and conclusions of the main text, regarding the immune modifications observed with daratumumab plus lenalidomide and dexamethasone treatment in relapsed/refractory multiple myeloma patients from the phase 3 POLLUX trial.

Supplemental methods

CyTOF® sample sources and staining

Patient samples were handled as previously described[1] with minor modifications. Patients were classified as responders (ie, patients with best overall responses of partial response, very good partial response, complete response, or stringent complete response) or nonresponders (ie, patients with a best response of minimal response, stable disease, or progressive disease).

Patient whole blood (WB) samples were collected and fixed in SmartTubes® (Smart Tube, Inc. Palo Alto, CA, USA) for 10 minutes at room temperature and stored at −80°C until analysis. Purified metal-conjugated antibodies from Fluidigm (Fluidigm Corp, San Francisco, CA, USA) or labeled in-house using the Maxpar® Antibody Labeling Kit (Fluidigm) according to the manufacturer's protocol were used for cytometry by time-of-flight (CyTOF) staining and analysis. Prior to staining, samples were thawed in a 10°C water bath for 20 minutes, and red blood cells were lysed with the addition of a hypotonic thaw-lyse buffer (SmartTube®) according to the manufacturer's protocol. If complete lysis was not achieved after 2 lysis steps, a third lysis step was performed using lyse buffer 2 (SmartTube®). Samples still containing red blood cells after 3 lysis steps were further purified by CD235 depletion (MACSxpress Erythrocyte Depletion

Kit, Miltenyi Biotec, Teterow, Germany). Cells were washed with stain buffer (BD Biosciences, San Jose, CA, USA). To block surface Fc receptors, each sample received 10 µl of Human TruStain FcX (BioLegend, San Diego, CA, USA) and was incubated for 15 minutes at 4°C. Cells were then incubated with a mixture of 35 surface marker metal-conjugated antibodies (not including intracellular antibodies active caspase-3, CTLA4, and granzyme B antibodies; **Table S1**) for 45 minutes at 4°C. Regarding staining of CD38, samples were stained with fluorescein isothiocyanate α-CD38 (HuMax-003; Genmab/Janssen Research & Development), an antibody that binds to an epitope that is distinct from the epitope bound by daratumumab.[2] Samples were washed twice with staining buffer and subsequently permeabilized with 1 ml of ice cold PermWash buffer (BD Biosciences), added while vortexing and incubated for 20 minutes at 4°C. Cells were pelleted by centrifugation for 6 minutes at 600*g* and supernatant was discarded. Intracellular staining was performed using anti-caspase-3, anti–granzyme B, and anti-CTLA4 antibodies. Upon addition of intracellular antibodies, samples were incubated for 45 minutes at 4°C. Cells were washed twice with 2 ml of PermWash buffer and once with 2 ml of staining buffer. Then 1 ml of ice cold PermIII buffer (BD Biosciences) was added while vortexing and samples were incubated for 20 minutes at 4°C. Cells were washed twice with 2 ml of staining buffer, followed by 1 wash with 2 ml of phosphate-buffered saline. Cells were resuspended in phosphate-buffered saline containing Intercalator Iridium and Rhodium (Fluidigm) and barcoded using Cell-ID^{TM} 20-Plex Pd Barcoding Kit (Fluidigm), followed by a 30-minute incubation at 4°C. Cells were washed twice with 2 ml of staining buffer, followed by 1 wash with ultrapure water. Labeled cells were then dissolved in ultrapure water with EQ™ Four Element Calibration Beads at a concentration of 3.3×10E+04 beads/mL (Fluidigm) for analysis with a CyTOF C5 system (Fluidigm). Samples were run at an acquisition rate of 300 to 500 cells/min. Each

experiment was run with a maximum of 20 samples per experiment, including an aliquot of WB from a healthy donor for quality control.

Data preprocessing and quality control

Data acquired on CyTOF® were obtained in FCS file format and normalized using CyTOF® Software 6.5.358 for Stand-Alone Processing Workstations (Fluidigm). FCS files were further analyzed in Cytobank software. Manual gating was used to define granulocyte, lymphocyte, and monocyte populations and spanning-tree progression analysis of density-normalized events (SPADE) analysis was performed on lymphocyte and monocyte populations using lineage markers (**Table S1**). CD38 positivity thresholds were set with a set of separately stained WB samples with a CD38 metal minus 1 antibody panel, and natural killer cells were used as the positive compensation population. B cells were used to set thresholds of other activation markers, based on expression levels of not expressed (ie, CTLA4, PD1, and CD137) or definitively expressed (HLA-DR). Quality control assessment of 19 WB control samples from 19 different batches was performed using the earth mover's distance (EMD)[3] algorithm across channels, by clustering on the pairwise EMD matrix (calculated from cell count distributions of 150 nodes in the samples' SPADE minimum spanning trees). For individual channel quality control analysis, marker enrichment modeling (MEM)[4] was used. MEM scores were calculated using the median control (batch 10) as a reference. Scaling between −10 and 10 was defined relative to the highest maximum MEM score across all channels for the controls and samples. The channels were subsequently ranked (from left to right) by mean absolute MEM scores for the 19 WB controls in descending order.

Bootstrap P values

To obtain *P* values (*P**) corrected for the multiple dependent hypothesis tests, we used the bootstrap single-step minP multiple testing procedure.[5,6] Resampling was performed with replacement ("bootstrapping") at both the sample and cell level (100 bootstrap runs) and a down-/up-sample relative to the median cell count was generated at the cell level. The assignment of sample group labels to bootstrap samples was randomized to generate a null distribution of smallest *P* values for comparison with raw *P* values.

Imputation at the node level

For each sample with 1 or more empty node(s) in the SPADE tree, the marker signal for each empty node was assigned to the mean marker intensity of the subset of cells from ≤3 nearest neighboring nodes, when all fell within the smallest parent population of the empty node. To identify the nearest neighbors of an empty node, the shortest-path length distance between nodes was calculated as the sum of edge weights in the SPADE minimum spanning tree.

Bin analysis

Cells were sorted from smallest to the highest value, and the empirical cumulative distribution function of each condition was computed to calculate the significance of the difference between any 2 conditions. To control for differences in cell numbers collected across different samples, the contribution of each cell to the empirical cumulative distribution function was divided by the total number of cells within each sample. The difference between the empirical cumulative distribution function of the test condition to the reference condition was integrated over the difference in values observed in each group, resulting in a signed value, which was used as the

test statistic and was compared to the bootstrap distribution obtained by repeating the procedure 400 times, each time randomly assigning samples to conditions to generate a 2-tailed P value showing the significance of the difference between conditions.

NanoString analysis

RNA was extracted from aliquots of viably frozen peripheral-blood mononuclear cells using the Qiagen AllPrep kit (Qiagen, Germantown, MD, USA) according to the manufacturer's instructions. One hundred nanograms of total RNA from each of 316 samples were hybridized to the NanoString PanCancer Immune Profiling Panel. Hybridizations were purified and scanned using the NanoString nCounter Analysis System (file version: 1.7, software version: 3.1.0.1). Raw RCC files were loaded into R using the NanoStringNorm package (https://CRAN.R-project.org/package=NanoStringNorm; version 1.2.1). Samples were considered poorly quantified and removed from analysis if their median log2 raw count was ≤3 (n=15 samples), or if the R^2 value of the positive control probes was <0.95 (n=1 sample; also identified by the low expression filter). Upon removal of poorly quantified samples, the 301 samples remaining were normalized and quality controlled using procedures implemented in the NanoStringNorm package (**Figure S2**). Nine additional samples were subsequently removed during this process due to extreme positive normalization factors, backgrounds, or RNA content, resulting in a final data set of 292 samples and 730 endogenously targeted probes. Endogenous probes were also filtered to remove poorly quantified features and the postnormalization mean background probe level was calculated across the data set. A feature was preserved if it was expressed above mean background probe level in >52 samples (52 was determined by calculating three-fourths of the

number of samples in the smallest group of interest for the differential expression comparison [70 samples]).

Patients who were included in the final analysis represented a well-balanced subgroup of the entire patient population from POLLUX. Each differential expression model included a term for NanoString Plate ID to account for the batch effect (observed via Principal Variance Components Analysis). Batches were proactively randomized by treatment, time point, and response. A random-effect blocking term and the limma::duplicateCorrelation function were used to correct for increased correlation and statistically control for the pairing of samples due to repeated collection from patients across time points. Analyses were conducted using CyTOF-derived cellular abundance estimates as covariates in the model matrix (terms were used for: Memory_CD8, Naive_CD8, NK, Memory_CD4, Naive_CD4, T_{reg}, Basophils, B_cells, Monocytes, pDC, mDC, Aspecific), in addition to standard response-based differential expression tests, to remove the contribution of bulk cell type differences from the differential expression signal and allow for the focus on altered transcriptional behavior.

T-cell clonality and richness analyses

Paired samples were collected on Day 1 of Cycles 1 and 3 for 133 and 124 patients treated with daratumumab given with lenalidomide and dexamethasone and lenalidomide and dexamethasone alone, respectively, and T-cell clonality was measured by tracking the genomics T-cell receptors (TCRs) from DNA extracted from patient frozen peripheral-blood mononuclear cells. TCR rearrangements were analyzed using prequalified multiplex polymerase chain reaction assays (TR2015CRO-V-019) using primers targeting the variable and joining genes of the TCR. The

amplicon generated from the initial round of polymerase chain reaction was subsequently amplified with primers that contained the universal and adapter sequences required for DNA sequencing by Illumina® (Illumina Inc, San Diego, CA, USA).

Calculation of clonality

The extent of mono- or oligoclonal expansion was calculated and quantitated by measuring the shape of the clone frequency distribution by using the following equations.

$Entropy = H = -\sum_{i=1}^{N} p_i \log_2(p_i)$; *Clonality = 1 – (H/log₂(N)),* where N is the total number of unique TCRβ clones and p_i is the frequency of the ith unique TCRβ clone. Calculated values ranged from 0 to 1, where values approaching 1 indicated a nearly monoclonal population.

Estimation of richness

Richness of a TCRβ repertoire is the number of clones defined by unique TCRβ rearrangements. In a given sample, the number of unique TCRβ clones detected was sensitive to sampling depth. The Daley and Smith estimator was used to correct for varying sampling depths and estimate the true richness of a repertoire. The height of the horizontal asymptote on the rarefaction curve was used as an estimator for richness.

References for supplemental materials

1. Smets T, Stevenaert F, Adams H 3rd, Vanhoof G. Deep profiling of the immune system of multiple myeloma patients using cytometry by time-of-flight (CyTOF). Methods Mol Biol. 2018; 1792: 47–54.

2. van de Donk NW, Moreau P, Plesner T, Palumbo A, Gay F, Laubach JP*, et al.* Clinical efficacy and management of monoclonal antibodies targeting CD38 and SLAMF7 in multiple myeloma. Blood. 2016; 127: 681–695.

3. Qiu P. Inferring phenotypic properties from single-cell characteristics. PLoS One. 2012; 7: e37038.

4. Diggins KE, Greenplate AR, Leelatian N, Wogsland CE, Irish JM. Characterizing cell subsets using marker enrichment modeling. Nat Methods. 2017; 14: 275–278.

5. Westfall P, Young S. Resampling-based Multiple Testing: Examples and Methods for P-value Adjustment. (Wiley, New York, 1993).

6. Sham PC, Purcell SM. Statistical power and significance testing in large-scale genetic studies. Nat Rev Genet. 2014; 15: 335–346.

Supplemental Tables

Table S1. CyTOF® antibody markers

Specificity	Clone	Provider	Catalog nr	Metal isotope	Purpose
CD45*	HI30	Fluidigm	3089003B	89Y	Leukocytes
CD66b*	80H3	Abnova	custom	139La	Granulocytes
Caspase 3	C92-605	BD	559565	141Nd	Apoptotic cells
CD20*	2H7	BD	555621	142Nd	B lymphocytes
CD3*	UCHT1	BD	555329	143Nd	T lymphocytes
CD11b*	ICRF44	Fluidigm	3144001B	144Nd	Monocytes, NK
CD4*	RPA-T4	Fluidigm	3145001B	145Nd	T-helper lymphocytes
CD8*	RPA-T8	Fluidigm	3146001B	146Nd	Cytotoxic T lymphocytes
CD24*	ML5	BD	555426	147Sm	Regulatory B cells
CD34*	581	Fluidigm	3148001B	148Nd	Hematopoietic progenitor cells
CD45RO*	UCHL1	Fluidigm	3149001B	149Sm	Memory T lymphocytes
CD138	MI15	BD	551902	150Nd	Plasma cells, multiple myeloma cells
CD33*	WM53	BD	555449	151Eu	Myeloid cells
CD55	MEM118	Abcam	ab1422	152Sm	Complement inhibition
CD366 (TIM3)	344823	R&D	MAB2365	153Eu	Effector T cells, T-cell exhaustion
CD45RA*	HI100	BD	555486	154Sm	Naive T lymphocytes
CD27*	L128	Fluidigm	3155001B	155Gd	Memory B lymphocytes, T lymphocytes
CD152 (CTLA4)	L3D10	Fluidigm	3158013B	156Gd	Regulatory T cells, T-cell activation
CD137	4B4-1	Fluidigm	3158013B	158Gd	Activated NK cells
CD123*	9F5	BD	555642	159Tb	Plasmacytoid dendritic cells, basophils
CD69	FN50	BD	555529	160Dy	T-cell early activation
CD28*	CD28.2	BD	555725	161Dy	T-cell costimulation
CD11c*	Bu15	Fluidigm	3162005B	162Dy	Monocytes, myeloid dendritic cells
Granzyme B*	GB11	Thermo Fisher Sc	MA1-80734	163Dy	Activated T cells, NK cells
CD15*	W6D3	Fluidigm	3164001B	164Dy	Granulocytes
CD127*	eBioRDR5	E-Bioscience	14-1278-82	165Ho	Activated and regulatory T lymphocytes

GPRC5D	571961	R&D	custom	166Er	Plasma cells
CD19*	HIB19	BD	555409	167Er	B lymphocytes
CD269 (BCMA)	Vicky	Enzo	ALX-804-151-C100	168Er	Plasma cells
CD25*	M-A251	BD	555429	169Tm	Activated and regulatory T lymphocytes
CD279 (PD-1)	EH12-1	BD	562138	170Er	T-cell co-inhibitory receptor/exhaustion
CD14*	HCD14	Biolegend	325602	171Yb	Monocytes, macrophages
CD38	HuMax	Janssen	custom	172Yb	Daratumumab target, activation, plasma cells
VISTA	GG8	Janssen	custom	173Yb	T-cell co-inhibitory receptor
HLA-DR*	646-6	BD	555809	174Yb	Dendritic cells, monocytes, B lymphocytes, T cell activation
CD274 (PDL-1)	29E.2A3	Fluidigm	3175017B	175Lu	Activation PD-1
CD56[a]	R19-760	BD	563237	176Yb	NK and NKT cells
DNA	Intercalator	Fluidigm	201192B	191Ir, 193 Ir	Nucleated cells
CD16	3G8	Fluidigm	3209002B	209Bi	Proinflammatory monocytes, NK subset, granulocytes

BCMA, B-cell maturation antigen; CTLA4, cytotoxic T-lymphocyte–associated protein 4; GPRC5D, G-protein coupled receptor family C group 5 member D; NK, natural killer; NKT, natural killer T cell; PD-1, programmed death-1; PDL-1, programmed death ligand-1; SPADE, spanning-tree progression of density-normalized events; TIM3, T-cell immunoglobulin mucin-3; VISTA, V-domain immunoglobulin suppressor of T-cell activation.

*Markers used for SPADE annotation.

Table S2. SPADE bubble marker annotations

Population	Markers
Aspecific	No definitive lineage staining pattern
$CD45^{neg/lo}$	$CD45^{–/low}$
Basophils	$CD45^{–/dim}$, $CD123^{+}$, $HLADR^{–}$
PBMCs	$CD45^{mid\text{-}bright}$
Monos	$CD45^{+}CD14^{+}$
ProInflamm Monos	$CD16^{+}CD14^{mid}$
pDCs	$CD45^{+}CD123^{+}$, $CD11c^{low}$, $HLADR^{+}$
mDCs	$CD45^{+}CD123^{–}$,$CD11c^{high}$, $HLADR^{mid}$
$CD16^{+}$ mDC	$CD45^{+}CD123^{–}$,$CD11c^{high}$, $HLADR^{mid}$, $CD16^{+}$
$CD16^{–}$ mDC	$CD45^{+}CD123^{–}$,$CD11c^{high}$, $HLADR^{mid}$, $CD16^{–}$
Lymphocytes	All $CD45^{+}CD3^{+}$, $CD56^{+}$, $CD19^{+}$ or $CD20^{+}$ Nonmonocytes or DCs
B cells	$CD45^{+}$, $CD3^{–}$, $CD19^{+}$, $CD20^{+/–}$
Memory B cells	$CD45^{+}$, $CD3^{–}$, $CD19^{+}$, $CD20^{+/–}$, $CD27^{+}$
B_{regs}	$CD45^{+}$, $CD3^{–}$, $CD19^{+}$, $CD20^{+/–}$, $CD24^{+}CD38^{high}$
NK cells	$CD45^{+}$, $CD3^{–}$, $CD19^{–}$, $CD20^{–}$, $CD56^{+}$
$CD56^{Br}$ ($CD56^{bright}$) NK	$CD45^{+}$, $CD3^{–}$, $CD19^{–}$, $CD20^{–}$, $CD56^{bright}$
$CD56^{dim}$ NK	$CD45^{+}$, $CD3^{–}$, $CD19^{–}$, $CD20^{–}$, $CD56^{dim}$
T cells	$CD45^{+}$, $CD3^{+}$, $CD19^{–}CD20^{–}$, $CD56^{+/–}$
$CD4^{+}$ T cells	$CD45^{+}$, $CD3^{+}$, $CD4^{+}$, $CD8^{–}$
Naive $CD4^{+}$ T cells	$CD45^{+}$, $CD3^{+}$, $CD4^{+}$, $CD8^{–}$, $CD45RA^{+}$
T_{EMRA} $CD4^{+}$ T cells	$CD45^{+}$, $CD3^{+}$, $CD4^{+}$, $CD8^{–}$, $CD45RA^{+}$, $CD27^{–}$
Naive NKT $CD4^{+}$	$CD45^{+}$, $CD3^{+}$, $CD4^{+}$, $CD8^{–}$, $CD45RA^{+}$, $CD56^{+}$
Memory $CD4^{+}$ T cells	$CD45^{+}$, $CD3^{+}$,$CD4^{+}$, $CD8^{–}$, $CD45RO^{–}$
T_{EM} $CD4^{+}$ T cells	$CD45^{+}$, $CD3^{+}$, $CD4^{+}$, $CD8^{–}$, $CD45RO^{+}$, $CD27^{–}$
T_{CM} $CD4^{+}$ T cells	$CD45^{+}$, $CD3^{+}$, $CD4^{+}$, $CD8^{–}$, $CD45RO^{+}$, $CD27^{+}$
T_{regs}	$CD45^{+}$, $CD3^{+}$, $CD4^{+}$, $CD8^{–}$, $CD127^{–}CD25^{+}$
$CD8^{+}$ T cells	$CD45^{+}$, $CD3^{+}$, $CD8^{+}$,$CD4^{–}$
Naive $CD8^{+}$ T cells	$CD45^{+}$, $CD3^{+}$, $CD8^{+}$,$CD4^{–}$,$CD45RA^{+}$
T_{EMRA} $CD8^{+}$ T cells	$CD45^{+}$, $CD3^{+}$, $CD8^{+}$, $CD4^{–}$, $CD45RA^{+}$, $CD27^{–}$
Naive NKT $CD8^{+}$ T cells	$CD45^{+}$, $CD3^{+}$, $CD8^{+}$, $CD4^{–}$, $CD45RA^{+}$, $CD56^{+}$
Memory $CD8^{+}$ T cells	$CD45^{+}$, $CD3^{+}$, $CD8^{+}$, $CD4^{–}$, $CD45RO^{+}$
T_{EM} $CD8^{+}$ T cells	$CD45^{+}$, $CD3^{+}$, $CD8^{+}$, $CD4^{–}$, $CD45RO^{+}$, $CD27^{–}$
T_{CM} $CD8^{+}$ T cells	$CD45^{+}$, $CD3^{+}$, $CD8^{+}$, $CD4^{–}$, $CD45RO^{+}$, $CD27^{+}$
Memory NKT $CD8^{+}$ T cells	$CD45^{+}$, $CD3^{+}$, $CD8^{+}$, $CD4^{–}$, $CD45RO^{+}$, $CD56^{+}$

B_{regs}, regulatory B cells; DC, dendritic cells; mDC, myeloid dendritic cells; mono, monocytes; NK, natural killer; NKT, natural killer T cells; PBMC, peripheral-blood mononuclear cells; pDC, plasmacytoid dendritic cells; proinflamm mono, proinflammatory monocytes; SPADE, spanning-tree

progression analysis of density-normalized events; T_{CM}, central memory T cells; T_{EM}, effector memory T cells; T_{EMRA}, effector memory $CD45RA^+$ $CD4^+$ T cells; T_{regs}, regulatory T cells.

Table S3. Corresponding LIMMA anaylsis for Figures S3A and S3B.
(A) D-Rd at C3D1 versus C1D1. (B) Rd at C3D1 versus C1D1. (C) D-Rd at C3D1 versus C1D1, CyTOF corrected. (D) Rd at C3D1 versus C1D1, CyTOF corrected.

Name	Accession	missing_frac	logFC	AveExpr	t	P.Value	adj.P.Val	B
**A. D-Rd at C3D1 versus C1D1**								
SH2D1B	NM_053282.4	0.205	-3.41681	3.706056	-14.9021	5.78E-37	2.83E-34	73.30119
KLRF1	NM_016523.1	0.086	-3.00283	5.228966	-14.1678	2.30E-34	5.64E-32	67.38061
KLRC1	NM_002259.3	0.079	-2.19502	4.143517	-10.4676	1.03E-21	1.69E-19	38.58832
NCAM1	NM_000615.5	0.017	-1.53667	4.312272	-10.1965	7.80E-21	9.55E-19	36.59592
KLRC2	NM_002260.3	0.134	-1.99793	3.811944	-8.45919	1.79E-15	1.75E-13	24.4365
NCR1	NM_004829.5	0.017	-1.35513	5.426898	-7.80681	1.34E-13	1.09E-11	20.19922
CCR5	NM_000579.1	0.462	1.740521	1.57749	7.740926	2.04E-13	1.43E-11	19.7829
TXK	NM_003328.1	0.202	-1.65782	2.831012	-7.63704	3.97E-13	2.43E-11	19.13101
TNFRSF18	NM_004195.2	0.247	-1.48002	2.385066	-6.88376	4.15E-11	2.26E-09	14.57908
FEZ1	NM_005103.4	0.414	-1.44229	1.635389	-6.54278	3.07E-10	1.50E-08	12.62641
AKT3	NM_181690.1	0.003	-0.72754	6.132502	-6.24445	1.67E-09	7.42E-08	10.97699
GNLY	NM_006433.2	0.003	-1.00535	9.647454	-6.01491	5.90E-09	2.41E-07	9.746945
KIR_Activating_Subgroup_2	NM_014512.1	0.349	-1.49462	2.233523	-5.77725	2.11E-08	7.75E-07	8.51051
CD8B	NM_004931.3	0.024	1.062014	5.952351	5.768002	2.21E-08	7.75E-07	8.463154
PTPRC	NM_080921.3	0	0.655771	9.119697	5.752554	2.40E-08	7.85E-07	8.384219
S100A12	NM_005621.1	0	0.792894	10.46119	5.692146	3.30E-08	1.01E-06	8.077119
CCRL2	NM_003965.4	0.072	1.083262	3.840413	5.650747	4.09E-08	1.14E-06	7.868118
CD84	NM_001184879.1	0	0.429798	6.841624	5.64538	4.21E-08	1.14E-06	7.841114
CD8A	NM_001768.5	0	0.80596	7.865574	5.61845	4.84E-08	1.25E-06	7.705905
CD70	NM_001252.2	0.346	1.251414	1.907375	5.565217	6.36E-08	1.56E-06	7.440137
GZMK	NM_002104.2	0.01	1.054386	6.858668	5.515896	8.19E-08	1.91E-06	7.195684
PDGFRB	NM_002609.3	0.349	-1.14096	1.901005	-5.32375	2.16E-07	4.80E-06	6.259943
PVR	NM_006505.3	0.182	1.136021	2.843678	5.247303	3.15E-07	6.71E-06	5.895086
MX1	NM_002462.2	0	-0.77543	7.738464	-5.121	5.82E-07	1.19E-05	5.301714

ITGAM	NM_000632.3	0	0.483722	8.143104	5.061426	7.76E-07	1.52E-05	5.025928
CD247	NM_198053.1	0.007	-0.68805	7.876366	-5.00378	1.02E-06	1.92E-05	4.761617
KIR_Inhibiting_Subgroup_1	NM_014218.2	0.243	-1.14847	2.723496	-4.80752	2.55E-06	4.64E-05	3.880651
LAG3	NM_002286.5	0.065	1.086793	4.59753	4.789119	2.78E-06	4.86E-05	3.799551
CR1	NM_000651.4	0	0.740897	8.079674	4.745726	3.39E-06	5.73E-05	3.60937
KIR_Activating_Subgroup_1	NM_001083539.1	0.469	-1.22277	1.847964	-4.70583	4.06E-06	6.64E-05	3.435808
KLRB1	NM_002258.2	0.007	-0.71353	7.909012	-4.50459	9.95E-06	0.000157	2.57935
IFITM1	NM_003641.3	0	-0.45607	10.38768	-4.26469	2.78E-05	0.000424	1.600485
ITGB2	NM_000211.2	0	0.32014	10.81662	4.258522	2.85E-05	0.000424	1.575935
IRF4	NM_002460.1	0.003	-0.61667	6.314729	-4.09722	5.55E-05	0.0008	0.94487
ISG15	NM_005101.3	0	-0.69021	7.013594	-4.08937	5.73E-05	0.000802	0.914705
ABCB1	NM_000927.3	0.092	-0.8762	3.909035	-4.04055	6.98E-05	0.00095	0.728275
IL17RA	NM_014339.6	0.003	0.472284	7.084215	4.017942	7.64E-05	0.001005	0.642586
CD44	NM_001001392.1	0	0.296753	11.27305	4.012959	7.79E-05	0.001005	0.62376
KLRG1	NM_005810.3	0.051	0.933907	5.268751	3.98302	8.78E-05	0.00109	0.511088
CYBB	NM_000397.3	0	0.458037	9.699284	3.979712	8.90E-05	0.00109	0.498682
CD3D	NM_000732.4	0.003	0.570789	8.308636	3.950701	9.98E-05	0.001193	0.390294
CD36	NM_001001548.2	0	0.524659	9.040517	3.918928	0.000113	0.001319	0.272398
SIGLEC1	NM_023068.3	0.182	-1.29751	3.955035	-3.84717	0.00015	0.001704	0.009272
MAF	NM_005360.4	0.021	0.599062	5.451789	3.834556	0.000157	0.001721	-0.03655
IFIT1	NM_001548.3	0.079	-1.06272	4.665226	-3.83281	0.000158	0.001721	-0.04288
ENTPD1	NM_001098175.1	0.092	0.778952	3.750198	3.75999	0.000209	0.0022	-0.30458
IRF7	NM_001572.3	0	-0.40975	7.657972	-3.75718	0.000211	0.0022	-0.31458
BCL6	NM_001706.2	0	0.479732	9.486685	3.702348	0.000259	0.002571	-0.50853
KLRD1	NM_002262.3	0.014	-0.72143	6.719953	-3.69959	0.000262	0.002571	-0.51821
TARP	NM_001003799.1	0.003	0.530529	6.887268	3.699418	0.000262	0.002571	-0.51882
TNFRSF14	NM_003820.2	0	0.186072	7.564073	3.643948	0.000323	0.003091	-0.71226
LTBR	NM_002342.1	0.007	0.42619	6.519281	3.639401	0.000328	0.003091	-0.728
BST1	NM_004334.2	0	0.400438	8.429325	3.631924	0.000337	0.003118	-0.75384
CD2	NM_001767.3	0.072	0.793883	4.100804	3.624966	0.000346	0.003139	-0.77785

CFP	NM_002621.2	0	0.331617	8.896465	3.606935	0.00037	0.003294	-0.83987
BATF	NM_006399.3	0.021	0.527219	4.187868	3.576466	0.000413	0.003523	-0.94402
KIR3DL2	NM_006737.2	0.253	-0.84899	2.487052	-3.5754	0.000415	0.003523	-0.94767
MAPK3	NM_001040056.1	0	0.31298	6.641203	3.574029	0.000417	0.003523	-0.95232
JAK3	NM_000215.2	0	0.310342	7.79399	3.562975	0.000434	0.003546	-0.98989
SLC11A1	NM_000578.2	0	0.410665	8.417996	3.562954	0.000434	0.003546	-0.98996
IL6R	NM_000565.2	0	0.32378	6.492918	3.551058	0.000453	0.003622	-1.03027
AMICA1	NM_153206.2	0	0.483811	8.896182	3.547248	0.00046	0.003622	-1.04315
CREB5	NM_182898.2	0.007	0.636811	6.394364	3.543623	0.000466	0.003622	-1.0554
ANXA1	NM_000700.1	0	0.331873	11.06019	3.53887	0.000474	0.003627	-1.07144
HLA-DOB	NM_002120.3	0.01	-0.63928	5.225432	-3.52261	0.000502	0.003734	-1.12616
CD40	NM_001250.4	0.021	0.612597	5.137436	3.522345	0.000503	0.003734	-1.12705
CD79B	NM_021602.2	0	-0.57396	7.068639	-3.5136	0.000519	0.003796	-1.15641
PSMB10	NM_002801.2	0	0.227266	8.926977	3.43114	0.000696	0.005017	-1.42974
ITGB1	NM_033666.2	0	0.25672	9.274698	3.412549	0.000743	0.005279	-1.49055
LCP1	NM_002298.4	0	0.263542	11.29735	3.406474	0.000759	0.005286	-1.51035
CD33	NM_001177608.1	0.024	0.555835	5.324418	3.40402	0.000766	0.005286	-1.51834
MARCO	NM_006770.3	0.031	0.704341	5.582365	3.373583	0.000852	0.005798	-1.61701
CD24	NM_013230.2	0.007	-0.65722	5.745172	-3.339	0.000961	0.006447	-1.72815
S100A8	NM_002964.3	0	0.557673	12.77144	3.297923	0.001106	0.007325	-1.85877
CD46	NM_172350.1	0	0.184901	8.729013	3.285543	0.001154	0.007496	-1.89784
CD163	NM_004244.4	0.007	0.652912	7.064932	3.283346	0.001163	0.007496	-1.90476
CCL5	NM_002985.2	0	0.379724	10.74778	3.213697	0.001471	0.009306	-2.12194
FCER1G	NM_004106.1	0	0.304969	10.93316	3.211651	0.001481	0.009306	-2.12825
CD7	NM_006137.6	0.007	-0.35531	6.90442	-3.20036	0.001538	0.009542	-2.16302
CTSS	NM_004079.3	0	0.296626	12.66577	3.188827	0.001599	0.009792	-2.19844
IL18	NM_001562.2	0.024	0.492262	5.163597	3.162812	0.001743	0.010455	-2.27788
CXCL1	NM_001511.1	0.144	0.950258	4.529862	3.161534	0.00175	0.010455	-2.28176
STAT5B	NM_012448.3	0	0.29585	6.965891	3.157978	0.001771	0.010455	-2.29257
CD14	NM_000591.2	0	0.454038	9.392181	3.129524	0.001945	0.011346	-2.37864

TLR4	NM_138554.2	0.003	0.429478	6.63778	3.102603	0.002124	0.012245	-2.45941
MAP2K1	NM_002755.2	0	0.267301	7.536886	3.081023	0.002278	0.012982	-2.52369
CD47	NM_001777.3	0	-0.14662	8.84671	-3.05494	0.002479	0.013961	-2.6008
LRP1	NM_002332.2	0	0.312869	7.60475	3.050507	0.002514	0.014	-2.61386
ICAM3	NM_002162.3	0	0.181665	9.390235	3.026442	0.002716	0.014954	-2.68439
PRKCE	NM_005400.2	0.151	-0.63034	3.082404	-3.00676	0.002892	0.015745	-2.74167
KIR_Inhibiting_Subgroup_2	NM_014511.3	0.466	-0.70009	1.691103	-3.001	0.002945	0.01586	-2.75837
MEFV	NM_000243.2	0.003	0.406438	7.319955	2.992718	0.003024	0.015956	-2.78234
IFNG	NM_000619.2	0.007	0.55437	6.625883	2.99223	0.003028	0.015956	-2.78375
PYCARD	NM_013258.3	0	0.28966	7.013016	2.98793	0.00307	0.016003	-2.79616
ITGA5	NM_002205.2	0	0.225765	7.295539	2.938682	0.003584	0.018487	-2.93715
OAS3	NM_006187.2	0.024	-0.67505	5.979037	-2.92645	0.003723	0.018944	-2.97182
PIK3CD	NM_005026.3	0	0.269563	7.459838	2.924165	0.00375	0.018944	-2.97829
PAX5	NM_016734.1	0.178	-0.81488	3.896755	-2.90305	0.004004	0.02002	-3.03778
NLRC5	NM_032206.4	0.003	0.327928	7.113546	2.895917	0.004093	0.02026	-3.05779
CD27	NM_001242.4	0.031	0.552271	5.303214	2.868439	0.004454	0.021588	-3.13443
REPS1	NM_001128617.2	0	-0.23993	7.134522	-2.86672	0.004478	0.021588	-3.13921
TYK2	NM_003331.3	0	0.222789	6.65378	2.865558	0.004494	0.021588	-3.14243
BAX	NM_138761.3	0	0.14886	8.163919	2.84639	0.004765	0.022668	-3.19543
CD6	NM_006725.3	0.014	0.434557	6.383183	2.829149	0.005021	0.023657	-3.24283
NOD2	NM_022162.1	0	0.30178	6.60489	2.823654	0.005105	0.023826	-3.25787
PRF1	NM_005041.3	0.007	-0.49842	8.437403	-2.80111	0.005465	0.025263	-3.31933
JAK2	NM_004972.2	0	0.249047	7.932236	2.796542	0.005541	0.025358	-3.33171
NCF4	NM_000631.4	0	0.371764	7.36641	2.793273	0.005595	0.025358	-3.34057
CAMP	NM_004345.3	0.253	-0.81128	2.845462	-2.78954	0.005658	0.025358	-3.35066
XCL2	NM_003175.3	0.038	-0.59723	5.62487	-2.78752	0.005693	0.025358	-3.35614
NFATC1	NM_172389.1	0.007	-0.2985	5.23277	-2.76644	0.006063	0.026764	-3.4129
APP	NM_000484.3	0	0.23494	7.901192	2.761502	0.006153	0.026918	-3.42615
HCK	NM_002110.2	0	0.320142	9.069937	2.748681	0.006392	0.027715	-3.46042
CD99	NM_002414.3	0	0.211892	10.01378	2.717751	0.007003	0.030091	-3.54249

MYD88	NM_002468.3	0	0.238502	8.563028	2.714866	0.007062	0.030091	-3.5501
GPI	NM_000175.2	0	0.158869	7.81144	2.709321	0.007178	0.030321	-3.5647
TOLLIP	NM_019009.2	0.01	0.297228	5.198327	2.690139	0.007592	0.031796	-3.615
MAP2K2	NM_030662.2	0	0.17964	8.356256	2.685174	0.007703	0.031986	-3.62797
GTF3C1	NM_001520.3	0.007	0.246212	5.445361	2.677614	0.007874	0.032423	-3.64766
SH2D1A	NM_001114937.2	0.024	0.465125	5.74523	2.67069	0.008034	0.032805	-3.66566
LAMP1	NM_005561.3	0	0.145852	8.754433	2.650769	0.00851	0.034463	-3.71719
CD3G	NM_000073.2	0.021	0.499929	6.111588	2.645475	0.008641	0.034706	-3.73082
GZMH	NM_033423.3	0.007	0.502992	8.230464	2.619782	0.009302	0.036877	-3.7966
CD38	NM_001775.2	0.007	-0.4401	5.552394	-2.61708	0.009374	0.036877	-3.80349
CLEC4A	NM_194448.2	0	0.249446	7.873964	2.615834	0.009407	0.036877	-3.80666
CD274	NM_014143.3	0.469	0.643583	1.515544	2.61043	0.009553	0.037152	-3.8204
ICOSLG	NM_015259.4	0.055	-0.52573	4.973823	-2.60391	0.009732	0.037551	-3.83695
FCGR1A	NM_000566.3	0.202	0.549029	2.38127	2.562312	0.010947	0.041905	-3.94155
PLAUR	NM_001005376.1	0	0.326902	9.382228	2.553371	0.011225	0.042013	-3.96383
LILRB2	NM_005874.1	0	0.245869	7.646844	2.552178	0.011262	0.042013	-3.96679
TLR8	NM_016610.2	0.014	0.36324	5.396971	2.551389	0.011287	0.042013	-3.96876
DUSP6	NM_001946.2	0	0.361146	8.345518	2.550419	0.011318	0.042013	-3.97116
CLEC4C	NM_203503.1	0.455	-0.58754	1.61876	-2.5388	0.011691	0.043073	-3.99996
IKBKG	NM_003639.2	0.003	0.239628	6.347981	2.529468	0.011999	0.043878	-4.02301
IL2RB	NM_000878.2	0.014	-0.40458	7.075061	-2.52224	0.012243	0.044436	-4.04081
LILRA1	NM_006863.1	0.038	0.503271	5.249274	2.508556	0.012716	0.045698	-4.07436
TNFSF12	NM_003809.2	0	0.219304	7.404735	2.506818	0.012777	0.045698	-4.07861
CTSW	NM_001335.3	0.003	-0.3448	7.625144	-2.49979	0.013027	0.046256	-4.09577
TAL1	NM_003189.2	0.058	0.57327	4.306962	2.474891	0.013949	0.049172	-4.15616
CTSH	NM_004390.3	0	0.239789	8.795855	2.469389	0.01416	0.049319	-4.16943
CD4	NM_000616.4	0	0.255158	7.792406	2.468574	0.014192	0.049319	-4.1714
CD3E	NM_000733.2	0.003	0.336242	7.960763	2.45971	0.014539	0.049957	-4.19271
FPR2	NM_001462.3	0.041	0.544979	5.147605	2.456968	0.014648	0.049957	-4.19929
ITGAX	NM_000887.3	0	0.224065	9.114923	2.456127	0.014681	0.049957	-4.2013

CSF2RB	NM_000395.2	0	0.352959	8.605077	2.428805	0.015809	0.053423	-4.26644
CCL3	NM_002983.2	0	-0.33221	9.06783	-2.41761	0.016293	0.054681	-4.29294
SBNO2	NM_014963.2	0.003	0.324087	6.477355	2.40959	0.016647	0.05549	-4.31183
ISG20	NM_002201.4	0	-0.24452	7.838453	-2.39845	0.017151	0.056784	-4.338
TBX21	NM_013351.1	0.007	-0.36026	6.936496	-2.3861	0.017725	0.058291	-4.36686
CXCL2	NM_002089.3	0.058	0.743106	6.138127	2.380986	0.017968	0.058441	-4.37876
GZMA	NM_006144.2	0.01	0.476721	8.236576	2.377494	0.018135	0.058441	-4.38688
CCR2	NM_001123041.2	0.175	0.694222	3.44741	2.377374	0.018141	0.058441	-4.38716
STAT4	NM_003151.2	0.003	-0.31897	6.168636	-2.37515	0.018248	0.058441	-4.39232
CD55	NM_000574.3	0.264	0.5755	2.574407	2.369065	0.018545	0.059006	-4.40643
TNFSF4	NM_003326.2	0.007	0.385024	4.998437	2.365422	0.018724	0.059193	-4.41486
CCL4	NM_002984.2	0	-0.43388	7.435982	-2.35957	0.019016	0.05973	-4.42838
BCL2	NM_000657.2	0	-0.25148	5.932649	-2.35611	0.01919	0.059892	-4.43634
TICAM2	NM_021649.4	0.158	0.530881	3.007295	2.347055	0.019653	0.060949	-4.45717
RUNX1	NM_001754.4	0	0.202637	6.612867	2.342903	0.019869	0.06123	-4.46669
ECSIT	NM_001142464.2	0.003	0.264785	5.140958	2.33816	0.020117	0.061609	-4.47754
MAPK14	NM_001315.1	0	0.299062	8.18495	2.331188	0.020488	0.061995	-4.49346
GZMB	NM_004131.3	0.003	-0.35074	8.566658	-2.33103	0.020496	0.061995	-4.49383
LCK	NM_005356.2	0.003	0.285389	7.600179	2.320101	0.021089	0.063397	-4.51868
CTLA4	NM_005214.3	0.01	0.365911	5.860546	2.303732	0.022006	0.065749	-4.5557
FCGR3A	NM_000569.6	0	-0.32026	8.819493	-2.27467	0.023719	0.070437	-4.62082
PRKCD	NM_006254.3	0	0.237448	8.691483	2.269047	0.024063	0.07103	-4.63332
CD160	NM_007053.2	0.171	-0.62107	3.418159	-2.26458	0.02434	0.071418	-4.64325
SYK	NM_003177.3	0	0.2308	7.845749	2.26224	0.024486	0.071418	-4.64842
BTK	NM_000061.1	0	0.188386	6.738055	2.236171	0.026166	0.075303	-4.70585
CCL3L1	NM_021006.4	0	-0.30834	8.932008	-2.23611	0.026171	0.075303	-4.70599
TNFRSF1A	NM_001065.2	0	0.208707	7.604868	2.234478	0.026279	0.075303	-4.70955
TLR6	NM_006068.2	0.024	0.347632	4.722826	2.2291	0.026639	0.075891	-4.72131
MAPK1	NM_138957.2	0	0.163464	8.063744	2.226368	0.026824	0.075973	-4.72727
CD68	NM_001251.2	0	0.198991	9.788476	2.224096	0.026978	0.075973	-4.73223

PECAM1	NM_000442.3	0	0.179892	9.144289	2.198016	0.028806	0.08009	-4.78874
CXCR3	NM_001504.1	0.11	0.475396	3.638518	2.194281	0.029077	0.08009	-4.79678
ARG1	NM_000045.2	0.408	0.691967	2.243983	2.194138	0.029087	0.08009	-4.79708
FCGR2A	NM_021642.3	0	0.28213	9.886013	2.194039	0.029094	0.08009	-4.7973
NLRP3	NM_001079821.2	0	0.291763	7.913347	2.186845	0.029621	0.081086	-4.81275
PSMB8	NM_004159.4	0	0.143517	8.709897	2.178847	0.030217	0.082258	-4.82986
SLAMF1	NM_003037.2	0.205	0.489197	2.599121	2.167578	0.031074	0.084124	-4.85388
F13A1	NM_000129.3	0	0.333896	9.569633	2.14357	0.03297	0.088767	-4.90464
TNFRSF13C	NM_052945.3	0.045	-0.53199	6.038428	-2.1378	0.033441	0.08954	-4.91675
C3AR1	NM_004054.2	0.147	0.556983	3.763189	2.097793	0.036863	0.097599	-4.99991
FUT7	NM_004479.3	0.058	0.501392	4.629216	2.097493	0.03689	0.097599	-5.00053
CD53	NM_001040033.1	0	0.149053	10.1139	2.095727	0.037048	0.097599	-5.00416
THBD	NM_000361.2	0.003	0.439923	8.640481	2.090443	0.037523	0.098323	-5.01502
DPP4	NM_001935.3	0.366	0.461306	1.721784	2.084983	0.03802	0.098805	-5.02621
CD5	NM_014207.2	0.007	0.28897	7.202144	2.084002	0.03811	0.098805	-5.02822
RIPK2	NM_003821.5	0	0.250255	8.656458	2.074521	0.038989	0.099953	-5.04758
RPS6	NM_001010.2	0	-0.1768	12.82758	-2.07334	0.0391	0.099953	-5.04999
ITGA2B	NM_000419.3	0	0.365633	7.925738	2.072638	0.039165	0.099953	-5.05141
TLR5	NM_003268.3	0.202	0.531728	3.00475	2.064061	0.039978	0.101348	-5.06884
FYN	NM_002037.3	0	0.164473	8.537363	2.062514	0.040126	0.101348	-5.07197
CKLF	NM_181640.2	0	0.291154	7.308658	2.059725	0.040394	0.101502	-5.07762
TLR2	NM_003264.3	0	0.266334	7.639966	2.049766	0.041364	0.103409	-5.09772
ATG7	NM_001136031.2	0.014	0.358224	5.433937	2.035535	0.042784	0.106417	-5.12629
IL1R2	NM_173343.1	0.027	0.675714	5.976301	2.026329	0.043725	0.108208	-5.14466
TNFRSF9	NM_001561.4	0.329	0.432551	1.935197	2.014698	0.044938	0.110212	-5.16776
SMAD2	NM_005901.5	0	0.138294	6.605892	2.014261	0.044985	0.110212	-5.16862
CD9	NM_001769.2	0	0.290111	6.234418	2.011642	0.045262	0.11034	-5.17381
SIGIRR	NM_021805.2	0.007	0.233946	6.441101	2.005464	0.045922	0.111395	-5.18601
CASP1	NM_001223.3	0	0.202287	8.02859	1.993983	0.04717	0.113353	-5.20858
IKBKE	NM_014002.2	0.027	0.386498	5.211535	1.993788	0.047192	0.113353	-5.20896

NUP107	NM_020401.2	0.003	-0.15193	5.14324	-1.9827	0.048425	0.115603	-5.23064
LGALS3	NM_001177388.1	0	0.245447	9.566068	1.981146	0.0486	0.115603	-5.23367
PIK3CG	NM_002649.2	0.003	0.181245	7.079316	1.973193	0.049504	0.117184	-5.24914
LILRA5	NM_181879.2	0	0.230913	9.279045	1.967672	0.05014	0.118119	-5.25984
TNFSF13B	NM_006573.4	0.003	0.296887	6.505515	1.96088	0.050932	0.118925	-5.27296
BTLA	NM_181780.2	0.034	-0.39411	4.895995	-1.96057	0.050968	0.118925	-5.27355
BLNK	NM_013314.2	0.432	-0.46884	1.715381	-1.95816	0.051252	0.11902	-5.2782
MERTK	NM_006343.2	0.171	0.43155	2.826829	1.936018	0.05392	0.124628	-5.32062
CSF1	NM_000757.4	0.236	0.519842	2.980229	1.925146	0.055273	0.127153	-5.34128
LILRB3	NM_006864.2	0	0.287106	7.73877	1.869507	0.062646	0.143443	-5.44523
IL2RA	NM_000417.1	0.318	-0.39047	1.886076	-1.86306	0.063552	0.144839	-5.45708
MIF	NM_002415.1	0	-0.13941	8.121836	-1.85721	0.064382	0.145623	-5.4678
CD86	NM_175862.3	0.024	0.314964	5.845976	1.856451	0.06449	0.145623	-5.46919
IGF2R	NM_000876.1	0.003	0.217785	6.933908	1.850033	0.065413	0.146586	-5.48091
STAT6	NM_003153.3	0	0.177015	9.220851	1.84933	0.065515	0.146586	-5.48219
HLA-DRB3	NM_022555.3	0	0.16786	11.31087	1.834446	0.0677	0.150786	-5.5092
ANP32B	NM_006401.2	0	0.159597	8.430959	1.831395	0.068155	0.151113	-5.51472
IFI27	NM_005532.3	0.202	-0.84351	4.944098	-1.82081	0.069755	0.153963	-5.53377
CD63	NM_001780.4	0	0.187677	9.740343	1.815047	0.070638	0.154492	-5.5441
MAP2K4	NM_003010.2	0.171	0.402001	2.860254	1.813294	0.070909	0.154492	-5.54723
LAMP2	NM_001122606.1	0	0.138036	7.314486	1.81309	0.07094	0.154492	-5.54759
SELL	NR_029467.1	0	0.231525	9.719688	1.807002	0.071887	0.155862	-5.55846
CARD11	NM_032415.2	0	-0.17702	6.856218	-1.8031	0.072499	0.156496	-5.5654
CCR4	NM_005508.4	0.062	0.360719	4.225344	1.792409	0.074199	0.159463	-5.58435
STAT3	NM_139276.2	0	0.131285	8.852237	1.790312	0.074536	0.159488	-5.58806
CCR7	NM_001838.2	0.113	-0.40158	3.873809	-1.76406	0.078865	0.167157	-5.63407
CSF1R	NM_005211.2	0.164	0.448694	3.669342	1.763889	0.078894	0.167157	-5.63437
BCL10	NM_003921.2	0	-0.1225	8.121733	-1.76241	0.079144	0.167157	-5.63694
MAVS	NM_020746.3	0.031	0.281078	4.40858	1.758621	0.079787	0.167793	-5.64352
CD1D	NM_001766.3	0	0.196275	6.7726	1.739177	0.083156	0.174129	-5.67708

IL12RB1	NM_005535.1	0	0.147886	5.68807	1.734363	0.084007	0.175164	-5.68533
PPBP	NM_002704.2	0	0.291871	11.85189	1.731233	0.084565	0.17558	-5.69068
CD59	NM_000611.4	0	-0.25373	6.047519	-1.72094	0.08642	0.17799	-5.70822
ABL1	NM_005157.3	0.014	-0.20131	4.733803	-1.71954	0.086674	0.17799	-5.71058
CSF3R	NM_156038.2	0	0.266759	10.23874	1.718767	0.086815	0.17799	-5.7119
PTGS2	NM_000963.1	0.034	0.457425	6.713789	1.71658	0.087215	0.178064	-5.71561
CD58	NM_001779.2	0	0.134737	7.622745	1.714148	0.087662	0.178233	-5.71973
MME	NM_000902.2	0.216	0.601901	4.285765	1.706816	0.089018	0.180244	-5.73211
CMKLR1	NM_004072.1	0.301	-0.46717	2.531657	-1.6984	0.090597	0.182685	-5.74625
HLA-DMA	NM_006120.3	0	0.141123	8.650773	1.684709	0.093213	0.187189	-5.76912
ITGAL	NM_002209.2	0	0.137909	8.40379	1.668326	0.096423	0.191015	-5.79624
ICOS	NM_012092.2	0.048	0.346148	5.292234	1.667893	0.096509	0.191015	-5.79696
NFKB2	NM_002502.2	0	-0.2444	9.49988	-1.66707	0.096672	0.191015	-5.79831
CD200	NM_005944.5	0.226	-0.3983	2.696199	-1.66705	0.096677	0.191015	-5.79835
CD83	NM_004233.3	0	-0.20491	10.15734	-1.66437	0.097212	0.191301	-5.80276
ITGB3	NM_000212.2	0.003	0.324411	6.991195	1.659975	0.098093	0.192262	-5.80997
TICAM1	NM_014261.1	0.003	-0.18054	6.452073	-1.63835	0.102525	0.200149	-5.8452
TNFRSF10C	NM_003841.3	0.106	0.524647	5.144526	1.630503	0.104174	0.20256	-5.85788
INPP5D	NM_005541.3	0	0.101407	7.592274	1.62661	0.104999	0.203358	-5.86414
MAP3K1	NM_005921.1	0	-0.09879	8.030733	-1.61473	0.107549	0.207477	-5.88316
CD79A	NM_001783.3	0.003	-0.32874	7.378975	-1.60655	0.109334	0.210093	-5.89618
CD81	NM_004356.3	0	0.108501	9.013876	1.600666	0.110633	0.211759	-5.90552
SELPLG	NM_001206609.1	0	0.217235	7.156874	1.571082	0.117347	0.223736	-5.9519
CARD9	NM_052813.4	0.089	0.337109	4.2204	1.564369	0.118915	0.225263	-5.96231
STAT1	NM_007315.2	0	0.176626	8.891621	1.563336	0.119157	0.225263	-5.9639
TXNIP	NM_006472.1	0	-0.13231	12.29742	-1.56177	0.119527	0.225263	-5.96633
CXCR4	NM_003467.2	0	-0.16252	11.62269	-1.55761	0.120509	0.226243	-5.97274
PSMB9	NM_002800.4	0	0.176752	8.658391	1.553184	0.121563	0.227351	-5.97955
LILRB1	NM_001081637.1	0.007	0.249265	6.524287	1.547335	0.122966	0.228235	-5.98852
CYLD	NM_015247.1	0	-0.11888	8.50695	-1.54552	0.123405	0.228235	-5.9913

IRF3	NM_001571.5	0.01	0.166218	5.322492	1.5454	0.123433	0.228235	-5.99148
CXCL3	NM_002090.2	0.267	0.533637	3.587643	1.540035	0.124736	0.229776	-5.99966
C2	NM_000063.3	0.243	0.378901	2.578399	1.532053	0.126693	0.232507	-6.01179
TNFRSF8	NM_152942.2	0.373	-0.32676	1.712385	-1.5224	0.129092	0.236026	-6.02638
CASP3	NM_032991.2	0.007	-0.16381	5.030588	-1.51277	0.131521	0.239573	-6.04084
BST2	NM_004335.2	0	-0.15642	6.971434	-1.50811	0.132709	0.240843	-6.04781
IL10RA	NM_001558.2	0	0.099196	9.67183	1.501943	0.134293	0.242818	-6.05699
CD244	NM_016382.2	0.017	0.227955	6.020149	1.490724	0.137214	0.247187	-6.0736
CHUK	NM_001278.3	0	0.140904	6.720205	1.484485	0.138859	0.249235	-6.08279
HLA-DRA	NM_019111.3	0	0.130451	12.5209	1.470147	0.142699	0.255191	-6.10375
TAP2	NM_000544.3	0	0.12125	7.793236	1.44842	0.148672	0.264907	-6.13514
SMAD3	NM_005902.3	0	-0.16599	6.376146	-1.44312	0.150158	0.266585	-6.14273
TIGIT	NM_173799.2	0.096	0.321056	3.561425	1.44107	0.150736	0.266645	-6.14566
JAM3	NM_032801.3	0.027	0.310485	4.903156	1.430013	0.153882	0.270679	-6.16138
CTSL	NM_001912.4	0.205	0.373504	2.912495	1.428553	0.154301	0.270679	-6.16345
IL18R1	NM_003855.2	0.106	0.364047	3.790822	1.427258	0.154674	0.270679	-6.16528
RUNX3	NM_004350.1	0	-0.15498	8.954162	-1.41944	0.156937	0.273363	-6.1763
CEBPB	NM_005194.2	0	0.15375	9.52179	1.418114	0.157323	0.273363	-6.17816
PIN1	NM_006221.2	0	0.093019	6.588471	1.405379	0.161071	0.278886	-6.19597
NOTCH1	NM_017617.3	0.007	0.177519	6.163288	1.401063	0.162356	0.279916	-6.20197
LTB	NM_002341.1	0	0.168483	9.500478	1.39955	0.162808	0.279916	-6.20407
ETS1	NM_005238.3	0	-0.1474	9.109918	-1.39095	0.165397	0.282395	-6.21595
PLAU	NM_002658.2	0.226	0.552814	4.195883	1.390934	0.165403	0.282395	-6.21598
LAIR2	NM_002288.3	0.014	-0.22649	4.845662	-1.38641	0.166779	0.283756	-6.22221
JAK1	NM_002227.1	0	-0.05898	9.228152	-1.38147	0.168288	0.285333	-6.22898
POU2AF1	NM_006235.2	0.455	-0.36936	1.816663	-1.37777	0.169428	0.285652	-6.23404
CCR1	NM_001295.2	0.007	0.220317	6.178898	1.377075	0.169642	0.285652	-6.23499
IKBKB	NM_001556.1	0.024	0.227596	4.661722	1.370672	0.171628	0.287762	-6.2437
CD180	NM_005582.2	0.164	-0.35221	3.296288	-1.36771	0.172553	0.287762	-6.24772
HLA-DPB1	NM_002121.4	0	0.128797	10.31264	1.367376	0.172657	0.287762	-6.24818

ICAM4	NM_001039132.1	0.068	0.27321	3.664074	1.348974	0.178488	0.296471	-6.27295
SOCS1	NM_003745.1	0.024	0.252825	5.279282	1.335089	0.182984	0.302912	-6.29142
LTF	NM_002343.2	0.075	-0.43479	5.321943	-1.32866	0.185096	0.304678	-6.29991
IL6	NM_000600.1	0.027	0.254807	5.194713	1.327407	0.185508	0.304678	-6.30156
CD97	NM_078481.2	0	0.103223	10.53952	1.324908	0.186334	0.304678	-6.30484
TAB1	NM_153497.2	0.027	0.201001	4.727958	1.324294	0.186537	0.304678	-6.30565
TLR1	NM_003263.3	0	0.133115	7.545529	1.317599	0.188767	0.307158	-6.31442
MFGE8	NM_001114614.1	0.055	-0.25521	3.704272	-1.31598	0.18931	0.307158	-6.31653
PSEN2	NM_000447.2	0.058	-0.22722	3.557444	-1.30246	0.193881	0.313536	-6.33408
FCGR2B	NM_001002273.1	0.048	-0.2737	5.088996	-1.29084	0.197874	0.318942	-6.34902
CD74	NM_001025159.1	0	0.097469	12.72202	1.285973	0.199566	0.320614	-6.35525
HLA-DMB	NM_002118.3	0	0.128575	8.280356	1.282529	0.200769	0.321446	-6.35963
IFIH1	NM_022168.2	0.003	0.184728	6.062449	1.28074	0.201396	0.321446	-6.36191
TNFSF13	NM_003808.3	0	0.162787	6.691757	1.267778	0.205981	0.32668	-6.37829
SLAMF6	NM_001184714.1	0.267	0.330842	2.493996	1.266962	0.206272	0.32668	-6.37932
NOD1	NM_006092.1	0.082	-0.2416	3.62329	-1.26584	0.206675	0.32668	-6.38073
TNFRSF13B	NM_012452.2	0.418	-0.31274	1.686095	-1.23981	0.216135	0.340534	-6.41308
ATG5	NM_004849.2	0	-0.08647	6.209561	-1.22835	0.220397	0.346136	-6.42712
BLK	NM_001715.2	0.12	-0.28945	3.655608	-1.21111	0.226923	0.354869	-6.44798
IL21R	NM_021798.2	0.082	-0.25475	3.908427	-1.20842	0.227953	0.354869	-6.45121
TGFB1	NM_000660.3	0	0.096284	10.95069	1.207962	0.22813	0.354869	-6.45176
UBC	NM_021009.3	0	0.089996	14.08619	1.201947	0.230449	0.357341	-6.45896
TRAF6	NM_145803.1	0	-0.09946	6.432924	-1.1894	0.235339	0.363773	-6.47385
STAT2	NM_005419.2	0	0.144952	7.762665	1.179109	0.239404	0.368893	-6.48595
IL1RN	NM_000577.3	0.003	-0.23336	7.56642	-1.17574	0.240747	0.36978	-6.48989
SERPINB2	NM_002575.1	0.075	0.398617	5.633046	1.17388	0.241489	0.36978	-6.49206
IL8	NM_000584.2	0	0.238813	13.13576	1.161298	0.246558	0.374256	-6.50664
TAPBP	NM_003190.4	0	0.083916	9.857129	1.160946	0.2467	0.374256	-6.50705
PSMD7	NM_002811.3	0	-0.0742	8.443101	-1.16094	0.246704	0.374256	-6.50706
TIRAP	NM_148910.2	0.144	0.208686	2.624071	1.1524	0.250187	0.37837	-6.51687

MAP4K2	NM_004579.2	0	-0.07364	6.797415	-1.13127	0.258958	0.390428	-6.54083
BCL2L1	NM_001191.2	0	0.252998	8.230849	1.121969	0.262883	0.395131	-6.55124
IL6ST	NM_002184.2	0	0.089363	8.300979	1.117016	0.264991	0.395603	-6.55674
VEGFA	NM_001025366.1	0	0.133195	7.925177	1.116633	0.265155	0.395603	-6.55717
LYN	NM_002350.1	0	-0.10259	8.412352	-1.11555	0.265619	0.395603	-6.55837
EWSR1	NM_013986.3	0	0.048272	8.235811	1.111353	0.267416	0.396654	-6.56301
ICAM2	NM_000873.3	0	-0.11122	6.419583	-1.11012	0.267944	0.396654	-6.56437
DUSP4	NM_057158.2	0.164	0.307541	3.536306	1.106762	0.269392	0.397597	-6.56807
IRF2	NM_002199.3	0	0.125825	6.093233	1.103807	0.27067	0.398284	-6.57131
HLA-B	NM_005514.6	0	0.06257	13.57856	1.099096	0.272716	0.400092	-6.57647
HLA-DPA1	NM_033554.2	0	0.10823	10.29383	1.0922	0.275729	0.402578	-6.58398
CXCR2	NM_001557.2	0.058	-0.34404	6.028551	-1.09146	0.276054	0.402578	-6.58478
CCL20	NM_004591.1	0.411	0.304193	1.999293	1.08764	0.277734	0.403827	-6.58891
C1QB	NM_000491.3	0.195	0.313921	3.38991	1.085578	0.278644	0.403952	-6.59114
ITGA6	NM_000210.1	0.062	-0.22526	4.19563	-1.08271	0.279912	0.404593	-6.59423
CD19	NM_001770.4	0.055	-0.26062	4.816248	-1.07958	0.281304	0.405409	-6.59759
IL16	NM_004513.4	0	-0.06057	7.722864	-1.07575	0.283008	0.405746	-6.60169
IFNAR2	NM_000874.3	0	-0.0721	8.301537	-1.07534	0.283194	0.405746	-6.60213
IFI16	NM_005531.1	0	-0.09556	6.908296	-1.07132	0.284994	0.407135	-6.60642
DDX58	NM_014314.3	0.031	-0.21558	5.32417	-1.03466	0.301764	0.429839	-6.64479
ADA	NM_000022.2	0.007	0.125845	5.724951	1.021704	0.307845	0.43723	-6.65803
TP53	NM_000546.2	0.014	0.160834	5.277781	1.006197	0.315231	0.446426	-6.67366
CTSG	NM_001911.2	0.462	-0.27137	1.792806	-1.00393	0.316323	0.446681	-6.67593
ENG	NM_001114753.1	0.051	0.194946	4.763187	0.98663	0.324717	0.457216	-6.69304
IFNAR1	NM_000629.2	0.171	0.213625	2.674679	0.974803	0.33054	0.46402	-6.70458
TRAF2	NM_021138.3	0.048	0.168356	3.923246	0.972981	0.331443	0.46402	-6.70634
ICAM1	NM_000201.2	0	0.146275	7.861002	0.963163	0.336337	0.467682	-6.71579
FCER2	NM_002002.4	0.082	-0.25436	4.805759	-0.96106	0.337389	0.467682	-6.7178
THBS1	NM_003246.2	0	0.25518	10.2535	0.960582	0.337631	0.467682	-6.71826
TFE3	NM_006521.3	0.007	0.107958	4.911264	0.960093	0.337877	0.467682	-6.71873

CD28	NM_001243078.1	0.178	-0.23537	3.234351	-0.9537	0.341096	0.469709	-6.72481
HMGB1	NM_002128.4	0	0.052678	9.277236	0.953384	0.341258	0.469709	-6.72511
IRAK2	NM_001570.3	0.021	0.1818	4.751827	0.951481	0.34222	0.469714	-6.72692
IL13RA1	NM_001560.2	0	0.102063	7.233484	0.923108	0.356784	0.488104	-6.75337
TFRC	NM_003234.1	0	0.1063	7.850671	0.92152	0.357611	0.488104	-6.75483
HLA-G	NM_002127.4	0	0.06908	7.159786	0.913162	0.361981	0.491478	-6.76246
IL10	NM_000572.2	0.253	0.257615	2.957527	0.912957	0.362089	0.491478	-6.76264
C1QA	NM_015991.2	0.127	-0.21881	4.247896	-0.89677	0.370648	0.501707	-6.77721
CXCL16	NM_001100812.1	0.003	0.139032	7.241422	0.891576	0.373423	0.504069	-6.78184
SLAMF7	NM_021181.3	0	-0.10366	6.269028	-0.88419	0.377386	0.50737	-6.78836
IL15RA	NM_002189.2	0	0.085227	5.735257	0.883169	0.377939	0.50737	-6.78926
POU2F2	NM_002698.2	0.003	0.096551	6.756974	0.875423	0.38213	0.511595	-6.79604
CXCR5	NM_001716.3	0.171	-0.20507	3.232261	-0.87282	0.383545	0.512091	-6.7983
EP300	NM_001429.2	0	0.061543	8.468199	0.854861	0.393394	0.523813	-6.81374
TNFSF8	NM_001244.3	0.007	0.105252	5.546341	0.851631	0.395182	0.524767	-6.81648
C1QBP	NM_001212.3	0	-0.06452	7.336941	-0.83765	0.402978	0.533674	-6.82824
IL2RG	NM_000206.1	0	0.086111	8.597442	0.826763	0.409109	0.539772	-6.83726
HLA-E	NM_005516.4	0	0.05273	11.6039	0.825567	0.409786	0.539772	-6.83824
IRF1	NM_002198.1	0	0.10842	10.08568	0.811403	0.417857	0.547776	-6.84978
CCR3	NM_001837.2	0.455	0.184827	1.557105	0.810982	0.418098	0.547776	-6.85012
ADORA2A	NM_000675.3	0.003	0.15153	6.562508	0.807525	0.420083	0.548908	-6.85291
CLEC7A	NM_197954.2	0	0.113574	8.481846	0.805373	0.421321	0.549061	-6.85463
ST6GAL1	NM_003032.2	0.003	-0.10351	7.03997	-0.8028	0.422806	0.549536	-6.8567
BMI1	NM_005180.5	0	-0.10325	7.752925	-0.78136	0.435284	0.562806	-6.8736
RELB	NM_006509.2	0	-0.0971	6.86915	-0.78131	0.435313	0.562806	-6.87364
REL	NM_002908.2	0	-0.07364	7.632005	-0.77686	0.437931	0.5647	-6.8771
CDKN1A	NM_000389.2	0	0.081272	9.091459	0.773701	0.439792	0.565611	-6.87953
GZMM	NM_005317.2	0.01	-0.10206	5.841592	-0.76406	0.445508	0.571463	-6.88692
DOCK9	NM_001130048.1	0.024	-0.11841	4.756119	-0.75865	0.448729	0.574092	-6.89101
LY9	NM_001033667.1	0	-0.07538	6.883577	-0.74605	0.456292	0.580377	-6.90045

IGF1R	NM_000875.2	0	-0.11133	6.296926	-0.74591	0.456377	0.580377	-6.90056
RORA	NM_134261.2	0.003	-0.10543	6.797615	-0.74456	0.457195	0.580377	-6.90157
MICB	NM_005931.3	0	-0.05404	6.878711	-0.74203	0.458719	0.580808	-6.90343
CX3CR1	NM_001337.3	0.021	0.185298	5.610803	0.72505	0.469056	0.589108	-6.91584
PNMA1	NM_006029.4	0	0.074089	5.465363	0.721493	0.471238	0.589108	-6.9184
HLA-A	NM_002116.5	0	0.043542	13.45711	0.721279	0.471369	0.589108	-6.91856
KLRK1	NM_007360.3	0.007	0.099749	8.067134	0.721143	0.471452	0.589108	-6.91865
IL23A	NM_016584.2	0.291	-0.16181	1.983131	-0.71953	0.472446	0.589108	-6.91981
TCF7	NM_003202.2	0.021	0.132013	7.021768	0.719457	0.472488	0.589108	-6.91986
MSR1	NM_002445.3	0.082	0.182315	4.379792	0.704504	0.481733	0.599109	-6.93047
FOXJ1	NM_001454.3	0	-0.08887	5.448176	-0.68943	0.491154	0.609279	-6.94094
HAVCR2	NM_032782.3	0.014	0.09501	5.285496	0.661639	0.508773	0.629542	-6.95964
NFATC2	NM_012340.3	0	-0.06476	7.254581	-0.65178	0.515106	0.635773	-6.96609
CD37	NM_001774.2	0.003	-0.05473	7.4318	-0.64921	0.516759	0.636176	-6.96775
S100B	NM_006272.1	0.5	0.169354	1.716099	0.647246	0.518029	0.636176	-6.96902
TREM1	NM_018643.3	0	0.104434	9.971133	0.639519	0.523033	0.640716	-6.97398
IL18RAP	NM_003853.2	0.065	-0.17737	4.95418	-0.63668	0.524878	0.641067	-6.97578
CYFIP2	NM_001037332.2	0	0.051621	7.800324	0.635054	0.525937	0.641067	-6.97681
CD40LG	NM_000074.2	0.082	0.131066	3.887451	0.610916	0.541774	0.658296	-6.9918
TAP1	NM_000593.5	0	0.057792	7.406064	0.607463	0.544059	0.658296	-6.9939
NFATC3	NM_004555.2	0	-0.04096	6.85298	-0.60452	0.546011	0.658296	-6.99567
FLT3LG	NM_001459.3	0	0.055733	7.150165	0.603496	0.546691	0.658296	-6.99629
CXCL5	NM_002994.3	0.027	0.135741	5.612661	0.603349	0.546788	0.658296	-6.99638
IL3RA	NM_002183.2	0.161	0.13334	2.90557	0.595084	0.552291	0.663291	-7.00131
HLA-C	NM_002117.4	0	0.040013	12.6451	0.590276	0.555505	0.665519	-7.00415
PSEN1	NM_000021.2	0	0.038059	6.638653	0.579943	0.562442	0.672187	-7.01018
TNFAIP3	NM_006290.2	0	0.078881	11.40526	0.577028	0.564406	0.672893	-7.01186
CREB1	NM_004379.3	0	-0.04851	5.91797	-0.53694	0.591756	0.703787	-7.03412
ATF1	NM_005171.2	0.007	-0.05594	5.483778	-0.529	0.597247	0.707297	-7.03834
IRAK4	NM_016123.1	0.007	0.058234	5.477751	0.528496	0.597594	0.707297	-7.0386

IRF8	NM_002163.2	0	-0.04601	7.381763	-0.51217	0.608956	0.718601	-7.04707
TLR10	NM_030956.2	0.096	0.10918	3.325336	0.509452	0.610856	0.718601	-7.04846
ATG16L1	NM_198890.2	0.007	0.056687	5.877359	0.506868	0.612666	0.718601	-7.04977
IL11RA	NM_147162.1	0.161	0.11266	3.040718	0.506376	0.61301	0.718601	-7.05002
IL7R	NM_002185.2	0.01	0.078198	7.407723	0.483232	0.629327	0.735967	-7.06143
FCER1A	NM_002001.2	0.12	0.139981	4.494639	0.472221	0.637155	0.743347	-7.06668
BID	NM_001196.2	0.038	0.056804	5.419228	0.465418	0.642011	0.747234	-7.06986
CD96	NM_005816.4	0.007	0.061617	6.991743	0.459163	0.646491	0.747959	-7.07274
HLA-DQB1	NM_002123.3	0.315	-0.21103	5.682745	-0.45823	0.647163	0.747959	-7.07317
PSMB7	NM_002799.2	0	-0.02535	8.642375	-0.45816	0.647213	0.747959	-7.0732
CD22	NM_001771.2	0.068	-0.12103	5.534534	-0.45153	0.651977	0.751691	-7.07621
PLA2G6	NM_001004426.1	0.034	0.070664	4.049692	0.439319	0.660786	0.759036	-7.08163
LTA	NM_000595.2	0.017	-0.08495	5.180914	-0.43841	0.661446	0.759036	-7.08203
ILF3	NM_001137673.1	0	-0.02553	8.574618	-0.4227	0.672855	0.770325	-7.08878
OSM	NM_020530.4	0.014	0.079023	6.986592	0.417738	0.676474	0.772663	-7.09086
ZAP70	NM_001079.3	0.003	-0.05358	7.294081	-0.39317	0.69451	0.791419	-7.10079
FOS	NM_005252.2	0	-0.03882	12.90851	-0.37914	0.704883	0.801375	-7.10619
IRF5	NM_002200.3	0	0.033421	6.507566	0.364433	0.715823	0.811929	-7.11165
CD1C	NM_001765.2	0.113	0.094423	4.009041	0.359838	0.719252	0.81347	-7.11331
LCN2	NM_005564.3	0.007	-0.0795	6.196887	-0.35817	0.720502	0.81347	-7.11391
ITGAE	NM_002208.4	0	-0.03505	6.747701	-0.35362	0.723902	0.815429	-7.11552
IL1RAP	NM_002182.2	0	-0.05549	6.01225	-0.33445	0.738306	0.828346	-7.1221
YTHDF2	NM_001172828.1	0.01	0.038528	5.984099	0.332977	0.739413	0.828346	-7.12259
CD48	NM_001778.2	0	-0.02725	10.49278	-0.33162	0.74044	0.828346	-7.12304
IL1B	NM_000576.2	0.031	0.096229	6.876044	0.325897	0.744757	0.831278	-7.12492
CFD	NM_001928.2	0	-0.04064	6.853647	-0.31312	0.754431	0.840085	-7.12899
CXCR1	NM_000634.2	0.38	0.08596	2.291848	0.310956	0.756076	0.840085	-7.12967
ITGA4	NM_000885.4	0	-0.02192	7.624805	-0.29045	0.771695	0.855499	-7.13582
LY86	NM_004271.3	0	0.0302	7.733498	0.285574	0.775426	0.857695	-7.13722
NFKBIA	NM_020529.1	0	-0.03704	13.49199	-0.27474	0.783728	0.864925	-7.14025

MAPKAPK2	NM_004759.3	0	-0.0265	8.333636	-0.25475	0.799114	0.878633	-7.14553
ATF2	NM_001256090.1	0	-0.01359	6.355255	-0.25394	0.799736	0.878633	-7.14573
LY96	NM_015364.2	0	0.032096	7.682891	0.241835	0.809094	0.886926	-7.14873
HLA-DQA1	NM_002122.3	0.325	-0.11175	6.135714	-0.22641	0.821057	0.898032	-7.15233
ITK	NM_005546.3	0.007	-0.02951	7.219445	-0.22109	0.825194	0.899854	-7.15352
MS4A1	NM_152866.2	0.051	-0.05722	5.816769	-0.21954	0.826396	0.899854	-7.15386
CD164	NM_006016.4	0	-0.01464	8.347077	-0.21378	0.830881	0.902731	-7.1551
IFNGR1	NM_000416.1	0	-0.02342	8.654605	-0.20884	0.834729	0.904906	-7.15614
TNFSF10	NM_003810.2	0	-0.03057	7.480651	-0.20519	0.837584	0.905996	-7.1569
IFITM2	NM_006435.2	0	0.023987	10.97691	0.195966	0.844786	0.911774	-7.15875
EGR2	NM_000399.3	0.164	-0.06166	3.880796	-0.19315	0.846987	0.91214	-7.15929
TNFRSF10B	NM_003842.3	0.014	-0.02073	5.712225	-0.18922	0.850062	0.913444	-7.16004
MICA	NM_000247.1	0.178	-0.03878	2.747806	-0.18356	0.854501	0.916204	-7.1611
NT5E	NM_002526.2	0.137	-0.03921	2.742171	-0.17679	0.859808	0.919882	-7.16231
SERPING1	NM_000062.2	0.024	0.038353	5.201847	0.162896	0.870723	0.928173	-7.16467
TNFRSF1B	NM_001066.2	0	0.013391	9.797379	0.159926	0.87306	0.928173	-7.16515
TFEB	NM_007162.2	0.014	0.020696	5.444916	0.159697	0.87324	0.928173	-7.16518
CCND3	NM_001760.2	0	0.013033	8.801348	0.154851	0.877056	0.930211	-7.16594
IL32	NM_001012633.1	0	-0.01784	10.97232	-0.14732	0.882987	0.931925	-7.16708
RELA	NM_021975.2	0	0.01208	6.820764	0.146826	0.88338	0.931925	-7.16715
SH2B2	NM_020979.3	0	-0.01703	6.215647	-0.14556	0.884378	0.931925	-7.16733
TBK1	NM_013254.2	0	0.012682	6.272289	0.140713	0.888202	0.933947	-7.16803
PDGFC	NM_016205.2	0.147	-0.02704	3.027464	-0.12105	0.903746	0.948256	-7.1706
ELK1	NM_005229.3	0.031	-0.01725	4.493141	-0.11209	0.910835	0.950519	-7.17164
IL15	NM_172174.1	0.024	-0.01641	4.529072	-0.11187	0.911011	0.950519	-7.17166
IRAK1	NM_001569.3	0	0.007457	7.226552	0.106911	0.91494	0.950519	-7.1722
MAPK8	NM_002750.2	0.017	0.013963	4.508	0.106636	0.915158	0.950519	-7.17223
HLA-DRB4	NM_021983.4	0.586	-0.05579	3.564225	-0.10404	0.917216	0.950519	-7.17251
NFKB1	NM_003998.2	0.003	-0.01186	5.891648	-0.10363	0.917542	0.950519	-7.17255
ALCAM	NM_001627.3	0.048	0.014925	3.985995	0.08761	0.930252	0.961653	-7.17408

CASP8	NM_001228.4	0	-0.00908	8.914187	-0.08264	0.934202	0.963703	-7.1745
MAP3K7	NM_145333.1	0	0.004387	6.651272	0.074807	0.940424	0.968084	-7.17511
TANK	NM_004180.2	0	-0.00596	8.704286	-0.07053	0.943827	0.96955	-7.17543
MEF2C	NM_002397.3	0.01	0.012584	6.102815	0.067942	0.945882	0.969628	-7.1756
IFI35	NM_005533.3	0.027	-0.01384	5.329995	-0.06431	0.948771	0.970559	-7.17584
MR1	NM_001531.2	0.062	0.008867	3.313179	0.05128	0.959141	0.979123	-7.1766
ITCH	NM_001257138.1	0	0.002758	8.069219	0.041977	0.966548	0.984634	-7.17703
EGR1	NM_001964.2	0.031	0.010525	6.703873	0.03938	0.968617	0.984693	-7.17713
SPN	NM_003123.3	0.017	-0.00554	5.328756	-0.03139	0.974985	0.989115	-7.17742
TNF	NM_000594.2	0	0.003392	7.861256	0.025027	0.980052	0.992202	-7.17759
TLR7	NM_016562.3	0.473	-0.00364	1.375682	-0.01651	0.986838	0.994998	-7.17777
MAP3K5	NM_005923.3	0	0.001323	7.51705	0.016466	0.986875	0.994998	-7.17777
TRAF3	NM_145725.1	0	-0.00075	7.01356	-0.01025	0.99183	0.99794	-7.17785
IL4R	NM_000418.2	0	-0.00035	7.572045	-0.00357	0.997152	0.999437	-7.1779
IFIT2	NM_001547.4	0.003	-0.00071	6.889349	-0.00326	0.997398	0.999437	-7.1779
CCR6	NM_031409.2	0.301	0.000153	2.05276	0.000706	0.999437	0.999437	-7.17791
**B. Rd at C3D1 versus C1D1**								
SLC11A1	NM_000578.2	0	0.649853	8.417996	5.398942	1.48E-07	7.26E-05	7.102108
PVR	NM_006505.3	0.182	1.110499	2.843678	4.911774	1.57E-06	0.000306	4.895668
CD163	NM_004244.4	0.007	1.006155	7.064932	4.84504	2.15E-06	0.000306	4.606771
AKT3	NM_181690.1	0.003	-0.58549	6.132502	-4.81202	2.50E-06	0.000306	4.465031
PTPRC	NM_080921.3	0	0.556757	9.119697	4.676755	4.63E-06	0.000454	3.892933
LTBR	NM_002342.1	0.007	0.54105	6.519281	4.42419	1.41E-05	0.001152	2.861749
FCGR1A	NM_000566.3	0.202	0.966237	2.38127	4.318082	2.22E-05	0.00144	2.443168
CLEC4C	NM_203503.1	0.455	-1.03891	1.61876	-4.29878	2.41E-05	0.00144	2.367971
C1QA	NM_015991.2	0.127	1.089717	4.247896	4.276552	2.65E-05	0.00144	2.28173
S100A12	NM_005621.1	0	0.592689	10.46119	4.074347	6.09E-05	0.002983	1.515151
TLR5	NM_003268.3	0.202	1.044633	3.00475	3.882995	0.00013	0.005796	0.819838
CD19	NM_001770.4	0.055	-0.96608	4.816248	-3.83208	0.000158	0.006471	0.639815
PSMB10	NM_002801.2	0	0.260148	8.926977	3.760922	0.000208	0.006944	0.39181

C1QB	NM_000491.3	0.195	1.135341	3.38991	3.759569	0.000209	0.006944	0.387136
FCER1G	NM_004106.1	0	0.372388	10.93316	3.755241	0.000213	0.006944	0.372192
CD33	NM_001177608.1	0.024	0.637066	5.324418	3.735947	0.000229	0.007003	0.305749
TNFRSF13C	NM_052945.3	0.045	-0.95699	6.038428	-3.68252	0.000279	0.008055	0.123366
REPS1	NM_001128617.2	0	-0.3198	7.134522	-3.65881	0.000305	0.00831	0.043177
CYBB	NM_000397.3	0	0.433378	9.699284	3.605692	0.000371	0.009578	-0.13474
CD1D	NM_001766.3	0	0.422858	6.7726	3.587927	0.000396	0.009711	-0.19372
BTLA	NM_181780.2	0.034	-0.74037	4.895995	-3.52687	0.000495	0.01121	-0.39444
MARCO	NM_006770.3	0.031	0.76794	5.582365	3.522134	0.000503	0.01121	-0.40987
PAX5	NM_016734.1	0.178	-1.02839	3.896755	-3.50826	0.000529	0.011271	-0.45499
NLRP3	NM_001079821.2	0	0.462995	7.913347	3.323034	0.001015	0.02072	-1.04173
CD79A	NM_001783.3	0.003	-0.69935	7.378975	-3.27274	0.001205	0.023473	-1.19601
YTHDF2	NM_001172828.1	0.01	-0.39429	5.984099	-3.26309	0.001246	0.023473	-1.22537
IL17RA	NM_014339.6	0.003	0.392085	7.084215	3.194116	0.001571	0.028507	-1.43286
TLR4	NM_138554.2	0.003	0.457586	6.63778	3.165404	0.001728	0.030241	-1.51803
MAP2K1	NM_002755.2	0	0.278369	7.536886	3.072457	0.002342	0.03958	-1.78884
CD79B	NM_021602.2	0	-0.52153	7.068639	-3.05715	0.002461	0.0402	-1.83273
TNFRSF9	NM_001561.4	0.329	0.675476	1.935197	3.012679	0.002838	0.040448	-1.95905
CD22	NM_001771.2	0.068	-0.84185	5.534534	-3.00746	0.002886	0.040448	-1.97376
MEFV	NM_000243.2	0.003	0.42648	7.319955	3.007048	0.002889	0.040448	-1.97492
CD14	NM_000591.2	0	0.455382	9.392181	3.005605	0.002903	0.040448	-1.97898
HCK	NM_002110.2	0	0.365201	9.069937	3.002509	0.002931	0.040448	-1.98769
ST6GAL1	NM_003032.2	0.003	-0.40176	7.03997	-2.98377	0.003111	0.040448	-2.04023
CTSS	NM_004079.3	0	0.289475	12.66577	2.979909	0.003149	0.040448	-2.05103
CFP	NM_002621.2	0	0.286048	8.896465	2.979278	0.003155	0.040448	-2.05279
STAT1	NM_007315.2	0	0.350761	8.891621	2.972893	0.003219	0.040448	-2.0706
ENTPD1	NM_001098175.1	0.092	0.630554	3.750198	2.914531	0.003864	0.043183	-2.23173
LRP1	NM_002332.2	0	0.311489	7.60475	2.908183	0.003941	0.043183	-2.24908
ITGAM	NM_000632.3	0	0.289988	8.143104	2.90555	0.003973	0.043183	-2.25626
IL18RAP	NM_003853.2	0.065	0.844331	4.95418	2.902213	0.004014	0.043183	-2.26536

ITGB2	NM_000211.2	0	0.226878	10.81662	2.889894	0.00417	0.043183	-2.29886
IL13RA1	NM_001560.2	0	0.333556	7.233484	2.888847	0.004184	0.043183	-2.3017
TLR8	NM_016610.2	0.014	0.429362	5.396971	2.887871	0.004196	0.043183	-2.30435
IRF8	NM_002163.2	0	0.270587	7.381763	2.884113	0.004245	0.043183	-2.31453
IL6R	NM_000565.2	0	0.273818	6.492918	2.875677	0.004357	0.043183	-2.33735
FPR2	NM_001462.3	0.041	0.665984	5.147605	2.875106	0.004364	0.043183	-2.33889
RPS6	NM_001010.2	0	-0.25575	12.82758	-2.87196	0.004406	0.043183	-2.34737
NOD2	NM_022162.1	0	0.317584	6.60489	2.845446	0.004779	0.045911	-2.4186
TLR2	NM_003264.3	0	0.382829	7.639966	2.821326	0.005142	0.04788	-2.48285
IL18	NM_001562.2	0.024	0.457216	5.163597	2.812994	0.005273	0.04788	-2.50493
PYCARD	NM_013258.3	0	0.284235	7.013016	2.807569	0.00536	0.04788	-2.51927
TOLLIP	NM_019009.2	0.01	0.323312	5.198327	2.802061	0.005449	0.04788	-2.5338
NFATC1	NM_172389.1	0.007	-0.31559	5.23277	-2.80069	0.005472	0.04788	-2.53742
MS4A1	NM_152866.2	0.051	-0.75848	5.816769	-2.78658	0.005709	0.049076	-2.57452
CARD9	NM_052813.4	0.089	0.622355	4.2204	2.765526	0.006079	0.05136	-2.62952
RIPK2	NM_003821.5	0	0.347649	8.656458	2.759609	0.006187	0.051387	-2.64491
IL10	NM_000572.2	0.253	0.809981	2.957527	2.748686	0.006391	0.052197	-2.67324
IL18R1	NM_003855.2	0.106	0.727393	3.790822	2.730764	0.006739	0.054136	-2.7195
ANXA1	NM_000700.1	0	0.266762	11.06019	2.723883	0.006877	0.054354	-2.73718
ITGA5	NM_002205.2	0	0.21663	7.295539	2.700133	0.007374	0.057352	-2.79789
HAVCR2	NM_032782.3	0.014	0.399003	5.285496	2.660709	0.00827	0.062624	-2.89755
CASP1	NM_001223.3	0	0.281518	8.02859	2.65724	0.008353	0.062624	-2.90626
FCER2	NM_002002.4	0.082	-0.73308	4.805759	-2.65235	0.008472	0.062624	-2.91852
BLK	NM_001715.2	0.12	-0.66107	3.655608	-2.64863	0.008563	0.062624	-2.92781
HLA-DMB	NM_002118.3	0	0.275923	8.280356	2.635523	0.008892	0.064074	-2.9605
AMICA1	NM_153206.2	0	0.369879	8.896182	2.596844	0.00993	0.070516	-3.05606
CD68	NM_001251.2	0	0.241568	9.788476	2.585415	0.010257	0.071796	-3.08404
JAK2	NM_004972.2	0	0.239842	7.932236	2.578903	0.010447	0.072099	-3.09993
MIF	NM_002415.1	0	-0.20136	8.121836	-2.56869	0.010752	0.073175	-3.12478
KLRC2	NM_002260.3	0.134	0.632118	3.811944	2.56281	0.010931	0.073375	-3.13903

HLA-DOB	NM_002120.3	0.01	-0.48195	5.225432	-2.54299	0.011555	0.076515	-3.18687
HLA-DMA	NM_006120.3	0	0.218716	8.650773	2.500209	0.013012	0.085011	-3.28894
CD36	NM_001001548.2	0	0.348625	9.040517	2.493555	0.013253	0.085444	-3.30466
LILRA1	NM_006863.1	0.038	0.518531	5.249274	2.474952	0.013946	0.087884	-3.34842
IL10RA	NM_001558.2	0	0.170624	9.67183	2.473823	0.01399	0.087884	-3.35107
CCRL2	NM_003965.4	0.072	0.493346	3.840413	2.464305	0.014358	0.089056	-3.37332
MERTK	NM_006343.2	0.171	0.571311	2.826829	2.454263	0.014756	0.089781	-3.39671
TNFSF13	NM_003808.3	0	0.328814	6.691757	2.452137	0.014841	0.089781	-3.40165
APP	NM_000484.3	0	0.216949	7.901192	2.441838	0.015262	0.090407	-3.42553
BST1	NM_004334.2	0	0.279936	8.429325	2.431263	0.015704	0.090407	-3.44995
LY9	NM_001033667.1	0	-0.25652	6.883577	-2.43112	0.01571	0.090407	-3.45027
LILRB2	NM_005874.1	0	0.244214	7.646844	2.427443	0.015867	0.090407	-3.45874
TNFRSF1A	NM_001065.2	0	0.236777	7.604868	2.427435	0.015867	0.090407	-3.45876
MX1	NM_002462.2	0	-0.38316	7.738464	-2.42307	0.016055	0.090426	-3.4688
MAPK14	NM_001315.1	0	0.323065	8.18495	2.411446	0.016564	0.092234	-3.49543
THBS1	NM_003246.2	0	0.662285	10.2535	2.387285	0.017669	0.09728	-3.5504
ATG5	NM_004849.2	0	-0.1742	6.209561	-2.36956	0.01852	0.100085	-3.59039
BCL6	NM_001706.2	0	0.320457	9.486685	2.368199	0.018587	0.100085	-3.59345
FYN	NM_002037.3	0	0.19631	8.537363	2.357312	0.019129	0.101885	-3.61786
C2	NM_000063.3	0.243	0.607295	2.578399	2.351354	0.019432	0.102384	-3.63117
CCR2	NM_001123041.2	0.175	0.70728	3.44741	2.31932	0.021132	0.110157	-3.70219
S100A8	NM_002964.3	0	0.407041	12.77144	2.304992	0.021934	0.112043	-3.73366
POU2AF1	NM_006235.2	0.455	-0.64524	1.816663	-2.30469	0.021951	0.112043	-3.73432
CD47	NM_001777.3	0	-0.11342	8.84671	-2.2628	0.024451	0.123518	-3.82523
IFITM1	NM_003641.3	0	-0.24982	10.38768	-2.23693	0.026116	0.130581	-3.88055
CD200	NM_005944.5	0.226	-0.55472	2.696199	-2.22323	0.027037	0.133213	-3.90961
TLR10	NM_030956.2	0.096	-0.49708	3.325336	-2.22105	0.027186	0.133213	-3.91422
CD24	NM_013230.2	0.007	-0.4546	5.745172	-2.21158	0.027843	0.133577	-3.9342
S100B	NM_006272.1	0.5	0.60387	1.716099	2.20998	0.027955	0.133577	-3.93755
CEBPB	NM_005194.2	0	0.25002	9.52179	2.208221	0.028078	0.133577	-3.94125

ISG15	NM_005101.3	0	-0.38291	7.013594	-2.1724	0.030705	0.144668	-4.01592
ECSIT	NM_001142464.2	0.003	0.255897	5.140958	2.163798	0.031366	0.146377	-4.03368
ARG1	NM_000045.2	0.408	0.709211	2.243983	2.153398	0.032183	0.148768	-4.05505
IKBKB	NM_001556.1	0.024	0.369268	4.661722	2.129514	0.034126	0.155876	-4.10377
NLRC5	NM_032206.4	0.003	0.251502	7.113546	2.126765	0.034356	0.155876	-4.10934
LCP1	NM_002298.4	0	0.170876	11.29735	2.114978	0.035358	0.15849	-4.13316
CD4	NM_000616.4	0	0.228019	7.792406	2.11241	0.035579	0.15849	-4.13834
IGF2R	NM_000876.1	0.003	0.258861	6.933908	2.105662	0.036167	0.158934	-4.1519
EWSR1	NM_013986.3	0	0.095431	8.235811	2.103836	0.036328	0.158934	-4.15556
IFIT1	NM_001548.3	0.079	-0.60582	4.665226	-2.09225	0.03736	0.162005	-4.17873
JAK3	NM_000215.2	0	0.187426	7.79399	2.060498	0.040319	0.170947	-4.24159
TXK	NM_003328.1	0.202	-0.46705	2.831012	-2.06026	0.040342	0.170947	-4.24206
SERPING1	NM_000062.2	0.024	0.506245	5.201847	2.058943	0.040469	0.170947	-4.24465
TYK2	NM_003331.3	0	0.166719	6.65378	2.053387	0.041009	0.171746	-4.25554
IL11RA	NM_147162.1	0.161	0.474122	3.040718	2.040632	0.042271	0.174776	-4.28045
PLAUR	NM_001005376.1	0	0.2726	9.382228	2.038888	0.042446	0.174776	-4.28384
MAPK1	NM_138957.2	0	0.155235	8.063744	2.024576	0.043906	0.179282	-4.31159
KIR_Activating_Subgroup_2	NM_014512.1	0.349	0.542072	2.233523	2.006404	0.045821	0.185556	-4.34654
ITGAX	NM_000887.3	0	0.189696	9.114923	1.99116	0.047482	0.190706	-4.37563
IFNGR1	NM_000416.1	0	0.231399	8.654605	1.976196	0.049161	0.194662	-4.40397
IL3RA	NM_002183.2	0.161	0.460785	2.90557	1.969183	0.049966	0.194662	-4.41718
LTA	NM_000595.2	0.017	-0.39844	5.180914	-1.96893	0.049995	0.194662	-4.41766
PNMA1	NM_006029.4	0	-0.21109	5.465363	-1.9684	0.050056	0.194662	-4.41866
STAT2	NM_005419.2	0	0.25222	7.762665	1.964631	0.050493	0.194817	-4.42574
IRF5	NM_002200.3	0	0.187137	6.507566	1.954044	0.05174	0.196714	-4.44555
IL1R2	NM_173343.1	0.027	0.678679	5.976301	1.948863	0.052359	0.196714	-4.45521
CTSH	NM_004390.3	0	0.197465	8.795855	1.947248	0.052553	0.196714	-4.45821
GZMK	NM_002104.2	0.01	0.388655	6.858668	1.946933	0.052591	0.196714	-4.4588
DUSP6	NM_001946.2	0	0.287268	8.345518	1.942615	0.053114	0.197164	-4.46682
ITGB1	NM_033666.2	0	0.151899	9.274698	1.9335	0.054231	0.199799	-4.4837

CCR4	NM_005508.4	0.062	0.40484	4.225344	1.92629	0.055129	0.201592	-4.497
IRF1	NM_002198.1	0	0.268003	10.08568	1.920608	0.055846	0.201963	-4.50744
CD40	NM_001250.4	0.021	0.348471	5.137436	1.918643	0.056095	0.201963	-4.51105
STAT6	NM_003153.3	0	0.191287	9.220851	1.913643	0.056735	0.201963	-4.52021
SYK	NM_003177.3	0	0.203681	7.845749	1.911712	0.056983	0.201963	-4.52374
CR1	NM_000651.4	0	0.31129	8.079674	1.909324	0.057292	0.201963	-4.5281
FCGR2A	NM_021642.3	0	0.251237	9.886013	1.870898	0.062452	0.217856	-4.59754
CXCL16	NM_001100812.1	0.003	0.304399	7.241422	1.869201	0.062689	0.217856	-4.60058
CD63	NM_001780.4	0	0.201039	9.740343	1.861781	0.063732	0.219921	-4.61382
CREB5	NM_182898.2	0.007	0.342881	6.394364	1.827054	0.068807	0.235774	-4.6751
CD27	NM_001242.4	0.031	0.366202	5.303214	1.82131	0.069678	0.235942	-4.68513
IL15	NM_172174.1	0.024	0.278795	4.529072	1.820384	0.06982	0.235942	-4.68675
MFGE8	NM_001114614.1	0.055	0.367137	3.704272	1.812776	0.070989	0.237231	-4.69998
CLEC4A	NM_194448.2	0	0.180087	7.873964	1.808366	0.071674	0.237231	-4.70762
STAT3	NM_139276.2	0	0.138286	8.852237	1.805768	0.07208	0.237231	-4.71211
THBD	NM_000361.2	0.003	0.396772	8.640481	1.805403	0.072138	0.237231	-4.71274
BMI1	NM_005180.5	0	-0.24835	7.752925	-1.79966	0.073043	0.237993	-4.72266
CAMP	NM_004345.3	0.253	-0.54601	2.845462	-1.79778	0.073341	0.237993	-4.72589
LGALS3	NM_001177388.1	0	0.231598	9.566068	1.790041	0.07458	0.240422	-4.73918
OAS3	NM_006187.2	0.024	-0.4302	5.979037	-1.78585	0.075258	0.241022	-4.74636
CYFIP2	NM_001037332.2	0	-0.1507	7.800324	-1.77532	0.076984	0.244949	-4.76432
CD53	NM_001040033.1	0	0.130297	10.1139	1.75428	0.080529	0.254576	-4.79988
PSEN1	NM_000021.2	0	0.119523	6.638653	1.744023	0.082305	0.258523	-4.81707
IKBKG	NM_003639.2	0.003	0.171899	6.347981	1.737537	0.083445	0.260434	-4.82789
SBNO2	NM_014963.2	0.003	0.242296	6.477355	1.725033	0.085678	0.265055	-4.84863
CCL20	NM_004591.1	0.411	0.503224	1.999293	1.722927	0.086059	0.265055	-4.85211
IKBKE	NM_014002.2	0.027	0.348245	5.211535	1.720232	0.086548	0.265055	-4.85656
FEZ1	NM_005103.4	0.414	-0.39471	1.635389	-1.71457	0.087585	0.265057	-4.86589
MYD88	NM_002468.3	0	0.157277	8.563028	1.714314	0.087631	0.265057	-4.8663
IL15RA	NM_002189.2	0	0.171828	5.735257	1.705019	0.089354	0.26806	-4.88153

HLA-DRB3	NM_022555.3	0	0.162623	11.31087	1.701806	0.089955	0.26806	-4.88678
ATG7	NM_001136031.2	0.014	0.312047	5.433937	1.697908	0.09069	0.26806	-4.89313
ABL1	NM_005157.3	0.014	0.207507	4.733803	1.697261	0.090812	0.26806	-4.89418
BLNK	NM_013314.2	0.432	-0.42253	1.715381	-1.68986	0.092222	0.270592	-4.90621
LAIR2	NM_002288.3	0.014	0.283682	4.845662	1.662802	0.097525	0.284448	-4.9497
CTSG	NM_001911.2	0.462	0.466499	1.792806	1.652566	0.099594	0.287402	-4.96597
SMAD3	NM_005902.3	0	-0.19844	6.376146	-1.65199	0.099711	0.287402	-4.96688
SIGLEC1	NM_023068.3	0.182	-0.57625	3.955035	-1.63612	0.102992	0.293524	-4.99192
FCGR2B	NM_001002273.1	0.048	-0.36223	5.088996	-1.63592	0.103033	0.293524	-4.99222
TRAF6	NM_145803.1	0	-0.14214	6.432924	-1.6277	0.104767	0.294891	-5.0051
IL6ST	NM_002184.2	0	0.13593	8.300979	1.626997	0.104917	0.294891	-5.0062
ZAP70	NM_001079.3	0.003	0.231296	7.294081	1.625111	0.105318	0.294891	-5.00914
TAL1	NM_003189.2	0.058	0.391792	4.306962	1.619658	0.106486	0.295847	-5.01763
PDGFRB	NM_002609.3	0.349	-0.36159	1.901005	-1.6156	0.107362	0.295847	-5.02393
SPN	NM_003123.3	0.017	0.297969	5.328756	1.615094	0.107471	0.295847	-5.02472
PSMB9	NM_002800.4	0	0.191226	8.658391	1.609071	0.108782	0.297783	-5.03403
GZMA	NM_006144.2	0.01	0.33559	8.236576	1.602637	0.110197	0.29998	-5.04395
FLT3LG	NM_001459.3	0	-0.15312	7.150165	-1.58771	0.113534	0.307357	-5.0668
TRAF2	NM_021138.3	0.048	-0.28625	3.923246	-1.58413	0.114347	0.307857	-5.07226
IFI16	NM_005531.1	0	0.146863	6.908296	1.576568	0.116078	0.310811	-5.08373
CD59	NM_000611.4	0	-0.24161	6.047519	-1.56923	0.117779	0.313093	-5.09482
NFATC3	NM_004555.2	0	-0.1109	6.85298	-1.56739	0.118209	0.313093	-5.09759
IRF4	NM_002460.1	0.003	-0.24488	6.314729	-1.55799	0.120419	0.317233	-5.11169
CXCR5	NM_001716.3	0.171	-0.38	3.232261	-1.54869	0.122641	0.321303	-5.12557
CXCL2	NM_002089.3	0.058	0.503904	6.138127	1.546054	0.123276	0.321303	-5.12949
LILRA5	NM_181879.2	0	0.18787	9.279045	1.532967	0.126467	0.327879	-5.14884
CXCL1	NM_001511.1	0.144	0.479658	4.529862	1.528123	0.127665	0.329242	-5.15596
BCL2	NM_000657.2	0	-0.16938	5.932649	-1.51955	0.129807	0.333014	-5.16852
ITGA2B	NM_000419.3	0	0.277841	7.925738	1.508151	0.132698	0.336045	-5.18509
INPP5D	NM_005541.3	0	0.098115	7.592274	1.507026	0.132986	0.336045	-5.18672

KIR_Inhibiting_Subgroup_2	NM_014511.3	0.466	0.366432	1.691103	1.504105	0.133736	0.336045	-5.19095
RUNX1	NM_001754.4	0	0.135726	6.612867	1.502697	0.134099	0.336045	-5.19298
TICAM2	NM_021649.4	0.158	0.354664	3.007295	1.501459	0.134418	0.336045	-5.19477
CSF2RB	NM_000395.2	0	0.227045	8.605077	1.496065	0.135817	0.337819	-5.20254
CD44	NM_001001392.1	0	0.115002	11.27305	1.489173	0.137622	0.340579	-5.21243
MAPK3	NM_001040056.1	0	0.135772	6.641203	1.484638	0.138819	0.341815	-5.21891
STAT5B	NM_012448.3	0	0.1449	6.965891	1.481073	0.139766	0.342426	-5.22399
DOCK9	NM_001130048.1	0.024	-0.24066	4.756119	-1.47651	0.140985	0.343694	-5.23048
TFEB	NM_007162.2	0.014	0.198568	5.444916	1.467191	0.1435	0.348095	-5.24366
C3AR1	NM_004054.2	0.147	0.404109	3.763189	1.457435	0.146171	0.352827	-5.25738
TAP2	NM_000544.3	0	0.126885	7.793236	1.451425	0.147835	0.355094	-5.26578
TNFSF13B	NM_006573.4	0.003	0.228729	6.505515	1.44661	0.149179	0.356573	-5.27249
ADORA2A	NM_000675.3	0.003	-0.28249	6.562508	-1.44157	0.150595	0.358212	-5.27949
CHUK	NM_001278.3	0	0.142044	6.720205	1.433005	0.153026	0.362235	-5.29133
LILRB3	NM_006864.2	0	0.227995	7.73877	1.421607	0.156307	0.366304	-5.30698
CD244	NM_016382.2	0.017	0.227002	6.020149	1.421503	0.156337	0.366304	-5.30712
TLR6	NM_006068.2	0.024	0.231144	4.722826	1.419266	0.156987	0.366304	-5.31018
KLRC1	NM_002259.3	0.079	0.308205	4.143517	1.407404	0.16047	0.372656	-5.3263
TNFRSF14	NM_003820.2	0	0.074707	7.564073	1.400958	0.162387	0.375329	-5.33501
TICAM1	NM_014261.1	0.003	-0.15947	6.452073	-1.38576	0.166976	0.38251	-5.35539
GZMM	NM_005317.2	0.01	-0.19299	5.841592	-1.38344	0.167686	0.38251	-5.35848
CSF1R	NM_005211.2	0.164	0.367074	3.669342	1.381801	0.168187	0.38251	-5.36066
TNFRSF1B	NM_001066.2	0	0.12029	9.797379	1.37563	0.170089	0.38251	-5.36885
VEGFA	NM_001025366.1	0	0.170801	7.925177	1.37115	0.171479	0.38251	-5.37477
ATF2	NM_001256090.1	0	-0.07659	6.355255	-1.37055	0.171666	0.38251	-5.37556
MAP3K1	NM_005921.1	0	-0.08756	8.030733	-1.37039	0.171715	0.38251	-5.37577
LILRB1	NM_001081637.1	0.007	0.23053	6.524287	1.370314	0.171739	0.38251	-5.37587
CCND3	NM_001760.2	0	0.120015	8.801348	1.365466	0.173256	0.384141	-5.38225
TFRC	NM_003234.1	0	0.163735	7.850671	1.359198	0.17523	0.386769	-5.39047
TLR1	NM_003263.3	0	0.143132	7.545529	1.356638	0.176042	0.386818	-5.39382

CD180	NM_005582.2	0.164	-0.35978	3.296288	-1.33782	0.182094	0.397744	-5.41823
BCL10	NM_003921.2	0	-0.09699	8.121733	-1.33615	0.182637	0.397744	-5.42037
SIGIRR	NM_021805.2	0.007	0.162053	6.441101	1.330232	0.184577	0.400188	-5.42797
ETS1	NM_005238.3	0	-0.14402	9.109918	-1.30143	0.194231	0.419266	-5.46446
CD84	NM_001184879.1	0	0.103051	6.841624	1.296144	0.196045	0.421324	-5.47107
RELA	NM_021975.2	0	-0.11051	6.820764	-1.2863	0.199453	0.424922	-5.48332
RUNX3	NM_004350.1	0	-0.14655	8.954162	-1.28533	0.199792	0.424922	-5.48452
ITGAL	NM_002209.2	0	0.110709	8.40379	1.282449	0.200797	0.424922	-5.48808
F13A1	NM_000129.3	0	0.208432	9.569633	1.281333	0.201188	0.424922	-5.48946
MAPK8	NM_002750.2	0.017	-0.17437	4.508	-1.27521	0.203341	0.427627	-5.49699
SELPLG	NM_001206609.1	0	0.180803	7.156874	1.25212	0.211621	0.443139	-5.52511
BTK	NM_000061.1	0	0.109534	6.738055	1.24502	0.214216	0.446662	-5.53365
CDKN1A	NM_000389.2	0	0.136136	9.091459	1.241019	0.215688	0.446902	-5.53845
ICAM4	NM_001039132.1	0.068	0.262216	3.664074	1.239754	0.216154	0.446902	-5.53996
IL16	NM_004513.4	0	-0.07198	7.722864	-1.22411	0.221988	0.457034	-5.55853
ITGA6	NM_000210.1	0.062	-0.26395	4.19563	-1.21486	0.225491	0.462304	-5.5694
CX3CR1	NM_001337.3	0.021	0.321416	5.610803	1.204303	0.229539	0.464849	-5.58171
TNFRSF13B	NM_012452.2	0.418	-0.31701	1.686095	-1.20343	0.229877	0.464849	-5.58273
CXCR4	NM_003467.2	0	-0.13112	11.62269	-1.20333	0.229915	0.464849	-5.58285
PSMB8	NM_004159.4	0	0.082664	8.709897	1.201744	0.230527	0.464849	-5.58468
ITGAE	NM_002208.4	0	-0.12333	6.747701	-1.19167	0.234448	0.470818	-5.5963
ALCAM	NM_001627.3	0.048	0.211222	3.985995	1.187308	0.236161	0.472144	-5.60131
RORA	NM_134261.2	0.003	-0.17524	6.797615	-1.18509	0.237036	0.472144	-5.60384
PIK3CD	NM_005026.3	0	0.113814	7.459838	1.18224	0.238162	0.472467	-5.6071
CTLA4	NM_005214.3	0.01	0.195131	5.860546	1.176395	0.240485	0.475151	-5.61374
CD9	NM_001769.2	0	0.176341	6.234418	1.170875	0.242692	0.477587	-5.61999
CLEC7A	NM_197954.2	0	0.171825	8.481846	1.166744	0.244354	0.477732	-5.62465
CD86	NM_175862.3	0.024	0.206561	5.845976	1.165848	0.244716	0.477732	-5.62565
TNF	NM_000594.2	0	-0.16262	7.861256	-1.14891	0.25162	0.489261	-5.64456
SH2B2	NM_020979.3	0	0.140015	6.215647	1.146196	0.25274	0.489496	-5.64756

CD46	NM_172350.1	0	0.066854	8.729013	1.137536	0.256334	0.494502	-5.6571
TFE3	NM_006521.3	0.007	0.133184	4.911264	1.134183	0.257735	0.495255	-5.66077
TBK1	NM_013254.2	0	0.106346	6.272289	1.129884	0.259539	0.496773	-5.66547
MICB	NM_005931.3	0	-0.08566	6.878711	-1.12635	0.261028	0.497494	-5.66931
IFI35	NM_005533.3	0.027	0.252497	5.329995	1.123857	0.262083	0.497494	-5.67202
ICOS	NM_012092.2	0.048	-0.24291	5.292234	-1.12078	0.263389	0.497494	-5.67535
CXCL5	NM_002994.3	0.027	-0.263	5.612661	-1.1194	0.263976	0.497494	-5.67685
IRF7	NM_001572.3	0	-0.12638	7.657972	-1.10964	0.268153	0.503429	-5.68734
CTSL	NM_001912.4	0.205	0.301792	2.912495	1.105296	0.270026	0.503527	-5.69198
TNFSF12	NM_003809.2	0	0.10093	7.404735	1.104754	0.27026	0.503527	-5.69256
TNFRSF18	NM_004195.2	0.247	-0.24565	2.385066	-1.09407	0.274909	0.507626	-5.70389
TNFAIP3	NM_006290.2	0	0.156	11.40526	1.092742	0.275492	0.507626	-5.70529
IL2RG	NM_000206.1	0	-0.11863	8.597442	-1.0907	0.276387	0.507626	-5.70744
MAP2K2	NM_030662.2	0	0.076097	8.356256	1.089203	0.277046	0.507626	-5.70902
SELL	NR_029467.1	0	0.145559	9.719688	1.087853	0.277641	0.507626	-5.71043
NUP107	NM_020401.2	0.003	-0.08573	5.14324	-1.07134	0.284983	0.519115	-5.72764
TNFSF8	NM_001244.3	0.007	0.137647	5.546341	1.066498	0.287161	0.521145	-5.73263
TAB1	NM_153497.2	0.027	0.168658	4.727958	1.06405	0.288267	0.521221	-5.73515
GPI	NM_000175.2	0	0.064859	7.81144	1.059153	0.290487	0.523304	-5.74017
NFKB1	NM_003998.2	0.003	-0.12429	5.891648	-1.03959	0.29947	0.537509	-5.75998
PLAU	NM_002658.2	0.226	0.429511	4.195883	1.034839	0.30168	0.539501	-5.76474
TNFRSF8	NM_152942.2	0.373	-0.23021	1.712385	-1.02707	0.305317	0.544019	-5.77248
SOCS1	NM_003745.1	0.024	0.20106	5.279282	1.016683	0.310224	0.550298	-5.78272
CD5	NM_014207.2	0.007	-0.14696	7.202144	-1.01487	0.311087	0.550298	-5.7845
KLRK1	NM_007360.3	0.007	0.145483	8.067134	1.007153	0.314772	0.554815	-5.79203
PSMD7	NM_002811.3	0	-0.06573	8.443101	-0.98477	0.325626	0.570076	-5.81356
MAP4K2	NM_004579.2	0	-0.06663	6.797415	-0.98007	0.327938	0.570076	-5.81802
CD7	NM_006137.6	0.007	0.11356	6.90442	0.979467	0.328235	0.570076	-5.81859
SH2D1A	NM_001114937.2	0.024	0.177791	5.74523	0.977542	0.329185	0.570076	-5.82041
CD274	NM_014143.3	0.469	0.251653	1.515544	0.977416	0.329248	0.570076	-5.82053

CCR1	NM_001295.2	0.007	0.160784	6.178898	0.96233	0.336754	0.580469	-5.83466
HLA-DRA	NM_019111.3	0	0.089015	12.5209	0.960605	0.33762	0.580469	-5.83627
PRF1	NM_005041.3	0.007	0.177752	8.437403	0.956581	0.339644	0.581907	-5.83999
NT5E	NM_002526.2	0.137	-0.21835	2.742171	-0.94282	0.346625	0.591799	-5.85262
IFNAR1	NM_000629.2	0.171	0.214814	2.674679	0.938635	0.348766	0.593387	-5.85642
CD48	NM_001778.2	0	-0.08022	10.49278	-0.93497	0.350651	0.594388	-5.85974
IL1RN	NM_000577.3	0.003	-0.19334	7.56642	-0.93277	0.351781	0.594388	-5.86172
TCF7	NM_003202.2	0.021	-0.17797	7.021768	-0.92874	0.35386	0.594388	-5.86534
NCF4	NM_000631.4	0	0.128993	7.36641	0.928075	0.354207	0.594388	-5.86594
CSF3R	NM_156038.2	0	0.14935	10.23874	0.921454	0.357645	0.596571	-5.87186
CFD	NM_001928.2	0	0.124804	6.853647	0.920883	0.357943	0.596571	-5.87237
MSR1	NM_002445.3	0.082	0.246619	4.379792	0.912552	0.362301	0.601788	-5.87975
HMGB1	NM_002128.4	0	0.052205	9.277236	0.904732	0.366423	0.606578	-5.88661
BCL2L1	NM_001191.2	0	0.211185	8.230849	0.896804	0.370631	0.611479	-5.89351
BATF	NM_006399.3	0.021	0.137106	4.187868	0.890613	0.373938	0.614865	-5.89886
CARD11	NM_032415.2	0	0.089951	6.856218	0.877357	0.381081	0.624514	-5.91019
GZMB	NM_004131.3	0.003	0.135831	8.566658	0.864433	0.388126	0.633939	-5.92107
CD37	NM_001774.2	0.003	-0.07534	7.4318	-0.85578	0.392886	0.638186	-5.92826
MAPKAPK2	NM_004759.3	0	-0.09288	8.333636	-0.85498	0.393331	0.638186	-5.92892
ITGB3	NM_000212.2	0.003	0.173387	6.991195	0.849559	0.396331	0.640932	-5.93339
CD40LG	NM_000074.2	0.082	-0.18764	3.887451	-0.83753	0.403045	0.649645	-5.9432
IL23A	NM_016584.2	0.291	-0.19586	1.983131	-0.834	0.405027	0.650698	-5.94605
CYLD	NM_015247.1	0	-0.06649	8.50695	-0.82771	0.408572	0.65237	-5.9511
ICAM1	NM_000201.2	0	0.131221	7.861002	0.82738	0.40876	0.65237	-5.95137
LAMP1	NM_005561.3	0	0.04741	8.754433	0.825082	0.410061	0.65237	-5.9532
DDX58	NM_014314.3	0.031	-0.17794	5.32417	-0.81779	0.414209	0.656048	-5.959
TNFSF10	NM_003810.2	0	0.127017	7.480651	0.816309	0.415051	0.656048	-5.96016
TNFSF4	NM_003326.2	0.007	0.138104	4.998437	0.812449	0.417258	0.657416	-5.9632
IFIT2	NM_001547.4	0.003	0.182496	6.889349	0.802861	0.422769	0.663551	-5.97069
ENG	NM_001114753.1	0.051	0.165274	4.763187	0.800972	0.42386	0.663551	-5.97216

ANP32B	NM_006401.2	0	0.072397	8.430959	0.795511	0.427022	0.665921	-5.97637
KIR_Inhibiting_Subgroup_1	NM_014218.2	0.243	0.198002	2.723496	0.79367	0.428092	0.665921	-5.97778
LTB	NM_002341.1	0	-0.09931	9.500478	-0.78993	0.430267	0.667187	-5.98065
LAG3	NM_002286.5	0.065	0.18651	4.59753	0.787012	0.431973	0.667718	-5.98287
PSEN2	NM_000447.2	0.058	-0.14173	3.557444	-0.77797	0.437274	0.673787	-5.98971
PRKCD	NM_006254.3	0	0.08415	8.691483	0.770011	0.441974	0.677755	-5.99567
IRF2	NM_002199.3	0	0.091535	6.093233	0.768928	0.442616	0.677755	-5.99648
LAMP2	NM_001122606.1	0	0.060872	7.314486	0.765619	0.444579	0.678641	-5.99894
TNFRSF10C	NM_003841.3	0.106	0.252148	5.144526	0.750379	0.453687	0.688358	-6.01011
IFIH1	NM_022168.2	0.003	0.112874	6.062449	0.749364	0.454297	0.688358	-6.01085
SERPINB2	NM_002575.1	0.075	0.264692	5.633046	0.746413	0.456075	0.688358	-6.01298
CXCL3	NM_002090.2	0.267	0.269806	3.587643	0.745601	0.456564	0.688358	-6.01357
PIN1	NM_006221.2	0	0.050952	6.588471	0.737149	0.461679	0.693934	-6.01963
KLRB1	NM_002258.2	0.007	-0.1207	7.909012	-0.72968	0.466225	0.698117	-6.02493
CKLF	NM_181640.2	0	0.107452	7.308658	0.727902	0.467311	0.698117	-6.02618
BAX	NM_138761.3	0	0.038979	8.163919	0.713712	0.476028	0.708978	-6.03608
TBX21	NM_013351.1	0.007	-0.11193	6.936496	-0.70986	0.478412	0.709951	-6.03873
DUSP4	NM_057158.2	0.164	0.205445	3.536306	0.707973	0.479579	0.709951	-6.04002
RELB	NM_006509.2	0	-0.09121	6.86915	-0.7028	0.482795	0.712559	-6.04356
GTF3C1	NM_001520.3	0.007	0.066569	5.445361	0.69324	0.488761	0.717427	-6.05001
CD38	NM_001775.2	0.007	0.121672	5.552394	0.692824	0.489022	0.717427	-6.05029
IRF3	NM_001571.5	0.01	-0.07695	5.322492	-0.68509	0.493884	0.722314	-6.05545
KLRG1	NM_005810.3	0.051	0.166372	5.268751	0.679453	0.497439	0.722314	-6.05917
DPP4	NM_001935.3	0.366	-0.15601	1.721784	-0.6752	0.500134	0.722314	-6.06196
FOXJ1	NM_001454.3	0	-0.09016	5.448176	-0.66972	0.503613	0.722314	-6.06552
EGR2	NM_000399.3	0.164	-0.22262	3.880796	-0.66774	0.504876	0.722314	-6.0668
CXCR2	NM_001557.2	0.058	-0.21933	6.028551	-0.66629	0.505803	0.722314	-6.06774
CCL5	NM_002985.2	0	0.082077	10.74778	0.665168	0.506517	0.722314	-6.06846
CCR5	NM_000579.1	0.462	0.155936	1.57749	0.664095	0.507202	0.722314	-6.06915
CD3G	NM_000073.2	0.021	-0.1302	6.111588	-0.65975	0.509981	0.722314	-6.07193

IRAK2	NM_001570.3	0.021	-0.13135	4.751827	-0.65829	0.51092	0.722314	-6.07287
TIGIT	NM_173799.2	0.096	-0.15282	3.561425	-0.65682	0.511862	0.722314	-6.0738
IL2RB	NM_000878.2	0.014	0.109737	7.075061	0.655099	0.512968	0.722314	-6.07489
PPBP	NM_002704.2	0	0.115215	11.85189	0.654403	0.513415	0.722314	-6.07533
PRKCE	NM_005400.2	0.151	-0.14312	3.082404	-0.65371	0.513861	0.722314	-6.07577
IRAK4	NM_016123.1	0.007	0.075115	5.477751	0.652772	0.514465	0.722314	-6.07637
C1QBP	NM_001212.3	0	-0.05194	7.336941	-0.64573	0.519007	0.72661	-6.08079
FUT7	NM_004479.3	0.058	0.160506	4.629216	0.642962	0.5208	0.727043	-6.08252
SLAMF6	NM_001184714.1	0.267	0.174256	2.493996	0.639	0.523371	0.728556	-6.08498
IRAK1	NM_001569.3	0	0.046043	7.226552	0.63211	0.527856	0.729807	-6.08921
MAF	NM_005360.4	0.021	0.102955	5.451789	0.631047	0.528549	0.729807	-6.08986
LY86	NM_004271.3	0	0.06966	7.733498	0.630759	0.528738	0.729807	-6.09004
TAP1	NM_000593.5	0	0.061778	7.406064	0.621813	0.534595	0.735819	-6.09546
ITK	NM_005546.3	0.007	-0.08546	7.219445	-0.61307	0.540351	0.741658	-6.10069
IGF1R	NM_000875.2	0	-0.09329	6.296926	-0.59851	0.550005	0.752801	-6.10922
JAK1	NM_002227.1	0	-0.02647	9.228152	-0.59358	0.553298	0.755198	-6.11207
ATF1	NM_005171.2	0.007	0.064509	5.483778	0.584195	0.559582	0.755281	-6.11742
KLRF1	NM_016523.1	0.086	0.128842	5.228966	0.582103	0.560989	0.755281	-6.1186
CD6	NM_006725.3	0.014	-0.09331	6.383183	-0.5817	0.561262	0.755281	-6.11883
MAP3K5	NM_005923.3	0	0.048765	7.51705	0.58132	0.561515	0.755281	-6.11904
KIR_Activating_Subgroup_1	NM_001083539.1	0.469	0.157441	1.847964	0.580201	0.562268	0.755281	-6.11967
CD8A	NM_001768.5	0	0.086842	7.865574	0.579697	0.562607	0.755281	-6.11996
NCAM1	NM_000615.5	0.017	0.090715	4.312272	0.576399	0.564831	0.755488	-6.1218
TXNIP	NM_006472.1	0	0.050863	12.29742	0.574896	0.565845	0.755488	-6.12264
LYN	NM_002350.1	0	-0.05401	8.412352	-0.56243	0.574293	0.763836	-6.1295
CD74	NM_001025159.1	0	0.044411	12.72202	0.561077	0.575215	0.763836	-6.13024
LTF	NM_002343.2	0.075	-0.18905	5.321943	-0.55319	0.580597	0.765386	-6.13449
ICOSLG	NM_015259.4	0.055	-0.11617	4.973823	-0.55096	0.582125	0.765386	-6.13569
HLA-E	NM_005516.4	0	-0.03671	11.6039	-0.5504	0.582508	0.765386	-6.13599
CMKLR1	NM_004072.1	0.301	-0.15721	2.531657	-0.54728	0.584642	0.765386	-6.13764

PDGFC	NM_016205.2	0.147	0.127587	3.027464	0.546938	0.584878	0.765386	-6.13782
NFKB2	NM_002502.2	0	-0.08354	9.49988	-0.54566	0.585755	0.765386	-6.1385
TIRAP	NM_148910.2	0.144	0.10212	2.624071	0.539999	0.589648	0.768424	-6.14147
SH2D1B	NM_053282.4	0.205	0.127367	3.706056	0.531929	0.595217	0.772076	-6.14566
CCL4	NM_002984.2	0	-0.10204	7.435982	-0.53137	0.595602	0.772076	-6.14595
TAPBP	NM_003190.4	0	-0.03953	9.857129	-0.52364	0.600964	0.775909	-6.1499
OSM	NM_020530.4	0.014	0.103228	6.986592	0.522543	0.601726	0.775909	-6.15045
SLAMF7	NM_021181.3	0	-0.0631	6.269028	-0.51533	0.606749	0.780333	-6.15408
IL6	NM_000600.1	0.027	-0.1025	5.194713	-0.5113	0.609566	0.781904	-6.15608
NOTCH1	NM_017617.3	0.007	0.065892	6.163288	0.497987	0.618903	0.791808	-6.16259
LY96	NM_015364.2	0	0.068447	7.682891	0.49385	0.621818	0.792174	-6.16458
MAP2K4	NM_003010.2	0.171	0.114138	2.860254	0.492993	0.622422	0.792174	-6.16499
CD99	NM_002414.3	0	0.039699	10.01378	0.487579	0.626248	0.794978	-6.16756
IL1RAP	NM_002182.2	0	0.082964	6.01225	0.478809	0.632466	0.800797	-6.17166
MAVS	NM_020746.3	0.031	0.079156	4.40858	0.474239	0.635717	0.802838	-6.17377
CREB1	NM_004379.3	0	-0.04394	5.91797	-0.46567	0.64183	0.808475	-6.17767
ITGA4	NM_000885.4	0	-0.03618	7.624805	-0.45906	0.646562	0.812347	-6.18063
NFATC2	NM_012340.3	0	-0.04736	7.254581	-0.45645	0.648437	0.81262	-6.18179
CD247	NM_198053.1	0.007	-0.0648	7.876366	-0.45124	0.652183	0.815228	-6.18408
CCR7	NM_001838.2	0.113	-0.10635	3.873809	-0.44737	0.654971	0.81663	-6.18576
IL7R	NM_002185.2	0.01	0.074895	7.407723	0.443184	0.657991	0.818314	-6.18757
CTSW	NM_001335.3	0.003	-0.06211	7.625144	-0.43115	0.666705	0.825267	-6.19266
FCGR3A	NM_000569.6	0	0.063344	8.819493	0.430815	0.66695	0.825267	-6.1928
CD70	NM_001252.2	0.346	0.099249	1.907375	0.422646	0.672894	0.828542	-6.19618
CD2	NM_001767.3	0.072	-0.09664	4.100804	-0.42253	0.672979	0.828542	-6.19623
GNLY	NM_006433.2	0.003	0.072766	9.647454	0.416881	0.6771	0.831526	-6.19852
KIR3DL2	NM_006737.2	0.253	0.101491	2.487052	0.409278	0.682664	0.836263	-6.20157
HLA-DPA1	NM_033554.2	0	0.041226	10.29383	0.398379	0.690669	0.842505	-6.20583
CCR6	NM_031409.2	0.301	0.089742	2.05276	0.396606	0.691975	0.842505	-6.20651
STAT4	NM_003151.2	0.003	0.055443	6.168636	0.395326	0.692918	0.842505	-6.20701

PLA2G6	NM_001004426.1	0.034	0.062979	4.049692	0.374932	0.708008	0.858723	-6.21462
ICAM2	NM_000873.3	0	0.038713	6.419583	0.370028	0.711655	0.861015	-6.21639
MAP3K7	NM_145333.1	0	-0.02225	6.651272	-0.36331	0.716661	0.864935	-6.21877
CD8B	NM_004931.3	0.024	-0.06884	5.952351	-0.35804	0.720594	0.866057	-6.22061
HLA-A	NM_002116.5	0	-0.02245	13.45711	-0.35616	0.722004	0.866057	-6.22126
NOD1	NM_006092.1	0.082	0.070752	3.62329	0.35497	0.722892	0.866057	-6.22167
TREM1	NM_018643.3	0	0.05993	9.971133	0.351419	0.725551	0.867086	-6.22289
HLA-C	NM_002117.4	0	-0.02462	12.6451	-0.34772	0.728322	0.867086	-6.22414
HLA-G	NM_002127.4	0	0.027393	7.159786	0.34674	0.72906	0.867086	-6.22447
FCER1A	NM_002001.2	0.12	-0.10606	4.494639	-0.34261	0.732159	0.868663	-6.22585
CD83	NM_004233.3	0	-0.04348	10.15734	-0.33821	0.735468	0.870481	-6.2273
ATG16L1	NM_198890.2	0.007	0.038797	5.877359	0.332187	0.740008	0.873745	-6.22926
SMAD2	NM_005901.5	0	-0.02256	6.605892	-0.31467	0.753261	0.887255	-6.23475
HLA-DQB1	NM_002123.3	0.315	0.146759	5.682745	0.305144	0.760494	0.887905	-6.23761
CD55	NM_000574.3	0.264	-0.0769	2.574407	-0.30312	0.762034	0.887905	-6.23821
CCR3	NM_001837.2	0.455	0.07209	1.557105	0.302895	0.762206	0.887905	-6.23827
CCL3	NM_002983.2	0	0.043411	9.06783	0.302508	0.7625	0.887905	-6.23839
CASP3	NM_032991.2	0.007	-0.03391	5.030588	-0.29985	0.764524	0.887905	-6.23916
REL	NM_002908.2	0	0.029579	7.632005	0.298811	0.765316	0.887905	-6.23946
JAM3	NM_032801.3	0.027	0.067402	4.903156	0.297262	0.766497	0.887905	-6.23991
IFNG	NM_000619.2	0.007	-0.0556	6.625883	-0.28737	0.774054	0.89368	-6.24271
ITCH	NM_001257138.1	0	0.019578	8.069219	0.285335	0.775608	0.89368	-6.24328
CD96	NM_005816.4	0.007	0.039741	6.991743	0.283576	0.776955	0.89368	-6.24376
TRAF3	NM_145725.1	0	-0.02097	7.01356	-0.27291	0.78513	0.900969	-6.24664
IFNAR2	NM_000874.3	0	-0.0178	8.301537	-0.25417	0.799561	0.915386	-6.25142
NFKBIA	NM_020529.1	0	-0.03524	13.49199	-0.2503	0.802547	0.916662	-6.25237
SLAMF1	NM_003037.2	0.205	0.056232	2.599121	0.238585	0.81161	0.922816	-6.25515
ELK1	NM_005229.3	0.031	-0.03834	4.493141	-0.23847	0.811702	0.922816	-6.25517
CD58	NM_001779.2	0	0.019209	7.622745	0.234017	0.815151	0.924593	-6.25619
ICAM3	NM_002162.3	0	-0.01441	9.390235	-0.22991	0.818337	0.926063	-6.25711

EGR1	NM_001964.2	0.031	-0.06297	6.703873	-0.22559	0.821694	0.927719	-6.25807
PIK3CG	NM_002649.2	0.003	0.021167	7.079316	0.220663	0.825523	0.928016	-6.25913
IL32	NM_001012633.1	0	-0.02787	10.97232	-0.22038	0.825745	0.928016	-6.2592
IFITM2	NM_006435.2	0	0.027574	10.97691	0.21571	0.829378	0.929966	-6.26018
CD81	NM_004356.3	0	-0.01507	9.013876	-0.21286	0.8316	0.930329	-6.26077
IL1B	NM_000576.2	0.031	-0.0632	6.876044	-0.20496	0.837758	0.933469	-6.26237
MICA	NM_000247.1	0.178	0.045088	2.747806	0.204374	0.838217	0.933469	-6.26249
GZMH	NM_033423.3	0.007	-0.03749	8.230464	-0.18699	0.851814	0.944169	-6.26579
BID	NM_001196.2	0.038	0.023479	5.419228	0.184212	0.853987	0.944169	-6.26629
TANK	NM_004180.2	0	0.016213	8.704286	0.183679	0.854405	0.944169	-6.26638
XCL2	NM_003175.3	0.038	0.040662	5.62487	0.181733	0.85593	0.944169	-6.26673
IFI27	NM_005532.3	0.202	-0.08698	4.944098	-0.17978	0.85746	0.944169	-6.26707
CD28	NM_001243078.1	0.178	0.045639	3.234351	0.177082	0.859578	0.944379	-6.26753
IL2RA	NM_000417.1	0.318	0.03794	1.886076	0.173342	0.862514	0.945485	-6.26817
IL12RB1	NM_005535.1	0	-0.01462	5.68807	-0.16419	0.869706	0.949836	-6.26967
ISG20	NM_002201.4	0	-0.01739	7.838453	-0.16336	0.87036	0.949836	-6.2698
HLA-B	NM_005514.6	0	0.00947	13.57856	0.159285	0.873565	0.951215	-6.27044
CD164	NM_006016.4	0	-0.01112	8.347077	-0.15547	0.876566	0.951447	-6.27102
ABCB1	NM_000927.3	0.092	0.034371	3.909035	0.151775	0.879479	0.951447	-6.27157
CCL3L1	NM_021006.4	0	0.021425	8.932008	0.148787	0.881834	0.951447	-6.272
TP53	NM_000546.2	0.014	0.024703	5.277781	0.147987	0.882465	0.951447	-6.27212
ADA	NM_000022.2	0.007	0.018869	5.724951	0.146691	0.883487	0.951447	-6.2723
IL4R	NM_000418.2	0	-0.01432	7.572045	-0.13982	0.888905	0.953758	-6.27326
CSF1	NM_000757.4	0.236	0.037627	2.980229	0.133434	0.893951	0.953758	-6.2741
IL8	NM_000584.2	0	0.028588	13.13576	0.133118	0.8942	0.953758	-6.27414
FOS	NM_005252.2	0	-0.014	12.90851	-0.13092	0.895937	0.953758	-6.27443
HLA-DRB4	NM_021983.4	0.586	0.073017	3.564225	0.130382	0.896362	0.953758	-6.27449
CD3E	NM_000733.2	0.003	-0.01844	7.960763	-0.12918	0.897311	0.953758	-6.27464
TLR7	NM_016562.3	0.473	0.028725	1.375682	0.124809	0.900769	0.955361	-6.27518
LCK	NM_005356.2	0.003	0.015288	7.600179	0.119009	0.905358	0.955373	-6.27587

EP300	NM_001429.2	0	0.008872	8.468199	0.118006	0.906152	0.955373	-6.27598
CASP8	NM_001228.4	0	0.013473	8.914187	0.117402	0.90663	0.955373	-6.27605
MEF2C	NM_002397.3	0.01	0.022107	6.102815	0.114295	0.90909	0.95591	-6.2764
IL21R	NM_021798.2	0.082	-0.02388	3.908427	-0.10849	0.913691	0.95869	-6.27703
CXCR1	NM_000634.2	0.38	0.029819	2.291848	0.103293	0.917808	0.959785	-6.27756
ILF3	NM_001137673.1	0	-0.00636	8.574618	-0.10083	0.919762	0.959785	-6.27781
PTGS2	NM_000963.1	0.034	-0.02776	6.713789	-0.09976	0.92061	0.959785	-6.27791
HLA-DPB1	NM_002121.4	0	0.008911	10.31264	0.090592	0.927885	0.964743	-6.27876
CD160	NM_007053.2	0.171	0.025251	3.418159	0.088165	0.929812	0.964743	-6.27897
NCR1	NM_004829.5	0.017	0.015649	5.426898	0.086325	0.931272	0.964743	-6.27912
TNFRSF10B	NM_003842.3	0.014	0.009215	5.712225	0.080559	0.935853	0.967443	-6.27959
MR1	NM_001531.2	0.062	0.013608	3.313179	0.075356	0.939988	0.969671	-6.27998
UBC	NM_021009.3	0	0.005621	14.08619	0.071885	0.942747	0.970475	-6.28023
CD97	NM_078481.2	0	0.005598	10.53952	0.068805	0.945197	0.970957	-6.28044
CXCR3	NM_001504.1	0.11	-0.01382	3.638518	-0.06107	0.95135	0.973516	-6.28093
PECAM1	NM_000442.3	0	-0.00519	9.144289	-0.06068	0.951661	0.973516	-6.28095
TGFB1	NM_000660.3	0	0.00438	10.95069	0.052625	0.95807	0.977248	-6.28139
POU2F2	NM_002698.2	0.003	0.005652	6.756974	0.04907	0.9609	0.977248	-6.28157
CD3D	NM_000732.4	0.003	-0.00733	8.308636	-0.04858	0.961293	0.977248	-6.28159
CD1C	NM_001765.2	0.113	0.009095	4.009041	0.033189	0.973549	0.985803	-6.2822
PSMB7	NM_002799.2	0	0.001904	8.642375	0.032958	0.973732	0.985803	-6.28221
BST2	NM_004335.2	0	-0.00305	6.971434	-0.02816	0.977558	0.986877	-6.28235
KLRD1	NM_002262.3	0.014	0.005411	6.719953	0.026573	0.978821	0.986877	-6.28239
TARP	NM_001003799.1	0.003	0.003025	6.887268	0.020198	0.983901	0.989914	-6.28254
MME	NM_000902.2	0.216	0.006527	4.285765	0.017722	0.985874	0.989914	-6.28258
HLA-DQA1	NM_002122.3	0.325	0.007192	6.135714	0.013953	0.988878	0.9909	-6.28264
LCN2	NM_005564.3	0.007	0.000404	6.196887	0.001742	0.998611	0.998611	-6.28273
**C. D-Rd at C3D1 versus C1D1, CyTOF corrected**								
TICAM1	NM_014261.1	0.003	-1.21069	6.484478	-3.49476	0.001062	0.520404	-4.34817
TFE3	NM_006521.3	0.007	-1.39809	4.812652	-2.93857	0.005142	0.933739	-4.41643

NFATC1	NM_172389.1	0.007	-1.09311	5.167577	-2.76575	0.00815	0.933739	-4.4368
SH2B2	NM_020979.3	0	-1.08561	6.308864	-2.7514	0.008462	0.933739	-4.43847
CD40	NM_001250.4	0.021	1.439695	5.185361	2.705796	0.009528	0.933739	-4.44374
CD19	NM_001770.4	0.055	1.534883	4.931502	2.525745	0.015056	0.966676	-4.46414
CEBPB	NM_005194.2	0	-0.8497	9.624956	-2.46144	0.017651	0.966676	-4.47125
TXNIP	NM_006472.1	0	-0.73084	12.34162	-2.40301	0.020354	0.966676	-4.47763
CLEC4C	NM_203503.1	0.455	-1.95706	1.795366	-2.3847	0.021274	0.966676	-4.47961
CYBB	NM_000397.3	0	0.75861	9.618004	2.254127	0.028998	0.966676	-4.49346
BLK	NM_001715.2	0.12	1.572592	3.613267	2.196412	0.033144	0.966676	-4.49944
IGF1R	NM_000875.2	0	-1.08298	6.662979	-2.18286	0.034191	0.966676	-4.50083
HLA-DRB4	NM_021983.4	0.586	3.582443	3.214603	2.104246	0.040855	0.966676	-4.50877
IL1RAP	NM_002182.2	0	-1.17748	6.309927	-2.10279	0.040989	0.966676	-4.50892
CCRL2	NM_003965.4	0.072	1.249169	4.064196	2.100226	0.041225	0.966676	-4.50918
ADORA2A	NM_000675.3	0.003	-1.41942	6.821263	-2.06434	0.044655	0.966676	-4.51274
CXCR2	NM_001557.2	0.058	-1.62912	6.942205	-2.05026	0.046067	0.966676	-4.51412
DUSP6	NM_001946.2	0	1.000039	8.452191	2.034349	0.047709	0.966676	-4.51568
CD22	NM_001771.2	0.068	1.493466	5.510924	1.984124	0.053231	0.966676	-4.52054
NFKB2	NM_002502.2	0	-0.94178	9.750106	-1.96139	0.055906	0.966676	-4.52272
CD164	NM_006016.4	0	0.461634	8.324403	1.937604	0.058829	0.966676	-4.52497
TRAF6	NM_145803.1	0	-0.58915	6.417511	-1.91556	0.061654	0.966676	-4.52704
TNFRSF13C	NM_052945.3	0.045	1.218577	5.933964	1.892025	0.064796	0.966676	-4.52924
CD86	NM_175862.3	0.024	-1.26566	5.490634	-1.88416	0.065876	0.966676	-4.52997
NFKBIA	NM_020529.1	0	-0.90217	13.82902	-1.86501	0.068571	0.966676	-4.53173
THBD	NM_000361.2	0.003	-1.14782	8.602248	-1.85529	0.069974	0.966676	-4.53262
CD97	NM_078481.2	0	-0.46276	10.56114	-1.81775	0.075622	0.966676	-4.53603
OSM	NM_020530.4	0.014	-1.30762	7.354942	-1.78358	0.081095	0.966676	-4.53909
IL1RN	NM_000577.3	0.003	-1.37911	7.831008	-1.76929	0.083478	0.966676	-4.54036
IL1R2	NM_173343.1	0.027	-1.74557	6.622486	-1.76143	0.084815	0.966676	-4.54105
STAT1	NM_007315.2	0	0.630483	8.931527	1.731674	0.090038	0.966676	-4.54366
TAP2	NM_000544.3	0	0.462483	7.801693	1.72827	0.090652	0.966676	-4.54395

IL21R	NM_021798.2	0.082	-1.27301	3.672715	-1.7047	0.095001	0.966676	-4.54599
TREM1	NM_018643.3	0	-1.04106	10.27873	-1.67374	0.100974	0.966676	-4.54863
IL10RA	NM_001558.2	0	-0.44818	9.547293	-1.67164	0.10139	0.966676	-4.54881
ITGA2B	NM_000419.3	0	-0.96104	7.979841	-1.66416	0.102884	0.966676	-4.54944
PLA2G6	NM_001004426.1	0.034	0.72897	4.186342	1.640584	0.107709	0.966676	-4.55142
IL12RB1	NM_005535.1	0	0.478417	5.5927	1.626033	0.11078	0.966676	-4.55262
CD38	NM_001775.2	0.007	1.111632	5.538861	1.592445	0.118139	0.966676	-4.55538
TNFAIP3	NM_006290.2	0	-0.72107	11.64834	-1.58864	0.118997	0.966676	-4.55569
ECSIT	NM_001142464.2	0.003	0.699679	4.991808	1.573135	0.122546	0.966676	-4.55695
HLA-DRB3	NM_022555.3	0	0.515176	11.11413	1.569918	0.123293	0.966676	-4.55721
ITGA5	NM_002205.2	0	-0.45042	7.247719	-1.54976	0.128058	0.966676	-4.55882
CD36	NM_001001548.2	0	0.463599	9.11305	1.522948	0.134622	0.966676	-4.56094
KLRG1	NM_005810.3	0.051	0.985724	5.462645	1.517234	0.136055	0.966676	-4.56139
NOTCH1	NM_017617.3	0.007	-0.58336	6.291812	-1.51577	0.136423	0.966676	-4.56151
MFGE8	NM_001114614.1	0.055	-1.09089	3.889892	-1.5026	0.139782	0.966676	-4.56253
BCL10	NM_003921.2	0	-0.37489	8.084753	-1.50062	0.140291	0.966676	-4.56269
CD79B	NM_021602.2	0	0.73276	6.988636	1.468895	0.148672	0.966676	-4.56513
IL13RA1	NM_001560.2	0	-0.72096	7.264141	-1.46827	0.148841	0.966676	-4.56518
CD37	NM_001774.2	0.003	-0.72211	7.261653	-1.46475	0.149795	0.966676	-4.56544
CD79A	NM_001783.3	0.003	0.729146	7.370404	1.462169	0.150498	0.966676	-4.56564
SLC11A1	NM_000578.2	0	-0.58162	8.524329	-1.46135	0.150722	0.966676	-4.5657
CSF3R	NM_156038.2	0	-0.83159	10.61811	-1.46102	0.150812	0.966676	-4.56573
RELA	NM_021975.2	0	-0.38826	6.773554	-1.45976	0.151158	0.966676	-4.56582
CCR7	NM_001838.2	0.113	-1.07554	4.026042	-1.45677	0.151978	0.966676	-4.56605
MAP4K2	NM_004579.2	0	-0.35266	6.850069	-1.44845	0.154277	0.966676	-4.56668
SH2D1B	NM_053282.4	0.205	-1.21591	3.51781	-1.4425	0.155941	0.966676	-4.56713
BATF	NM_006399.3	0.021	-0.72197	4.086645	-1.42678	0.160401	0.966676	-4.5683
REL	NM_002908.2	0	-0.49236	7.611904	-1.41659	0.163343	0.966676	-4.56906
CTSL	NM_001912.4	0.205	1.448571	2.654839	1.402317	0.167538	0.966676	-4.57011
AKT3	NM_181690.1	0.003	-0.57013	6.175368	-1.39865	0.168628	0.966676	-4.57038

FCER2	NM_002002.4	0.082	1.017635	4.838727	1.393812	0.170076	0.966676	-4.57073
ITGB3	NM_000212.2	0.003	-1.02619	7.078078	-1.3936	0.170141	0.966676	-4.57075
TNF	NM_000594.2	0	-0.69026	7.922257	-1.39156	0.170755	0.966676	-4.5709
CFD	NM_001928.2	0	-0.67912	7.118607	-1.39113	0.170883	0.966676	-4.57093
DUSP4	NM_057158.2	0.164	-1.34182	3.60756	-1.37713	0.175141	0.966676	-4.57194
IL11RA	NM_147162.1	0.161	0.946338	3.286179	1.376933	0.175202	0.966676	-4.57196
GZMM	NM_005317.2	0.01	0.652385	5.838119	1.370053	0.177325	0.966676	-4.57246
CD74	NM_001025159.1	0	0.35519	12.54212	1.362883	0.179558	0.966676	-4.57297
LYN	NM_002350.1	0	-0.48819	8.436436	-1.35714	0.181363	0.966676	-4.57338
CD2	NM_001767.3	0.072	1.107239	4.124985	1.35197	0.183	0.966676	-4.57375
PRKCE	NM_005400.2	0.151	-1.06852	2.835834	-1.34275	0.185946	0.966676	-4.5744
ELK1	NM_005229.3	0.031	-0.67682	4.410011	-1.33215	0.189379	0.966676	-4.57515
MS4A1	NM_152866.2	0.051	1.01409	5.957998	1.328473	0.190579	0.966676	-4.57541
BTLA	NM_181780.2	0.034	0.830699	4.925785	1.315702	0.194798	0.966676	-4.5763
THBS1	NM_003246.2	0	-0.96911	10.14454	-1.31195	0.196051	0.966676	-4.57656
IFI35	NM_005533.3	0.027	1.007662	5.504062	1.311	0.196369	0.966676	-4.57663
PDGFC	NM_016205.2	0.147	-0.92684	3.22263	-1.30806	0.197355	0.966676	-4.57683
IL18RAP	NM_003853.2	0.065	-1.13865	5.650186	-1.30197	0.199411	0.966676	-4.57725
TNFRSF1B	NM_001066.2	0	-0.45883	9.713878	-1.29805	0.200744	0.966676	-4.57752
IL18	NM_001562.2	0.024	-0.71499	5.156855	-1.29729	0.201003	0.966676	-4.57757
ANXA1	NM_000700.1	0	0.444556	11.06642	1.281628	0.2064	0.966676	-4.57864
CD274	NM_014143.3	0.469	1.103878	1.426443	1.279311	0.207207	0.966676	-4.5788
ARG1	NM_000045.2	0.408	1.217947	2.621635	1.278503	0.207489	0.966676	-4.57885
CXCL16	NM_001100812.1	0.003	-0.67901	7.176502	-1.27664	0.20814	0.966676	-4.57898
CD3G	NM_000073.2	0.021	0.884528	6.097442	1.268007	0.21118	0.966676	-4.57956
S100B	NM_006272.1	0.5	0.985865	2.099881	1.262216	0.213237	0.966676	-4.57995
LAIR2	NM_002288.3	0.014	0.677743	4.742557	1.250116	0.217584	0.966676	-4.58076
IFNGR1	NM_000416.1	0	-0.40018	8.702158	-1.24931	0.217877	0.966676	-4.58081
IFITM2	NM_006435.2	0	-0.58133	11.276	-1.24079	0.220978	0.966676	-4.58138
FEZ1	NM_005103.4	0.414	-0.92888	1.798337	-1.23713	0.22232	0.966676	-4.58162

CX3CR1	NM_001337.3	0.021	1.068401	5.709481	1.228262	0.2256	0.966676	-4.5822
CCND3	NM_001760.2	0	-0.44014	8.792868	-1.22436	0.227053	0.966676	-4.58246
CCR5	NM_000579.1	0.462	0.971388	1.470938	1.223312	0.227446	0.966676	-4.58253
ICOSLG	NM_015259.4	0.055	-0.90246	4.625151	-1.22195	0.227955	0.966676	-4.58262
TGFB1	NM_000660.3	0	-0.39576	10.97278	-1.21308	0.231296	0.966676	-4.58319
PIK3CG	NM_002649.2	0.003	-0.30769	7.102338	-1.20086	0.235955	0.966676	-4.58398
CLEC7A	NM_197954.2	0	-0.53339	8.867019	-1.19922	0.236586	0.966676	-4.58408
NLRP3	NM_001079821.2	0	-0.51367	7.962799	-1.19375	0.238699	0.966676	-4.58444
CYFIP2	NM_001037332.2	0	0.375732	7.808367	1.192048	0.23936	0.966676	-4.58454
BCL2	NM_000657.2	0	0.424431	6.051298	1.188692	0.240665	0.966676	-4.58476
ICAM1	NM_000201.2	0	-0.62006	8.015949	-1.18636	0.241577	0.966676	-4.58491
BMI1	NM_005180.5	0	-0.47615	8.109578	-1.1782	0.244781	0.966676	-4.58542
CAMP	NM_004345.3	0.253	-1.21435	3.309635	-1.17491	0.246081	0.966676	-4.58563
TLR2	NM_003264.3	0	-0.49999	7.830566	-1.1748	0.246126	0.966676	-4.58564
XCL2	NM_003175.3	0.038	-0.85933	5.603926	-1.16877	0.248521	0.966676	-4.58602
BLNK	NM_013314.2	0.432	0.789848	1.742082	1.167782	0.248916	0.966676	-4.58608
HLA-B	NM_005514.6	0	-0.27706	13.51642	-1.15754	0.253029	0.966676	-4.58672
IL10	NM_000572.2	0.253	-0.89241	3.047345	-1.15681	0.253324	0.966676	-4.58676
FPR2	NM_001462.3	0.041	-0.8063	5.454238	-1.15619	0.253576	0.966676	-4.5868
ATF1	NM_005171.2	0.007	0.536291	5.417584	1.153602	0.254626	0.966676	-4.58696
IL23A	NM_016584.2	0.291	-0.93591	2.108989	-1.14767	0.257043	0.966676	-4.58733
IRF3	NM_001571.5	0.01	0.509539	5.238257	1.146567	0.257494	0.966676	-4.5874
TRAF3	NM_145725.1	0	-0.3199	7.125145	-1.14172	0.259484	0.966676	-4.58769
GZMA	NM_006144.2	0.01	0.838222	8.241182	1.140358	0.260044	0.966676	-4.58778
PSMB10	NM_002801.2	0	0.255618	8.863405	1.13251	0.263293	0.966676	-4.58826
PSMB8	NM_004159.4	0	0.304627	8.725179	1.125226	0.266334	0.966676	-4.5887
C1QA	NM_015991.2	0.127	-0.90696	4.005041	-1.12353	0.267045	0.966676	-4.5888
SELL	NR_029467.1	0	-0.47272	9.916742	-1.12091	0.268146	0.966676	-4.58896
HLA-E	NM_005516.4	0	-0.26993	11.62095	-1.11889	0.268998	0.966676	-4.58908
CD200	NM_005944.5	0.226	0.772615	2.747506	1.109018	0.273191	0.966676	-4.58968

FCGR2B	NM_001002273.1	0.048	0.679396	5.255136	1.104241	0.275235	0.966676	-4.58996
KLRF1	NM_016523.1	0.086	-0.83951	5.565089	-1.10003	0.277047	0.966676	-4.59021
DDX58	NM_014314.3	0.031	0.692713	5.685544	1.0981	0.277879	0.966676	-4.59033
PSMB9	NM_002800.4	0	0.398139	8.72407	1.094333	0.27951	0.966676	-4.59055
STAT6	NM_003153.3	0	-0.38459	9.391131	-1.09166	0.280671	0.966676	-4.59071
TICAM2	NM_021649.4	0.158	0.953162	2.653328	1.08968	0.281533	0.966676	-4.59082
MYD88	NM_002468.3	0	0.346512	8.569967	1.089615	0.281562	0.966676	-4.59083
BAX	NM_138761.3	0	0.223957	8.065971	1.077143	0.287037	0.966676	-4.59156
CYLD	NM_015247.1	0	-0.29501	8.487604	-1.07005	0.290182	0.966676	-4.59197
GZMK	NM_002104.2	0.01	0.694104	6.677627	1.068768	0.290754	0.966676	-4.59204
CD28	NM_001243078.1	0.178	-0.87615	3.178077	-1.06092	0.29427	0.966676	-4.5925
SMAD3	NM_005902.3	0	-0.40931	6.1692	-1.06002	0.294675	0.966676	-4.59255
CSF1R	NM_005211.2	0.164	1.048766	3.562819	1.057098	0.29599	0.966676	-4.59272
CXCR4	NM_003467.2	0	-0.36274	11.71915	-1.05629	0.296357	0.966676	-4.59276
VEGFA	NM_001025366.1	0	-0.41144	8.070343	-1.05066	0.298905	0.966676	-4.59308
STAT3	NM_139276.2	0	-0.29706	8.892825	-1.04176	0.302973	0.966676	-4.59359
TLR7	NM_016562.3	0.473	0.82941	1.309267	1.04101	0.303315	0.966676	-4.59363
TNFSF13	NM_003808.3	0	0.37276	6.751035	1.036681	0.305307	0.966676	-4.59387
LILRB3	NM_006864.2	0	-0.60964	8.053259	-1.035	0.306084	0.966676	-4.59397
TNFRSF1A	NM_001065.2	0	-0.33902	7.511156	-1.03198	0.307479	0.966676	-4.59414
IL15	NM_172174.1	0.024	0.451387	4.544888	1.030789	0.308033	0.966676	-4.59421
IFNAR1	NM_000629.2	0.171	-0.78159	2.773402	-1.0251	0.310681	0.966676	-4.59452
SH2D1A	NM_001114937.2	0.024	0.66963	5.607209	1.022751	0.311778	0.966676	-4.59465
S100A8	NM_002964.3	0	0.524137	13.06959	1.018894	0.313586	0.966676	-4.59487
EGR1	NM_001964.2	0.031	0.897586	6.651427	1.015287	0.315283	0.966676	-4.59507
ST6GAL1	NM_003032.2	0.003	0.359546	7.072304	1.013199	0.316268	0.966676	-4.59518
IL2RA	NM_000417.1	0.318	-0.7237	2.036021	-1.01287	0.316423	0.966676	-4.5952
HLA-DPB1	NM_002121.4	0	0.315224	10.20357	1.009739	0.317906	0.966676	-4.59537
MME	NM_000902.2	0.216	-1.24272	5.016458	-1.00971	0.317919	0.966676	-4.59537
IRF4	NM_002460.1	0.003	-0.62267	6.239596	-1.00905	0.318234	0.966676	-4.59541

CD180	NM_005582.2	0.164	0.914516	2.984111	1.006203	0.319585	0.966676	-4.59557
HLA-DRA	NM_019111.3	0	0.296431	12.4152	1.002291	0.32145	0.966676	-4.59578
ADA	NM_000022.2	0.007	0.318975	5.661062	0.9933	0.325764	0.966676	-4.59627
TLR1	NM_003263.3	0	-0.35461	7.71714	-0.99276	0.326023	0.966676	-4.5963
MX1	NM_002462.2	0	-0.55782	7.86973	-0.99035	0.327186	0.966676	-4.59643
CKLF	NM_181640.2	0	0.470493	7.453505	0.989596	0.327552	0.966676	-4.59647
ITGAX	NM_000887.3	0	-0.36772	9.221799	-0.98941	0.327644	0.966676	-4.59648
LY86	NM_004271.3	0	-0.3462	7.747927	-0.98918	0.327756	0.966676	-4.59649
FCGR2A	NM_021642.3	0	-0.48083	10.11777	-0.9791	0.332656	0.966676	-4.59703
CDKN1A	NM_000389.2	0	-0.31305	9.021987	-0.979	0.332703	0.966676	-4.59704
TFEB	NM_007162.2	0.014	-0.48768	5.550239	-0.97762	0.333378	0.966676	-4.59711
CXCL3	NM_002090.2	0.267	1.29938	3.780252	0.975061	0.334633	0.966676	-4.59725
IL2RG	NM_000206.1	0	0.340539	8.710508	0.974496	0.33491	0.966676	-4.59728
IRF5	NM_002200.3	0	-0.29627	6.435055	-0.9657	0.339245	0.966676	-4.59774
CASP8	NM_001228.4	0	-0.38002	9.094482	-0.96458	0.339801	0.966676	-4.5978
MR1	NM_001531.2	0.062	-0.62679	3.474107	-0.96319	0.340494	0.966676	-4.59788
LTBR	NM_002342.1	0.007	-0.45403	6.427198	-0.95889	0.342632	0.966676	-4.5981
CCR2	NM_001123041.2	0.175	0.878435	3.386746	0.95629	0.343928	0.966676	-4.59824
CARD9	NM_052813.4	0.089	-0.6378	4.147282	-0.95208	0.346037	0.966676	-4.59846
BTK	NM_000061.1	0	0.224839	6.784176	0.949364	0.3474	0.966676	-4.5986
S100A12	NM_005621.1	0	0.440536	10.56909	0.949057	0.347554	0.966676	-4.59861
ITGA6	NM_000210.1	0.062	0.53556	4.602096	0.947642	0.348267	0.966676	-4.59869
KLRC2	NM_002260.3	0.134	-0.76327	4.132589	-0.94674	0.348722	0.966676	-4.59873
CD27	NM_001242.4	0.031	0.6659	5.179405	0.945609	0.349292	0.966676	-4.59879
SPN	NM_003123.3	0.017	0.557062	5.348767	0.94298	0.350621	0.966676	-4.59893
MAF	NM_005360.4	0.021	0.643818	5.119079	0.934709	0.354822	0.966676	-4.59935
EP300	NM_001429.2	0	-0.26477	8.483865	-0.93229	0.356056	0.966676	-4.59948
IKBKG	NM_003639.2	0.003	-0.2412	6.422169	-0.93084	0.356802	0.966676	-4.59955
PLAU	NM_002658.2	0.226	-1.30662	4.97886	-0.92027	0.362233	0.966676	-4.60009
SLAMF7	NM_021181.3	0	0.4606	6.25628	0.919761	0.362499	0.966676	-4.60011

PSEN1	NM_000021.2	0	-0.23402	6.561164	-0.91909	0.362847	0.966676	-4.60015
JAK1	NM_002227.1	0	-0.19057	9.217217	-0.91401	0.365479	0.966676	-4.6004
CCL4	NM_002984.2	0	-0.47725	7.54716	-0.90999	0.367577	0.966676	-4.6006
IL4R	NM_000418.2	0	-0.41843	7.584634	-0.9076	0.368823	0.966676	-4.60072
TIGIT	NM_173799.2	0.096	0.672945	3.37671	0.907141	0.369063	0.966676	-4.60074
LILRA1	NM_006863.1	0.038	0.549535	5.268155	0.903658	0.370888	0.966676	-4.60092
GZMH	NM_033423.3	0.007	0.612791	8.19661	0.886928	0.379734	0.967393	-4.60174
MAPKAPK2	NM_004759.3	0	-0.3165	8.36488	-0.88651	0.379955	0.967393	-4.60176
LILRB1	NM_001081637.1	0.007	0.551154	6.376977	0.873022	0.387189	0.967393	-4.60241
MAPK3	NM_001040056.1	0	-0.26676	6.620495	-0.87176	0.38787	0.967393	-4.60247
IFNG	NM_000619.2	0.007	0.501867	6.698489	0.862662	0.392802	0.967393	-4.60291
IL6ST	NM_002184.2	0	-0.27123	8.370773	-0.85426	0.397391	0.967393	-4.6033
TXK	NM_003328.1	0.202	-0.58429	3.152687	-0.85365	0.397727	0.967393	-4.60333
CCL20	NM_004591.1	0.411	-0.78081	2.384007	-0.85328	0.397929	0.967393	-4.60335
MAP3K1	NM_005921.1	0	-0.20026	8.05806	-0.84916	0.400195	0.967393	-4.60354
HLA-A	NM_002116.5	0	-0.20606	13.41276	-0.84652	0.401646	0.967393	-4.60367
CCR4	NM_005508.4	0.062	-0.60262	4.255131	-0.84616	0.401846	0.967393	-4.60368
TNFRSF10B	NM_003842.3	0.014	-0.3557	5.755992	-0.83729	0.406763	0.967393	-4.60409
HLA-DPA1	NM_033554.2	0	0.274373	10.19163	0.835104	0.407978	0.967393	-4.60419
TARP	NM_001003799.1	0.003	0.433794	6.786451	0.834963	0.408057	0.967393	-4.6042
PIN1	NM_006221.2	0	0.205641	6.481329	0.826965	0.412529	0.967393	-4.60457
PSEN2	NM_000447.2	0.058	-0.49675	3.563695	-0.8215	0.4156	0.967393	-4.60482
CXCL2	NM_002089.3	0.058	0.816366	6.548646	0.820867	0.415959	0.967393	-4.60485
C2	NM_000063.3	0.243	0.753681	2.476793	0.819284	0.416852	0.967393	-4.60492
KLRD1	NM_002262.3	0.014	0.606022	6.740085	0.81847	0.417312	0.967393	-4.60495
CD24	NM_013230.2	0.007	-0.53807	5.739555	-0.81466	0.419465	0.967393	-4.60512
IRAK1	NM_001569.3	0	-0.20676	7.154834	-0.81298	0.42042	0.967393	-4.6052
HLA-G	NM_002127.4	0	-0.28118	7.116824	-0.81139	0.421325	0.967393	-4.60527
BCL6	NM_001706.2	0	-0.38129	9.634073	-0.81042	0.421874	0.967393	-4.60532
FOXJ1	NM_001454.3	0	0.359681	5.445389	0.81002	0.422102	0.967393	-4.60533

ANP32B	NM_006401.2	0	0.288929	8.323172	0.803697	0.425708	0.967393	-4.60562
NCAM1	NM_000615.5	0.017	-0.43682	4.399241	-0.80312	0.426036	0.967393	-4.60564
IL8	NM_000584.2	0	-0.51918	13.63443	-0.8004	0.427596	0.967393	-4.60576
TRAF2	NM_021138.3	0.048	0.460446	3.805718	0.786482	0.435621	0.967393	-4.60637
TNFRSF10C	NM_003841.3	0.106	-0.70204	5.932921	-0.78223	0.43809	0.967393	-4.60656
POU2F2	NM_002698.2	0.003	0.248341	6.763863	0.781512	0.438508	0.967393	-4.60659
ABCB1	NM_000927.3	0.092	-0.43062	4.00911	-0.77937	0.439759	0.967393	-4.60668
TIRAP	NM_148910.2	0.144	0.615686	2.708772	0.778528	0.440247	0.967393	-4.60672
KLRC1	NM_002259.3	0.079	-0.5704	4.543482	-0.77549	0.442024	0.967393	-4.60685
CD70	NM_001252.2	0.346	0.589957	1.72365	0.768617	0.446052	0.967393	-4.60714
MEFV	NM_000243.2	0.003	-0.42497	7.438844	-0.76832	0.446224	0.967393	-4.60715
C3AR1	NM_004054.2	0.147	0.704398	4.120067	0.766152	0.447502	0.967393	-4.60725
ALCAM	NM_001627.3	0.048	-0.43967	4.039628	-0.76475	0.448328	0.967393	-4.6073
LAMP1	NM_005561.3	0	0.162194	8.732198	0.761379	0.450319	0.967393	-4.60745
LY9	NM_001033667.1	0	0.273601	6.85593	0.75754	0.452592	0.967393	-4.60761
CD81	NM_004356.3	0	0.238954	8.858433	0.75754	0.452592	0.967393	-4.60761
CTLA4	NM_005214.3	0.01	0.441818	5.919118	0.753899	0.454754	0.967393	-4.60776
MAP2K2	NM_030662.2	0	0.192116	8.313204	0.753493	0.454996	0.967393	-4.60778
SMAD2	NM_005901.5	0	0.212659	6.606409	0.743581	0.460913	0.967393	-4.60819
IFIT1	NM_001548.3	0.079	-0.65407	4.978397	-0.74305	0.461234	0.967393	-4.60821
CD84	NM_001184879.1	0	-0.21508	6.851062	-0.72924	0.469556	0.967393	-4.60878
CD244	NM_016382.2	0.017	0.461241	5.927828	0.728625	0.469927	0.967393	-4.6088
CD4	NM_000616.4	0	0.232629	7.707342	0.727868	0.470386	0.967393	-4.60883
ICAM4	NM_001039132.1	0.068	-0.54172	4.095334	-0.727	0.470911	0.967393	-4.60887
FOS	NM_005252.2	0	-0.29111	13.08453	-0.72556	0.471789	0.967393	-4.60892
TLR5	NM_003268.3	0.202	-0.62005	3.397969	-0.72356	0.473003	0.967393	-4.60901
ISG20	NM_002201.4	0	-0.26048	7.953218	-0.72204	0.473929	0.967393	-4.60907
MIF	NM_002415.1	0	0.232805	8.051564	0.713908	0.478893	0.967393	-4.60939
RELB	NM_006509.2	0	-0.29434	6.880603	-0.70781	0.482636	0.967393	-4.60963
RIPK2	NM_003821.5	0	-0.28137	8.631872	-0.69767	0.488897	0.967393	-4.61003

LGALS3	NM_001177388.1	0	0.301185	9.422522	0.696755	0.489465	0.967393	-4.61006
IRF7	NM_001572.3	0	-0.28845	7.693078	-0.69654	0.489596	0.967393	-4.61007
SBNO2	NM_014963.2	0.003	-0.30674	6.650402	-0.69241	0.49216	0.967393	-4.61023
LILRB2	NM_005874.1	0	-0.26248	7.717561	-0.69024	0.493512	0.967393	-4.61031
LCK	NM_005356.2	0.003	0.422361	7.504193	0.688536	0.494576	0.967393	-4.61038
LILRA5	NM_181879.2	0	-0.31734	9.380127	-0.68539	0.496537	0.967393	-4.6105
HAVCR2	NM_032782.3	0.014	0.281889	5.163379	0.678973	0.500559	0.967393	-4.61074
TNFRSF18	NM_004195.2	0.247	0.524385	2.462623	0.677665	0.501381	0.967393	-4.61079
PVR	NM_006505.3	0.182	0.532449	2.690121	0.677455	0.501513	0.967393	-4.6108
GPI	NM_000175.2	0	0.157075	7.703165	0.674846	0.503154	0.967393	-4.6109
C1QBP	NM_001212.3	0	0.224017	7.333467	0.656212	0.514959	0.967393	-4.61159
TFRC	NM_003234.1	0	0.272634	7.865515	0.655594	0.515353	0.967393	-4.61161
TNFRSF9	NM_001561.4	0.329	-0.44476	1.979191	-0.65516	0.515632	0.967393	-4.61163
RPS6	NM_001010.2	0	0.203349	12.83735	0.65341	0.516747	0.967393	-4.61169
CTSS	NM_004079.3	0	0.165846	12.63254	0.651644	0.517876	0.967393	-4.61176
PIK3CD	NM_005026.3	0	-0.32411	7.383324	-0.64993	0.518973	0.967393	-4.61182
TNFRSF14	NM_003820.2	0	0.145319	7.563062	0.64914	0.519478	0.967393	-4.61185
TP53	NM_000546.2	0.014	0.332852	5.297269	0.64882	0.519683	0.967393	-4.61186
CD47	NM_001777.3	0	0.11663	8.848972	0.648048	0.520178	0.967393	-4.61189
ITGAL	NM_002209.2	0	0.196582	8.422515	0.645903	0.521553	0.967393	-4.61196
CD58	NM_001779.2	0	0.180404	7.654274	0.642841	0.523521	0.967393	-4.61207
IRF8	NM_002163.2	0	-0.20838	7.368256	-0.63916	0.525889	0.967393	-4.61221
CD48	NM_001778.2	0	0.183748	10.51527	0.636883	0.52736	0.967393	-4.61229
MEF2C	NM_002397.3	0.01	0.40749	6.144537	0.636784	0.527424	0.967393	-4.61229
LRP1	NM_002332.2	0	-0.2235	7.384461	-0.63488	0.528655	0.967393	-4.61236
IL6R	NM_000565.2	0	-0.22617	6.601497	-0.62938	0.532217	0.967393	-4.61255
CASP1	NM_001223.3	0	0.22689	8.0139	0.627706	0.533303	0.967393	-4.61261
TNFSF10	NM_003810.2	0	0.335017	7.566405	0.625915	0.534466	0.967393	-4.61267
STAT5B	NM_012448.3	0	-0.27783	7.056284	-0.62011	0.538247	0.967393	-4.61288
MICA	NM_000247.1	0.178	-0.4806	2.855832	-0.61946	0.538674	0.967393	-4.6129

ATG16L1	NM_198890.2	0.007	0.228282	5.810312	0.609585	0.545138	0.967393	-4.61324
IL32	NM_001012633.1	0	0.242986	11.05665	0.603698	0.549012	0.967393	-4.61344
CSF1	NM_000757.4	0.236	-0.52165	3.534624	-0.59524	0.554605	0.967393	-4.61372
UBC	NM_021009.3	0	-0.16844	14.13579	-0.59165	0.556983	0.967393	-4.61384
CD3D	NM_000732.4	0.003	0.397062	8.20291	0.590872	0.557501	0.967393	-4.61387
ENTPD1	NM_001098175.1	0.092	-0.42872	4.032419	-0.59031	0.557876	0.967393	-4.61388
MERTK	NM_006343.2	0.171	0.503313	2.777205	0.589604	0.558343	0.967393	-4.61391
F13A1	NM_000129.3	0	-0.34559	9.711828	-0.58475	0.561577	0.967393	-4.61407
TLR4	NM_138554.2	0.003	-0.26	6.848706	-0.57632	0.567212	0.967393	-4.61434
PSMD7	NM_002811.3	0	-0.12736	8.416579	-0.57547	0.567782	0.967393	-4.61437
NFATC3	NM_004555.2	0	0.166116	6.823623	0.572643	0.569677	0.967393	-4.61446
AMICA1	NM_153206.2	0	0.25202	9.092276	0.571893	0.570181	0.967393	-4.61448
PNMA1	NM_006029.4	0	-0.22958	5.394446	-0.57125	0.57061	0.967393	-4.6145
BID	NM_001196.2	0.038	-0.22157	5.321876	-0.56452	0.575143	0.967393	-4.61472
ATG7	NM_001136031.2	0.014	0.274431	5.794977	0.563732	0.575676	0.967393	-4.61474
CXCR1	NM_000634.2	0.38	-0.54958	2.902891	-0.56238	0.57659	0.967393	-4.61479
CD59	NM_000611.4	0	-0.28776	6.155392	-0.55579	0.58105	0.967393	-4.61499
MAP3K5	NM_005923.3	0	-0.15917	7.690549	-0.55398	0.582277	0.967393	-4.61505
PLAUR	NM_001005376.1	0	-0.2403	9.602724	-0.54846	0.586031	0.967393	-4.61522
TNFRSF8	NM_152942.2	0.373	0.413585	1.852716	0.548235	0.586183	0.967393	-4.61523
HLA-DQA1	NM_002122.3	0.325	0.751875	5.461318	0.547373	0.586771	0.967393	-4.61525
ITGAE	NM_002208.4	0	-0.16028	6.65954	-0.54437	0.588818	0.967393	-4.61534
CSF2RB	NM_000395.2	0	-0.2465	8.902947	-0.54156	0.590738	0.967393	-4.61543
ICAM2	NM_000873.3	0	-0.20871	6.481238	-0.53975	0.591973	0.967393	-4.61548
ICOS	NM_012092.2	0.048	0.333173	5.329691	0.534686	0.595444	0.967393	-4.61564
SLAMF1	NM_003037.2	0.205	-0.44393	2.548404	-0.52889	0.599426	0.967393	-4.61581
HMGB1	NM_002128.4	0	-0.10748	9.282678	-0.52791	0.600098	0.967393	-4.61584
STAT2	NM_005419.2	0	0.253285	7.744843	0.524494	0.602455	0.967393	-4.61594
BST2	NM_004335.2	0	0.205799	6.957766	0.524237	0.602632	0.967393	-4.61595
KIR3DL2	NM_006737.2	0.253	0.364515	2.403673	0.522529	0.603811	0.967393	-4.616

CD46	NM_172350.1	0	0.111207	8.867534	0.521569	0.604475	0.967393	-4.61603
FCGR1A	NM_000566.3	0.202	-0.40914	2.641645	-0.51393	0.609763	0.967393	-4.61625
YTHDF2	NM_001172828.1	0.01	-0.1339	5.948496	-0.5128	0.610547	0.967393	-4.61628
EGR2	NM_000399.3	0.164	0.574684	3.749044	0.503815	0.616797	0.967393	-4.61654
CREB1	NM_004379.3	0	0.172408	5.941505	0.498154	0.620751	0.967393	-4.6167
INPP5D	NM_005541.3	0	0.140262	7.652128	0.495993	0.622262	0.967393	-4.61676
BCL2L1	NM_001191.2	0	0.383814	8.045029	0.488808	0.627301	0.967393	-4.61696
NFATC2	NM_012340.3	0	0.143065	7.295299	0.486158	0.629165	0.967393	-4.61703
CXCR5	NM_001716.3	0.171	0.408885	3.160619	0.48381	0.630818	0.967393	-4.61709
CD63	NM_001780.4	0	-0.15499	9.814142	-0.48347	0.631057	0.967393	-4.6171
RUNX3	NM_004350.1	0	-0.17804	8.801292	-0.482	0.632091	0.967393	-4.61714
MAVS	NM_020746.3	0.031	0.236618	4.531542	0.477461	0.635297	0.967393	-4.61726
SELPLG	NM_001206609.1	0	0.230977	7.208536	0.476791	0.63577	0.967393	-4.61728
LTB	NM_002341.1	0	-0.23939	9.575613	-0.47513	0.636946	0.967393	-4.61733
NCF4	NM_000631.4	0	-0.20831	7.708288	-0.475	0.637038	0.967393	-4.61733
TBX21	NM_013351.1	0.007	0.255463	6.903745	0.474249	0.637568	0.967393	-4.61735
HCK	NM_002110.2	0	-0.20149	9.204422	-0.47344	0.638138	0.967393	-4.61737
ISG15	NM_005101.3	0	0.302912	7.06732	0.472332	0.638924	0.967393	-4.6174
NUP107	NM_020401.2	0.003	0.127423	5.270172	0.467563	0.642306	0.967393	-4.61753
ETS1	NM_005238.3	0	0.193647	9.049235	0.466834	0.642824	0.967393	-4.61755
KLRB1	NM_002258.2	0.007	-0.30415	7.841228	-0.4662	0.643275	0.967393	-4.61756
ITGB1	NM_033666.2	0	0.127123	9.265407	0.45692	0.64988	0.967393	-4.6178
CD68	NM_001251.2	0	-0.14099	9.592528	-0.4528	0.652826	0.967393	-4.61791
OAS3	NM_006187.2	0.024	0.36165	5.89985	0.442425	0.660257	0.967393	-4.61817
HLA-C	NM_002117.4	0	-0.11438	12.5779	-0.43943	0.66241	0.967393	-4.61824
PAX5	NM_016734.1	0.178	0.318128	3.936745	0.437596	0.663729	0.967393	-4.61829
FCER1A	NM_002001.2	0.12	0.433732	4.747797	0.434108	0.666241	0.967393	-4.61838
CXCL1	NM_001511.1	0.144	0.408447	5.006703	0.431942	0.667803	0.967393	-4.61843
IGF2R	NM_000876.1	0.003	-0.14573	7.211368	-0.42513	0.672727	0.967393	-4.61859
GZMB	NM_004131.3	0.003	0.269532	8.562487	0.412257	0.682067	0.967393	-4.6189

EWSR1	NM_013986.3	0	-0.07634	8.259702	-0.40882	0.684567	0.967393	-4.61898
ENG	NM_001114753.1	0.051	-0.28662	4.250448	-0.40719	0.685757	0.967393	-4.61901
CD6	NM_006725.3	0.014	0.246242	6.224248	0.40426	0.687896	0.967393	-4.61908
KIR_Activating_Subgroup_1	NM_001083539.1	0.469	-0.3743	1.765709	-0.40275	0.689002	0.967393	-4.61912
IKBKE	NM_014002.2	0.027	0.250712	5.332283	0.401849	0.689657	0.967393	-4.61914
IL7R	NM_002185.2	0.01	-0.26854	7.403287	-0.40152	0.689897	0.967393	-4.61914
TNFRSF13B	NM_012452.2	0.418	-0.32932	1.560851	-0.4014	0.689987	0.967393	-4.61915
IL18R1	NM_003855.2	0.106	-0.34549	4.143967	-0.40069	0.690504	0.967393	-4.61916
CCL3	NM_002983.2	0	-0.15976	9.132729	-0.39926	0.691548	0.967393	-4.61919
PSMB7	NM_002799.2	0	0.095609	8.640348	0.399196	0.691598	0.967393	-4.6192
IFIT2	NM_001547.4	0.003	-0.31851	7.040508	-0.39819	0.692331	0.967393	-4.61922
LTA	NM_000595.2	0.017	-0.2547	5.278901	-0.3954	0.694375	0.967393	-4.61928
CR1	NM_000651.4	0	0.205025	8.329865	0.394	0.695403	0.967393	-4.61931
ILF3	NM_001137673.1	0	-0.09246	8.590258	-0.38968	0.698576	0.967393	-4.61941
STAT4	NM_003151.2	0.003	0.162436	6.278248	0.388836	0.699193	0.967393	-4.61943
IL15RA	NM_002189.2	0	0.160847	5.63609	0.386611	0.700828	0.967393	-4.61947
CXCR3	NM_001504.1	0.11	-0.28016	3.352525	-0.38583	0.701401	0.967393	-4.61949
MAPK1	NM_138957.2	0	0.114649	8.055641	0.384541	0.702351	0.967393	-4.61952
ATF2	NM_001256090.1	0	-0.08603	6.400367	-0.38048	0.705341	0.967393	-4.61961
KIR_Inhibiting_Subgroup_2	NM_014511.3	0.466	0.25989	1.661611	0.376738	0.708101	0.967393	-4.61969
CD1D	NM_001766.3	0	0.133765	6.577481	0.373378	0.710583	0.967393	-4.61976
SERPINB2	NM_002575.1	0.075	0.361435	5.698605	0.373074	0.710808	0.967393	-4.61976
ITGAM	NM_000632.3	0	0.133471	8.134644	0.372464	0.711259	0.967393	-4.61978
CARD11	NM_032415.2	0	0.152785	6.802	0.363369	0.717995	0.967393	-4.61997
HLA-DMA	NM_006120.3	0	0.104734	8.465897	0.361499	0.719383	0.967393	-4.62
ABL1	NM_005157.3	0.014	-0.14492	4.655954	-0.35403	0.724937	0.967393	-4.62016
DPP4	NM_001935.3	0.366	-0.29449	1.607852	-0.35324	0.72552	0.967393	-4.62017
IL17RA	NM_014339.6	0.003	-0.13776	7.20004	-0.34789	0.729512	0.967393	-4.62028
CD160	NM_007053.2	0.171	-0.30584	3.628464	-0.34578	0.731086	0.967393	-4.62032
RORA	NM_134261.2	0.003	0.170553	6.734638	0.340303	0.735179	0.967393	-4.62042

CD55	NM_000574.3	0.264	-0.30758	3.129697	-0.33935	0.735893	0.967393	-4.62044
MAP2K1	NM_002755.2	0	0.108515	7.380962	0.339301	0.735928	0.967393	-4.62044
SLAMF6	NM_001184714.1	0.267	0.29427	2.349106	0.33906	0.736109	0.967393	-4.62045
POU2AF1	NM_006235.2	0.455	0.319944	1.810931	0.338995	0.736157	0.967393	-4.62045
TANK	NM_004180.2	0	-0.09819	8.706135	-0.33565	0.738661	0.967393	-4.62051
CXCL5	NM_002994.3	0.027	-0.24468	5.848684	-0.33334	0.740394	0.967393	-4.62056
JAM3	NM_032801.3	0.027	-0.26587	5.043229	-0.33068	0.74239	0.967393	-4.62061
TNFSF12	NM_003809.2	0	-0.13043	7.372529	-0.321	0.749667	0.967393	-4.62079
IFNAR2	NM_000874.3	0	-0.07641	8.322536	-0.3182	0.751771	0.967393	-4.62084
CCR6	NM_031409.2	0.301	-0.25579	1.833526	-0.3154	0.753883	0.967393	-4.62089
TNFSF8	NM_001244.3	0.007	-0.11452	5.552587	-0.31499	0.754193	0.967393	-4.62089
PPBP	NM_002704.2	0	-0.2021	11.92664	-0.31447	0.754584	0.967393	-4.6209
SIGLEC1	NM_023068.3	0.182	-0.38739	3.727891	-0.31301	0.755689	0.967393	-4.62093
KIR_Inhibiting_Subgroup_1	NM_014218.2	0.243	0.220761	2.758276	0.31085	0.75732	0.967393	-4.62097
CTSW	NM_001335.3	0.003	0.159823	7.545881	0.309402	0.758414	0.967393	-4.62099
LAG3	NM_002286.5	0.065	0.258618	4.236761	0.307129	0.760132	0.967393	-4.62103
KIR_Activating_Subgroup_2	NM_014512.1	0.349	-0.25516	2.183157	-0.30643	0.760658	0.967393	-4.62104
FCER1G	NM_004106.1	0	0.121139	10.81787	0.30136	0.764499	0.967393	-4.62113
PRKCD	NM_006254.3	0	-0.10259	8.930837	-0.299	0.766284	0.967393	-4.62117
CD33	NM_001177608.1	0.024	0.174417	5.292569	0.29865	0.766553	0.967393	-4.62118
CMKLR1	NM_004072.1	0.301	-0.30325	2.694735	-0.2986	0.766588	0.967393	-4.62118
CCR1	NM_001295.2	0.007	0.167999	6.124604	0.295516	0.768931	0.967393	-4.62123
NT5E	NM_002526.2	0.137	-0.24341	2.662419	-0.29303	0.770815	0.967393	-4.62127
MAP2K4	NM_003010.2	0.171	-0.24983	2.984437	-0.2929	0.77092	0.967393	-4.62127
CHUK	NM_001278.3	0	0.107873	6.830635	0.292442	0.771265	0.967393	-4.62128
RUNX1	NM_001754.4	0	-0.09456	6.466619	-0.29197	0.771625	0.967393	-4.62129
PTPRC	NM_080921.3	0	0.117816	9.185927	0.291099	0.772285	0.967393	-4.6213
NCR1	NM_004829.5	0.017	0.158447	5.448008	0.290052	0.773081	0.967393	-4.62132
TAPBP	NM_003190.4	0	-0.07422	9.897147	-0.28275	0.778638	0.967393	-4.62144
HLA-DMB	NM_002118.3	0	0.108724	8.249254	0.281031	0.779948	0.967393	-4.62147

CD8A	NM_001768.5	0	0.153085	7.690719	0.275703	0.784012	0.967393	-4.62155
CD83	NM_004233.3	0	-0.10978	10.37966	-0.27319	0.785929	0.967393	-4.62159
CD3E	NM_000733.2	0.003	0.174586	7.823806	0.273186	0.785934	0.967393	-4.62159
LAMP2	NM_001122606.1	0	-0.06591	7.404305	-0.27309	0.786005	0.967393	-4.62159
IFITM1	NM_003641.3	0	0.123775	10.37725	0.271781	0.787007	0.967393	-4.62161
TBK1	NM_013254.2	0	0.073406	6.159074	0.2715	0.787222	0.967393	-4.62161
TLR6	NM_006068.2	0.024	-0.12093	5.028522	-0.27083	0.787734	0.967393	-4.62162
CTSH	NM_004390.3	0	0.09014	8.71091	0.266343	0.791166	0.969179	-4.62169
PECAM1	NM_000442.3	0	0.064745	9.131581	0.263355	0.793453	0.969557	-4.62174
NOD2	NM_022162.1	0	-0.08608	6.654154	-0.25182	0.802299	0.970836	-4.62191
MAPK8	NM_002750.2	0.017	-0.12358	4.369522	-0.24998	0.80372	0.970836	-4.62193
TNFSF13B	NM_006573.4	0.003	0.152341	6.395955	0.248597	0.80478	0.970836	-4.62195
REPS1	NM_001128617.2	0	0.07369	7.156387	0.248458	0.804887	0.970836	-4.62195
IFIH1	NM_022168.2	0.003	-0.1056	6.32183	-0.24674	0.806211	0.970836	-4.62198
DOCK9	NM_001130048.1	0.024	-0.10078	4.890024	-0.24651	0.806388	0.970836	-4.62198
FYN	NM_002037.3	0	-0.09802	8.416811	-0.24187	0.809958	0.972743	-4.62205
CFP	NM_002621.2	0	-0.07898	8.814278	-0.23825	0.812743	0.973702	-4.62209
CD53	NM_001040033.1	0	0.059065	10.19694	0.22921	0.819723	0.975681	-4.62221
LTF	NM_002343.2	0.075	-0.23615	5.834957	-0.22627	0.821995	0.975681	-4.62225
FLT3LG	NM_001459.3	0	0.075226	7.123105	0.219574	0.827175	0.975681	-4.62234
ITCH	NM_001257138.1	0	-0.05148	8.144915	-0.21839	0.828092	0.975681	-4.62235
ITGA4	NM_000885.4	0	-0.05202	7.618351	-0.21817	0.828263	0.975681	-4.62235
APP	NM_000484.3	0	0.060948	7.831006	0.217769	0.828573	0.975681	-4.62236
FUT7	NM_004479.3	0.058	-0.16615	5.104936	-0.21739	0.828867	0.975681	-4.62236
CD96	NM_005816.4	0.007	0.127162	7.013041	0.212177	0.832907	0.975681	-4.62243
IRF1	NM_002198.1	0	0.097373	10.24649	0.208115	0.836058	0.975681	-4.62248
CD7	NM_006137.6	0.007	0.11275	6.681639	0.206931	0.836978	0.975681	-4.62249
C1QB	NM_000491.3	0.195	0.181173	3.174431	0.206233	0.83752	0.975681	-4.6225
FCGR3A	NM_000569.6	0	-0.10783	8.95422	-0.20128	0.841369	0.975681	-4.62255
TAB1	NM_153497.2	0.027	-0.13239	4.629758	-0.19963	0.842648	0.975681	-4.62257

CCR3	NM_001837.2	0.455	-0.18313	2.209823	-0.19755	0.844271	0.975681	-4.6226
TLR10	NM_030956.2	0.096	0.149076	3.445633	0.194534	0.846615	0.975681	-4.62263
IKBKB	NM_001556.1	0.024	-0.08512	4.93791	-0.19404	0.846999	0.975681	-4.62264
SIGIRR	NM_021805.2	0.007	0.09702	6.474541	0.19244	0.848245	0.975681	-4.62265
CREB5	NM_182898.2	0.007	-0.09888	6.674693	-0.18958	0.850475	0.975955	-4.62268
IRAK4	NM_016123.1	0.007	0.072142	5.505175	0.179233	0.858542	0.976271	-4.62279
PTGS2	NM_000963.1	0.034	0.161169	6.999915	0.172517	0.863788	0.976271	-4.62286
IFI16	NM_005531.1	0	0.059532	6.92399	0.170045	0.865721	0.976271	-4.62288
IL1B	NM_000576.2	0.031	-0.15845	7.188556	-0.16856	0.866886	0.976271	-4.6229
LCN2	NM_005564.3	0.007	0.133389	6.478134	0.167366	0.867816	0.976271	-4.62291
MSR1	NM_002445.3	0.082	-0.14595	3.928942	-0.16716	0.867979	0.976271	-4.62291
CD8B	NM_004931.3	0.024	0.108528	5.719368	0.16709	0.868032	0.976271	-4.62291
IL3RA	NM_002183.2	0.161	-0.1171	3.213654	-0.16486	0.869773	0.976271	-4.62293
MICB	NM_005931.3	0	0.036116	6.836105	0.163945	0.870493	0.976271	-4.62294
ZAP70	NM_001079.3	0.003	0.094757	7.326226	0.161684	0.872264	0.976271	-4.62296
CASP3	NM_032991.2	0.007	-0.05896	5.089648	-0.15388	0.878378	0.976271	-4.62303
MAP3K7	NM_145333.1	0	-0.04317	6.660926	-0.15047	0.881053	0.976271	-4.62306
IL16	NM_004513.4	0	0.030216	7.747595	0.148558	0.882552	0.976271	-4.62308
TNFSF4	NM_003326.2	0.007	-0.09259	5.133709	-0.14847	0.882625	0.976271	-4.62308
TAP1	NM_000593.5	0	-0.04654	7.545978	-0.14665	0.884053	0.976271	-4.62309
CD14	NM_000591.2	0	0.06416	9.172324	0.14629	0.884332	0.976271	-4.6231
CD5	NM_014207.2	0.007	0.082539	7.011149	0.144423	0.885798	0.976271	-4.62311
HLA-DQB1	NM_002123.3	0.315	0.176266	5.047712	0.139809	0.889422	0.976271	-4.62315
TCF7	NM_003202.2	0.021	0.088829	6.956808	0.138758	0.890247	0.976271	-4.62316
KLRK1	NM_007360.3	0.007	-0.08649	8.024475	-0.13831	0.890599	0.976271	-4.62316
CLEC4A	NM_194448.2	0	-0.04192	8.014145	-0.13423	0.893805	0.977381	-4.62319
ITGB2	NM_000211.2	0	-0.04401	10.7826	-0.13195	0.895601	0.977381	-4.62321
PRF1	NM_005041.3	0.007	0.095612	8.41812	0.129031	0.897896	0.977709	-4.62323
CD9	NM_001769.2	0	0.059064	6.293884	0.124235	0.901671	0.979642	-4.62327
ICAM3	NM_002162.3	0	0.026997	9.41513	0.120071	0.90495	0.98103	-4.62329

LY96	NM_015364.2	0	-0.05175	8.017329	-0.11296	0.910551	0.984923	-4.62334
SYK	NM_003177.3	0	-0.04133	7.852431	-0.10375	0.917821	0.986441	-4.6234
TYK2	NM_003331.3	0	-0.02724	6.846544	-0.10359	0.917942	0.986441	-4.6234
NOD1	NM_006092.1	0.082	0.060016	3.821186	0.10105	0.91995	0.986441	-4.62341
CD99	NM_002414.3	0	-0.03714	9.897383	-0.1004	0.920466	0.986441	-4.62342
LCP1	NM_002298.4	0	0.029029	11.29639	0.097211	0.922982	0.986441	-4.62344
CD40LG	NM_000074.2	0.082	0.074072	3.902024	0.095376	0.924431	0.986441	-4.62345
IL6	NM_000600.1	0.027	0.056079	5.404137	0.092353	0.926819	0.986441	-4.62346
PYCARD	NM_013258.3	0	-0.03132	7.012295	-0.09035	0.9284	0.986441	-4.62347
PDGFRB	NM_002609.3	0.349	-0.05768	2.205225	-0.0862	0.931683	0.986441	-4.62349
MARCO	NM_006770.3	0.031	0.066695	5.389415	0.083793	0.933585	0.986441	-4.6235
CD44	NM_001001392.1	0	0.028323	11.23368	0.083142	0.934099	0.986441	-4.62351
IFI27	NM_005532.3	0.202	-0.12779	4.230699	-0.07516	0.940417	0.990977	-4.62354
TOLLIP	NM_019009.2	0.01	-0.02107	5.238364	-0.06926	0.945085	0.991769	-4.62357
CTSG	NM_001911.2	0.462	-0.0673	1.970743	-0.06909	0.945217	0.991769	-4.62357
GTF3C1	NM_001520.3	0.007	0.016541	5.587329	0.063342	0.949769	0.992338	-4.62359
TAL1	NM_003189.2	0.058	0.050823	4.597146	0.062782	0.950213	0.992338	-4.62359
HLA-DOB	NM_002120.3	0.01	0.026431	5.360608	0.054653	0.956652	0.992338	-4.62362
CCL3L1	NM_021006.4	0	-0.0225	9.002363	-0.05351	0.957559	0.992338	-4.62362
GNLY	NM_006433.2	0.003	-0.03645	9.761048	-0.04831	0.96168	0.992338	-4.62364
IRF2	NM_002199.3	0	0.019134	6.138062	0.046619	0.963019	0.992338	-4.62364
IL2RB	NM_000878.2	0.014	0.030459	6.905447	0.045547	0.963869	0.992338	-4.62364
ITK	NM_005546.3	0.007	-0.02542	7.125809	-0.04534	0.964033	0.992338	-4.62365
CD1C	NM_001765.2	0.113	0.035226	4.171535	0.043084	0.965821	0.992338	-4.62365
CD247	NM_198053.1	0.007	-0.02427	7.865711	-0.04026	0.968062	0.992338	-4.62366
IRAK2	NM_001570.3	0.021	-0.02428	4.840822	-0.04002	0.968249	0.992338	-4.62366
TLR8	NM_016610.2	0.014	-0.01725	5.419839	-0.03774	0.970061	0.992338	-4.62366
JAK3	NM_000215.2	0	-0.00995	7.815328	-0.02943	0.976648	0.994033	-4.62368
BST1	NM_004334.2	0	0.011095	8.513588	0.029293	0.976758	0.994033	-4.62368
CCL5	NM_002985.2	0	0.014414	10.64813	0.027974	0.977804	0.994033	-4.62368

ATG5	NM_004849.2	0	0.007738	6.140491	0.024319	0.980704	0.994917	-4.62369
SOCS1	NM_003745.1	0.024	0.011155	5.50065	0.01948	0.984543	0.99645	-4.62369
SERPING1	NM_000062.2	0.024	-0.01565	5.143837	-0.01729	0.986282	0.99645	-4.6237
MAPK14	NM_001315.1	0	0.005991	8.403621	0.014464	0.988522	0.996658	-4.6237
NLRC5	NM_032206.4	0.003	0.003522	7.111103	0.008708	0.99309	0.997255	-4.6237
JAK2	NM_004972.2	0	0.00157	8.03719	0.005638	0.995526	0.997255	-4.6237
CD163	NM_004244.4	0.007	-0.00291	7.068783	-0.00495	0.996075	0.997255	-4.6237
NFKB1	NM_003998.2	0.003	-0.00128	5.894697	-0.00346	0.997255	0.997255	-4.6237
**D. Rd at C3D1 versus C1D1, CyTOF corrected**								
IL11RA	NM_147162.1	0.161	1.798013	3.286179	3.426965	0.001296	0.635194	-4.20086
IL10	NM_000572.2	0.253	1.483469	3.047345	2.519011	0.01531	0.985398	-4.38054
CARD11	NM_032415.2	0	0.804981	6.802	2.507854	0.015741	0.985398	-4.3826
C2	NM_000063.3	0.243	1.695595	2.476793	2.414457	0.019797	0.985398	-4.39959
KIR_Activating_Subgroup_2	NM_014512.1	0.349	1.531017	2.183157	2.408508	0.020084	0.985398	-4.40066
MERTK	NM_006343.2	0.171	1.555945	2.777205	2.387631	0.021125	0.985398	-4.4044
IL3RA	NM_002183.2	0.161	1.281985	3.213654	2.364291	0.022345	0.985398	-4.40857
CD40	NM_001250.4	0.021	0.930806	5.185361	2.291576	0.026561	0.985398	-4.42138
STAT4	NM_003151.2	0.003	0.696408	6.278248	2.183726	0.034123	0.985398	-4.43993
FCGR1A	NM_000566.3	0.202	1.320929	2.641645	2.173499	0.034931	0.985398	-4.44166
BLK	NM_001715.2	0.12	1.162324	3.613267	2.126549	0.038858	0.985398	-4.44954
TNFSF13	NM_003808.3	0	0.571923	6.751035	2.083547	0.042789	0.985398	-4.45665
S100B	NM_006272.1	0.5	1.23405	2.099881	2.069662	0.044131	0.985398	-4.45893
IL1RN	NM_000577.3	0.003	-1.22649	7.831008	-2.06118	0.044968	0.985398	-4.46031
BMI1	NM_005180.5	0	-0.62793	8.109578	-2.03535	0.047604	0.985398	-4.46451
CYBB	NM_000397.3	0	0.517487	9.618004	2.014234	0.049858	0.985398	-4.46791
PAX5	NM_016734.1	0.178	1.103911	3.936745	1.989094	0.052661	0.985398	-4.47193
STAT1	NM_007315.2	0	0.547141	8.931527	1.968533	0.055054	0.985398	-4.47519
KLRC2	NM_002260.3	0.134	1.204215	4.132589	1.956614	0.056483	0.985398	-4.47707
MSR1	NM_002445.3	0.082	1.280898	3.928942	1.921769	0.060846	0.985398	-4.48251
NCR1	NM_004829.5	0.017	0.794154	5.448008	1.904353	0.063133	0.985398	-4.48521

C1QB	NM_000491.3	0.195	1.266757	3.174431	1.888899	0.065224	0.985398	-4.48759
KIR_Inhibiting_Subgroup_1	NM_014218.2	0.243	1.023307	2.758276	1.88749	0.065417	0.985398	-4.4878
CD4	NM_000616.4	0	0.457854	7.707342	1.876581	0.066932	0.985398	-4.48947
MS4A1	NM_152866.2	0.051	1.085691	5.957998	1.863087	0.068846	0.985398	-4.49153
ADORA2A	NM_000675.3	0.003	-0.97561	6.821263	-1.85864	0.069487	0.985398	-4.4922
HLA-DRB3	NM_022555.3	0	0.465345	11.11413	1.857576	0.069642	0.985398	-4.49237
FOXJ1	NM_001454.3	0	-0.62397	5.445389	-1.84075	0.072117	0.985398	-4.49491
IRF8	NM_002163.2	0	0.448319	7.368256	1.801297	0.078217	0.985398	-4.5008
CD22	NM_001771.2	0.068	1.034121	5.510924	1.79968	0.078476	0.985398	-4.50104
HLA-DOB	NM_002120.3	0.01	0.655737	5.360608	1.77614	0.082329	0.985398	-4.50451
LAIR2	NM_002288.3	0.014	0.729444	4.742557	1.762494	0.084634	0.985398	-4.5065
IL32	NM_001012633.1	0	-0.5347	11.05665	-1.74021	0.088513	0.985398	-4.50973
ELK1	NM_005229.3	0.031	-0.66941	4.410011	-1.72591	0.09108	0.985398	-4.51179
CD33	NM_001177608.1	0.024	0.768086	5.292569	1.722795	0.091647	0.985398	-4.51224
IRF3	NM_001571.5	0.01	-0.58442	5.238257	-1.72265	0.091674	0.985398	-4.51226
HLA-DMA	NM_006120.3	0	0.380097	8.465897	1.718564	0.092423	0.985398	-4.51284
PRKCD	NM_006254.3	0	-0.44599	8.930837	-1.70272	0.095374	0.985398	-4.5151
TNFRSF13B	NM_012452.2	0.418	1.038179	1.560851	1.657593	0.10421	0.985398	-4.52144
TNFRSF13C	NM_052945.3	0.045	0.78931	5.933964	1.605358	0.115264	0.985398	-4.52861
HLA-DRA	NM_019111.3	0	0.362028	12.4152	1.603477	0.115679	0.985398	-4.52886
NLRC5	NM_032206.4	0.003	0.493656	7.111103	1.598959	0.116681	0.985398	-4.52947
LTF	NM_002343.2	0.075	-1.2637	5.834957	-1.5861	0.119573	0.985398	-4.53121
LAMP2	NM_001122606.1	0	-0.29186	7.404305	-1.58413	0.120019	0.985398	-4.53147
NFKBIA	NM_020529.1	0	-0.57049	13.82902	-1.54487	0.129235	0.985398	-4.53668
REPS1	NM_001128617.2	0	-0.34728	7.156387	-1.53382	0.131927	0.985398	-4.53813
CXCR2	NM_001557.2	0.058	-0.92439	6.942205	-1.52391	0.134381	0.985398	-4.53942
TLR5	NM_003268.3	0.202	0.994227	3.397969	1.519796	0.135411	0.985398	-4.53995
PSMB10	NM_002801.2	0	0.26136	8.863405	1.51684	0.136154	0.985398	-4.54034
LTBR	NM_002342.1	0.007	0.545352	6.427198	1.508733	0.13821	0.985398	-4.54138
NFKB2	NM_002502.2	0	-0.55114	9.750106	-1.50358	0.13953	0.985398	-4.54205

KLRC1	NM_002259.3	0.079	0.841469	4.543482	1.498593	0.140816	0.985398	-4.54269
KLRF1	NM_016523.1	0.086	0.870079	5.565089	1.493443	0.142154	0.985398	-4.54335
CTSS	NM_004079.3	0	0.289409	12.63254	1.489592	0.143161	0.985398	-4.54384
PVR	NM_006505.3	0.182	0.887667	2.690121	1.479458	0.145839	0.985398	-4.54513
CXCR3	NM_001504.1	0.11	0.819749	3.352525	1.478869	0.145996	0.985398	-4.5452
EGR1	NM_001964.2	0.031	-0.99761	6.651427	-1.47817	0.146183	0.985398	-4.54529
TRAF3	NM_145725.1	0	-0.31618	7.125145	-1.47816	0.146185	0.985398	-4.54529
TOLLIP	NM_019009.2	0.01	0.342092	5.238364	1.472722	0.14764	0.985398	-4.54598
CCL4	NM_002984.2	0	-0.5874	7.54716	-1.46713	0.149148	0.985398	-4.54669
HLA-DPA1	NM_033554.2	0	0.36533	10.19163	1.456581	0.152029	0.985398	-4.54801
CD59	NM_000611.4	0	-0.57434	6.155392	-1.45314	0.152978	0.985398	-4.54844
SH2D1B	NM_053282.4	0.205	0.930876	3.51781	1.446626	0.154786	0.985398	-4.54925
CTSH	NM_004390.3	0	0.373184	8.71091	1.444433	0.155399	0.985398	-4.54953
CD180	NM_005582.2	0.164	1.0012	2.984111	1.442999	0.155801	0.985398	-4.5497
TNFRSF10B	NM_003842.3	0.014	-0.46774	5.755992	-1.44225	0.15601	0.985398	-4.5498
NOD1	NM_006092.1	0.082	0.644113	3.821186	1.42064	0.162169	0.985398	-4.55246
IL12RB1	NM_005535.1	0	0.316186	5.5927	1.407719	0.165941	0.985398	-4.55404
CD163	NM_004244.4	0.007	0.631753	7.068783	1.406793	0.166214	0.985398	-4.55416
PSEN2	NM_000447.2	0.058	0.649076	3.563695	1.406118	0.166413	0.985398	-4.55424
CD74	NM_001025159.1	0	0.276114	12.54212	1.38783	0.171879	0.985398	-4.55645
GNLY	NM_006433.2	0.003	0.798564	9.761048	1.386571	0.172261	0.985398	-4.5566
PNMA1	NM_006029.4	0	-0.42501	5.394446	-1.38529	0.17265	0.985398	-4.55675
CD1C	NM_001765.2	0.113	0.860914	4.171535	1.379317	0.174471	0.985398	-4.55747
CD79A	NM_001783.3	0.003	0.521337	7.370404	1.36947	0.177506	0.985398	-4.55864
ATF1	NM_005171.2	0.007	0.484885	5.417584	1.366297	0.178492	0.985398	-4.55902
CD68	NM_001251.2	0	0.324063	9.592528	1.363337	0.179416	0.985398	-4.55937
MAP3K7	NM_145333.1	0	-0.29844	6.660926	-1.36269	0.179618	0.985398	-4.55945
HLA-DPB1	NM_002121.4	0	0.322107	10.20357	1.351574	0.183125	0.985398	-4.56076
SH2B2	NM_020979.3	0	-0.40601	6.308864	-1.34794	0.184283	0.985398	-4.56119
POU2AF1	NM_006235.2	0.455	0.967175	1.810931	1.342375	0.186066	0.985398	-4.56184

CD14	NM_000591.2	0	0.443142	9.172324	1.32356	0.192194	0.985398	-4.56402
TNFRSF18	NM_004195.2	0.247	0.781296	2.462623	1.322609	0.192508	0.985398	-4.56413
FCER2	NM_002002.4	0.082	0.735942	4.838727	1.320402	0.193238	0.985398	-4.56439
MAPKAPK2	NM_004759.3	0	-0.35918	8.36488	-1.31788	0.194073	0.985398	-4.56468
TRAF6	NM_145803.1	0	-0.30852	6.417511	-1.314	0.195365	0.985398	-4.56512
CD1D	NM_001766.3	0	0.354061	6.577481	1.2946	0.201924	0.985398	-4.56733
LCN2	NM_005564.3	0.007	-0.78658	6.478134	-1.29283	0.202531	0.985398	-4.56754
CLEC7A	NM_197954.2	0	-0.4377	8.867019	-1.28908	0.203818	0.985398	-4.56796
IL15RA	NM_002189.2	0	0.408764	5.63609	1.287018	0.204531	0.985398	-4.56819
PRF1	NM_005041.3	0.007	0.725785	8.41812	1.283039	0.205909	0.985398	-4.56864
HLA-DRB4	NM_021983.4	0.586	1.651185	3.214603	1.270467	0.210311	0.985398	-4.57005
BLNK	NM_013314.2	0.432	0.655209	1.742082	1.268962	0.210842	0.985398	-4.57021
CASP1	NM_001223.3	0	0.347511	8.0139	1.259388	0.214247	0.985398	-4.57128
LY96	NM_015364.2	0	-0.43926	8.017329	-1.25605	0.215444	0.985398	-4.57164
CTSL	NM_001912.4	0.205	0.983165	2.654839	1.246762	0.2188	0.985398	-4.57267
CFP	NM_002621.2	0	0.312735	8.814278	1.235802	0.22281	0.985398	-4.57386
ZAP70	NM_001079.3	0.003	0.5486	7.326226	1.226198	0.226369	0.985398	-4.5749
RELA	NM_021975.2	0	-0.24878	6.773554	-1.22524	0.226725	0.985398	-4.57501
PTGS2	NM_000963.1	0.034	-0.87299	6.999915	-1.22409	0.227156	0.985398	-4.57513
TNFRSF9	NM_001561.4	0.329	0.632482	1.979191	1.220433	0.228524	0.985398	-4.57553
HLA-DMB	NM_002118.3	0	0.35994	8.249254	1.218738	0.229161	0.985398	-4.57571
NCF4	NM_000631.4	0	-0.40764	7.708288	-1.2176	0.229589	0.985398	-4.57583
IFITM2	NM_006435.2	0	-0.43515	11.276	-1.21664	0.229951	0.985398	-4.57593
CSF1R	NM_005211.2	0.164	0.919738	3.562819	1.214371	0.230808	0.985398	-4.57618
KLRD1	NM_002262.3	0.014	0.67276	6.740085	1.190213	0.240073	0.985398	-4.57874
CD160	NM_007053.2	0.171	0.801324	3.628464	1.186773	0.241414	0.985398	-4.5791
MICB	NM_005931.3	0	-0.19932	6.836105	-1.18523	0.242016	0.985398	-4.57926
IKBKE	NM_014002.2	0.027	0.56062	5.332283	1.177081	0.245222	0.985398	-4.58012
IFNAR2	NM_000874.3	0	-0.21461	8.322536	-1.17068	0.24776	0.985398	-4.58078
CCR7	NM_001838.2	0.113	0.65721	4.026042	1.16605	0.249608	0.985398	-4.58126

CCR2	NM_001123041.2	0.175	0.808327	3.386746	1.152702	0.254992	0.985398	-4.58263
CD24	NM_013230.2	0.007	-0.58048	5.739555	-1.15127	0.255576	0.985398	-4.58278
CD47	NM_001777.3	0	-0.15767	8.848972	-1.14759	0.257077	0.985398	-4.58316
NCAM1	NM_000615.5	0.017	0.474546	4.399241	1.142898	0.258998	0.985398	-4.58363
SIGIRR	NM_021805.2	0.007	0.439093	6.474541	1.140886	0.259826	0.985398	-4.58384
CCR1	NM_001295.2	0.007	0.494412	6.124604	1.139238	0.260506	0.985398	-4.584
CD40LG	NM_000074.2	0.082	-0.67291	3.902024	-1.13498	0.262266	0.985398	-4.58443
S100A12	NM_005621.1	0	0.401002	10.56909	1.13164	0.263655	0.985398	-4.58477
PRKCE	NM_005400.2	0.151	0.683743	2.835834	1.125524	0.266209	0.985398	-4.58538
SPN	NM_003123.3	0.017	0.501899	5.348767	1.112924	0.271527	0.985398	-4.58664
INPP5D	NM_005541.3	0	0.23806	7.652128	1.102739	0.27588	0.985398	-4.58764
TXNIP	NM_006472.1	0	-0.25585	12.34162	-1.10197	0.27621	0.985398	-4.58772
CD63	NM_001780.4	0	-0.26926	9.814142	-1.10019	0.276979	0.985398	-4.5879
C1QA	NM_015991.2	0.127	0.67106	4.005041	1.088955	0.281849	0.985398	-4.58899
LTA	NM_000595.2	0.017	0.533619	5.278901	1.085169	0.283505	0.985398	-4.58936
FCER1G	NM_004106.1	0	0.331335	10.81787	1.079744	0.285889	0.985398	-4.58988
PLAU	NM_002658.2	0.226	-1.16833	4.97886	-1.07792	0.286695	0.985398	-4.59006
MR1	NM_001531.2	0.062	-0.52992	3.474107	-1.06673	0.291666	0.985398	-4.59113
TLR1	NM_003263.3	0	-0.28981	7.71714	-1.06282	0.293416	0.985398	-4.5915
GZMK	NM_002104.2	0.01	0.523896	6.677627	1.056708	0.296167	0.985398	-4.59208
LILRB2	NM_005874.1	0	0.306655	7.717561	1.056332	0.296336	0.985398	-4.59212
ATG16L1	NM_198890.2	0.007	0.301853	5.810312	1.055864	0.296548	0.985398	-4.59216
TREM1	NM_018643.3	0	-0.50133	10.27873	-1.05582	0.296569	0.985398	-4.59217
MAP3K1	NM_005921.1	0	-0.18715	8.05806	-1.03953	0.303997	0.985398	-4.5937
IL10RA	NM_001558.2	0	0.211834	9.547293	1.034994	0.306086	0.985398	-4.59412
IRF5	NM_002200.3	0	0.239199	6.435055	1.021321	0.312447	0.985398	-4.59538
ITGB2	NM_000211.2	0	0.257523	10.7826	1.011482	0.31708	0.985398	-4.59628
IL1B	NM_000576.2	0.031	-0.7256	7.188556	-1.01111	0.317255	0.985398	-4.59631
BTK	NM_000061.1	0	0.18201	6.784176	1.006716	0.319341	0.985398	-4.59671
TICAM2	NM_021649.4	0.158	0.67014	2.653328	1.003573	0.320838	0.985398	-4.59699

GZMB	NM_004131.3	0.003	0.500539	8.562487	1.002875	0.321171	0.985398	-4.59706
TAP1	NM_000593.5	0	-0.24175	7.545978	-0.99779	0.323606	0.985398	-4.59751
CTSW	NM_001335.3	0.003	0.391668	7.545881	0.993234	0.325796	0.985398	-4.59792
LY86	NM_004271.3	0	0.263605	7.747927	0.986621	0.328993	0.985398	-4.59851
CCR6	NM_031409.2	0.301	0.609659	1.833526	0.984735	0.329909	0.985398	-4.59868
RUNX1	NM_001754.4	0	0.243288	6.466619	0.984052	0.330241	0.985398	-4.59874
CD36	NM_001001548.2	0	0.228364	9.11305	0.982701	0.330898	0.985398	-4.59886
TFEB	NM_007162.2	0.014	0.373534	5.550239	0.980875	0.331789	0.985398	-4.59902
IFIT2	NM_001547.4	0.003	0.595959	7.040508	0.975957	0.334194	0.985398	-4.59945
CARD9	NM_052813.4	0.089	0.498482	4.147282	0.974736	0.334793	0.985398	-4.59956
KLRG1	NM_005810.3	0.051	0.483053	5.462645	0.973964	0.335172	0.985398	-4.59963
CDKN1A	NM_000389.2	0	0.237441	9.021987	0.972699	0.335794	0.985398	-4.59974
CD38	NM_001775.2	0.007	0.518244	5.538861	0.972497	0.335893	0.985398	-4.59976
MIF	NM_002415.1	0	-0.24117	8.051564	-0.96878	0.337725	0.985398	-4.60008
CSF3R	NM_156038.2	0	-0.41973	10.61811	-0.96598	0.33911	0.985398	-4.60033
PSMB9	NM_002800.4	0	0.267738	8.72407	0.963997	0.340091	0.985398	-4.6005
MAF	NM_005360.4	0.021	0.50638	5.119079	0.963031	0.340571	0.985398	-4.60058
BCL10	NM_003921.2	0	-0.18342	8.084753	-0.96174	0.341211	0.985398	-4.60069
CD244	NM_016382.2	0.017	0.463828	5.927828	0.959805	0.342174	0.985398	-4.60086
IGF1R	NM_000875.2	0	-0.36227	6.662979	-0.95651	0.343819	0.985398	-4.60115
CCR5	NM_000579.1	0.462	0.577957	1.470938	0.953434	0.345357	0.985398	-4.60141
MAPK14	NM_001315.1	0	-0.30137	8.403621	-0.95319	0.34548	0.985398	-4.60143
TBX21	NM_013351.1	0.007	0.389253	6.903745	0.946586	0.348799	0.985398	-4.602
BCL2	NM_000657.2	0	0.257587	6.051298	0.945012	0.349593	0.985398	-4.60213
CXCR4	NM_003467.2	0	-0.2472	11.71915	-0.94296	0.350633	0.985398	-4.60231
KIR_Inhibiting_Subgroup_2	NM_014511.3	0.466	0.492236	1.661611	0.934703	0.354826	0.985398	-4.60301
SYK	NM_003177.3	0	0.284192	7.852431	0.934569	0.354894	0.985398	-4.60302
ABCB1	NM_000927.3	0.092	0.39259	4.00911	0.930747	0.356847	0.985398	-4.60334
IL1RAP	NM_002182.2	0	-0.39401	6.309927	-0.92171	0.361491	0.985398	-4.6041
CD79B	NM_021602.2	0	0.350988	6.988636	0.921661	0.361517	0.985398	-4.6041

TNFRSF10C	NM_003841.3	0.106	-0.62941	5.932921	-0.91866	0.363068	0.985398	-4.60435
PSEN1	NM_000021.2	0	0.178264	6.561164	0.917105	0.363874	0.985398	-4.60448
IL8	NM_000584.2	0	-0.45061	13.63443	-0.91	0.36757	0.985398	-4.60507
RIPK2	NM_003821.5	0	0.280051	8.631872	0.90963	0.367763	0.985398	-4.6051
TNFRSF14	NM_003820.2	0	0.154836	7.563062	0.906023	0.369648	0.985398	-4.6054
MX1	NM_002462.2	0	-0.38922	7.86973	-0.90518	0.370089	0.985398	-4.60547
ENTPD1	NM_001098175.1	0.092	0.500612	4.032419	0.902925	0.371273	0.985398	-4.60565
TGFB1	NM_000660.3	0	-0.22283	10.97278	-0.89472	0.375599	0.985398	-4.60632
HLA-C	NM_002117.4	0	-0.17748	12.5779	-0.89321	0.376397	0.985398	-4.60644
HLA-E	NM_005516.4	0	-0.16399	11.62095	-0.89042	0.377879	0.985398	-4.60667
NUP107	NM_020401.2	0.003	-0.18521	5.270172	-0.89022	0.377981	0.985398	-4.60668
KIR_Activating_Subgroup_1	NM_001083539.1	0.469	0.623683	1.765709	0.879066	0.383937	0.985398	-4.60758
TAPBP	NM_003190.4	0	-0.17612	9.897147	-0.8789	0.384027	0.985398	-4.60759
IL16	NM_004513.4	0	-0.13646	7.747595	-0.87885	0.384053	0.985398	-4.6076
CD9	NM_001769.2	0	-0.31866	6.293884	-0.87801	0.384504	0.985398	-4.60766
CD7	NM_006137.6	0.007	0.361263	6.681639	0.868525	0.389619	0.985398	-4.60842
ITGAM	NM_000632.3	0	0.237039	8.134644	0.8665	0.390716	0.985398	-4.60858
UBC	NM_021009.3	0	-0.18745	14.13579	-0.86254	0.392867	0.985398	-4.60889
AKT3	NM_181690.1	0.003	-0.26645	6.175368	-0.85623	0.39631	0.985398	-4.60938
MAP2K4	NM_003010.2	0.171	0.557116	2.984437	0.855578	0.396669	0.985398	-4.60943
DUSP4	NM_057158.2	0.164	0.634357	3.60756	0.852835	0.398173	0.985398	-4.60964
CD274	NM_014143.3	0.469	0.561323	1.426443	0.852155	0.398546	0.985398	-4.6097
IRAK2	NM_001570.3	0.021	-0.39375	4.840822	-0.85012	0.399665	0.985398	-4.60985
RPS6	NM_001010.2	0	-0.20137	12.83735	-0.84759	0.401058	0.985398	-4.61005
ITCH	NM_001257138.1	0	-0.15246	8.144915	-0.84724	0.401249	0.985398	-4.61008
HLA-A	NM_002116.5	0	-0.15709	13.41276	-0.84539	0.40227	0.985398	-4.61022
ECSIT	NM_001142464.2	0.003	-0.28683	4.991808	-0.84477	0.402612	0.985398	-4.61027
NFKB1	NM_003998.2	0.003	-0.23798	5.894697	-0.84377	0.403166	0.985398	-4.61034
BCL2L1	NM_001191.2	0	-0.50513	8.045029	-0.84269	0.403765	0.985398	-4.61043
CD96	NM_005816.4	0.007	0.383725	7.013041	0.838707	0.405974	0.985398	-4.61073

ITGAE	NM_002208.4	0	-0.18784	6.65954	-0.83572	0.407633	0.985398	-4.61096
IFNGR1	NM_000416.1	0	-0.20409	8.702158	-0.83464	0.408237	0.985398	-4.61104
SIGLEC1	NM_023068.3	0.182	0.778307	3.727891	0.823779	0.414318	0.989111	-4.61186
PIN1	NM_006221.2	0	0.155316	6.481329	0.818174	0.417479	0.989111	-4.61228
TNFSF8	NM_001244.3	0.007	0.226357	5.552587	0.815575	0.418949	0.989111	-4.61247
HMGB1	NM_002128.4	0	-0.12624	9.282678	-0.81221	0.420857	0.989111	-4.61272
IL4R	NM_000418.2	0	0.285337	7.584634	0.810736	0.421695	0.989111	-4.61283
ABL1	NM_005157.3	0.014	0.253241	4.655954	0.810399	0.421886	0.989111	-4.61286
ALCAM	NM_001627.3	0.048	-0.35082	4.039628	-0.79932	0.428218	0.98974	-4.61367
ITGAL	NM_002209.2	0	0.185619	8.422515	0.798905	0.428454	0.98974	-4.6137
TBK1	NM_013254.2	0	-0.16467	6.159074	-0.79783	0.429069	0.98974	-4.61378
BATF	NM_006399.3	0.021	-0.3048	4.086645	-0.78905	0.434133	0.98974	-4.61441
TNFSF13B	NM_006573.4	0.003	0.368791	6.395955	0.788332	0.434549	0.98974	-4.61447
ANXA1	NM_000700.1	0	0.208035	11.06642	0.785638	0.43611	0.98974	-4.61466
LTB	NM_002341.1	0	-0.30025	9.575613	-0.78061	0.439035	0.98974	-4.61502
ATF2	NM_001256090.1	0	-0.13457	6.400367	-0.77961	0.439617	0.98974	-4.61509
TLR7	NM_016562.3	0.473	0.472547	1.309267	0.776929	0.44118	0.98974	-4.61528
BID	NM_001196.2	0.038	-0.23129	5.321876	-0.77194	0.444101	0.98974	-4.61563
CCL3	NM_002983.2	0	-0.2339	9.132729	-0.76575	0.44774	0.98974	-4.61607
IL1R2	NM_173343.1	0.027	-0.57804	6.622486	-0.76409	0.44872	0.98974	-4.61619
CLEC4C	NM_203503.1	0.455	-0.47749	1.795366	-0.76215	0.449862	0.98974	-4.61632
CD97	NM_078481.2	0	-0.14793	10.56114	-0.76119	0.450433	0.98974	-4.61639
LYN	NM_002350.1	0	-0.20414	8.436436	-0.74337	0.461039	0.994165	-4.61761
ICOSLG	NM_015259.4	0.055	0.416309	4.625151	0.738401	0.464024	0.994165	-4.61795
CCRL2	NM_003965.4	0.072	0.335191	4.064196	0.738224	0.464131	0.994165	-4.61796
GZMA	NM_006144.2	0.01	0.407997	8.241182	0.727091	0.470857	0.994165	-4.61871
CX3CR1	NM_001337.3	0.021	0.479913	5.709481	0.72272	0.473513	0.994165	-4.619
DUSP6	NM_001946.2	0	0.271119	8.452191	0.722469	0.473666	0.994165	-4.61902
IL18	NM_001562.2	0.024	0.299238	5.156855	0.711226	0.480537	0.994165	-4.61976
THBS1	NM_003246.2	0	0.400925	10.14454	0.710982	0.480687	0.994165	-4.61977

LILRA1	NM_006863.1	0.038	0.322241	5.268155	0.694129	0.491095	0.994165	-4.62086
CD3G	NM_000073.2	0.021	0.369027	6.097442	0.692977	0.49181	0.994165	-4.62093
PSMD7	NM_002811.3	0	-0.11643	8.416579	-0.68911	0.494219	0.994165	-4.62118
CASP8	NM_001228.4	0	-0.20461	9.094482	-0.68032	0.499712	0.994165	-4.62173
MAP2K1	NM_002755.2	0	0.165455	7.380962	0.677681	0.501371	0.994165	-4.6219
SMAD3	NM_005902.3	0	0.199731	6.1692	0.677581	0.501434	0.994165	-4.6219
CREB1	NM_004379.3	0	0.177966	5.941505	0.673589	0.503946	0.994165	-4.62215
TNFAIP3	NM_006290.2	0	-0.23231	11.64834	-0.67045	0.505927	0.994165	-4.62235
CCR4	NM_005508.4	0.062	0.363232	4.255131	0.6681	0.50741	0.994165	-4.62249
TLR10	NM_030956.2	0.096	-0.38968	3.445633	-0.66611	0.508668	0.994165	-4.62261
CCL3L1	NM_021006.4	0	-0.21301	9.002363	-0.66345	0.510354	0.994165	-4.62278
LILRB1	NM_001081637.1	0.007	0.319713	6.376977	0.663381	0.5104	0.994165	-4.62278
TIRAP	NM_148910.2	0.144	0.399525	2.708772	0.661775	0.511419	0.994165	-4.62288
IL6ST	NM_002184.2	0	-0.1595	8.370773	-0.65803	0.513799	0.994165	-4.6231
CD70	NM_001252.2	0.346	-0.38457	1.72365	-0.65631	0.514895	0.994165	-4.62321
CXCR5	NM_001716.3	0.171	0.422531	3.160619	0.654911	0.515789	0.994165	-4.62329
LILRB3	NM_006864.2	0	-0.29447	8.053259	-0.65486	0.51582	0.994165	-4.6233
EGR2	NM_000399.3	0.164	-0.56487	3.749044	-0.64869	0.519764	0.994165	-4.62367
LRP1	NM_002332.2	0	0.17198	7.384461	0.639945	0.525385	0.994165	-4.62419
FOS	NM_005252.2	0	-0.19543	13.08453	-0.63803	0.526619	0.994165	-4.6243
REL	NM_002908.2	0	0.169117	7.611904	0.637376	0.527042	0.994165	-4.62434
TLR2	NM_003264.3	0	-0.20523	7.830566	-0.63167	0.530734	0.994165	-4.62467
CD53	NM_001040033.1	0	-0.12374	10.19694	-0.62902	0.532447	0.994165	-4.62482
FCGR2A	NM_021642.3	0	-0.23441	10.11777	-0.62527	0.534886	0.994165	-4.62504
NLRP3	NM_001079821.2	0	0.205165	7.962799	0.624577	0.535337	0.994165	-4.62508
LGALS3	NM_001177388.1	0	0.205533	9.422522	0.622842	0.536466	0.994165	-4.62518
PYCARD	NM_013258.3	0	0.163601	7.012295	0.618319	0.539417	0.994165	-4.62544
SELL	NR_029467.1	0	-0.19857	9.916742	-0.61678	0.54042	0.994165	-4.62553
BST2	NM_004335.2	0	0.184229	6.957766	0.614742	0.541756	0.994165	-4.62564
PSMB7	NM_002799.2	0	-0.11192	8.640348	-0.61213	0.543467	0.994165	-4.62579

HLA-B	NM_005514.6	0	-0.11154	13.51642	-0.61044	0.544576	0.994165	-4.62589
CSF2RB	NM_000395.2	0	-0.21024	8.902947	-0.60504	0.548127	0.994165	-4.62619
STAT5B	NM_012448.3	0	0.206911	7.056284	0.604949	0.548188	0.994165	-4.62619
FUT7	NM_004479.3	0.058	-0.34724	5.104936	-0.59515	0.55466	0.994165	-4.62674
FYN	NM_002037.3	0	0.184052	8.416811	0.594935	0.554804	0.994165	-4.62675
CD19	NM_001770.4	0.055	0.27334	4.931502	0.589208	0.558606	0.994165	-4.62706
TNFSF12	NM_003809.2	0	0.181839	7.372529	0.586243	0.56058	0.994165	-4.62722
MFGE8	NM_001114614.1	0.055	0.323355	3.889892	0.583436	0.562452	0.994165	-4.62737
DOCK9	NM_001130048.1	0.024	0.181193	4.890024	0.580557	0.564374	0.994165	-4.62753
CD37	NM_001774.2	0.003	-0.21734	7.261653	-0.5775	0.566418	0.994165	-4.62769
CD27	NM_001242.4	0.031	0.310402	5.179405	0.577402	0.566486	0.994165	-4.6277
CD48	NM_001778.2	0	-0.12673	10.51527	-0.5754	0.567829	0.994165	-4.6278
CCL20	NM_004591.1	0.411	-0.39925	2.384007	-0.57153	0.570423	0.994165	-4.62801
CFD	NM_001928.2	0	0.211902	7.118607	0.568597	0.572397	0.994165	-4.62816
CXCL2	NM_002089.3	0.058	-0.43033	6.548646	-0.56681	0.573602	0.994165	-4.62826
CREB5	NM_182898.2	0.007	0.224042	6.674693	0.562702	0.576372	0.994165	-4.62847
CD44	NM_001001392.1	0	0.143419	11.23368	0.551497	0.583964	0.994165	-4.62905
TNF	NM_000594.2	0	-0.20596	7.922257	-0.5439	0.589136	0.994165	-4.62943
MICA	NM_000247.1	0.178	-0.32173	2.855832	-0.54322	0.589605	0.994165	-4.62947
PLA2G6	NM_001004426.1	0.034	0.183056	4.186342	0.539663	0.592034	0.994165	-4.62965
IL2RG	NM_000206.1	0	-0.14247	8.710508	-0.53404	0.595888	0.994165	-4.62993
KLRB1	NM_002258.2	0.007	0.264786	7.841228	0.531651	0.597528	0.994165	-4.63004
ITGA6	NM_000210.1	0.062	-0.22876	4.602096	-0.53023	0.598507	0.994165	-4.63011
CD8B	NM_004931.3	0.024	-0.26212	5.719368	-0.52864	0.599601	0.994165	-4.63019
SERPING1	NM_000062.2	0.024	0.365074	5.143837	0.528221	0.599887	0.994165	-4.63021
CD164	NM_006016.4	0	0.094587	8.324403	0.520053	0.605522	0.994165	-4.63061
NFATC2	NM_012340.3	0	0.115725	7.295299	0.515135	0.608927	0.994165	-4.63085
ITGA2B	NM_000419.3	0	-0.22403	7.979841	-0.50818	0.613758	0.994165	-4.63118
STAT3	NM_139276.2	0	-0.10941	8.892825	-0.5026	0.617648	0.994165	-4.63144
CD83	NM_004233.3	0	0.154088	10.37966	0.50229	0.617861	0.994165	-4.63145

CTLA4	NM_005214.3	0.01	0.220688	5.919118	0.493286	0.624159	0.994165	-4.63187
IL18R1	NM_003855.2	0.106	0.323629	4.143967	0.491665	0.625295	0.994165	-4.63194
CYFIP2	NM_001037332.2	0	0.117998	7.808367	0.490391	0.62619	0.994165	-4.632
CD46	NM_172350.1	0	-0.07911	8.867534	-0.48604	0.629244	0.994165	-4.6322
IFNAR1	NM_000629.2	0.171	-0.28254	2.773402	-0.48542	0.629687	0.994165	-4.63222
CD3D	NM_000732.4	0.003	0.248495	8.20291	0.4844	0.630402	0.994165	-4.63227
TIGIT	NM_173799.2	0.096	-0.27195	3.37671	-0.48022	0.633349	0.994165	-4.63246
PECAM1	NM_000442.3	0	-0.08984	9.131581	-0.47871	0.634415	0.994165	-4.63252
GTF3C1	NM_001520.3	0.007	0.095288	5.587329	0.477991	0.634922	0.994165	-4.63256
SLC11A1	NM_000578.2	0	0.145084	8.524329	0.477509	0.635262	0.994165	-4.63258
CEBPB	NM_005194.2	0	-0.12506	9.624956	-0.47455	0.637356	0.994165	-4.63271
TLR4	NM_138554.2	0.003	-0.16331	6.848706	-0.47419	0.637611	0.994165	-4.63272
GZMH	NM_033423.3	0.007	0.249934	8.19661	0.473861	0.637842	0.994165	-4.63274
SLAMF7	NM_021181.3	0	-0.18109	6.25628	-0.4737	0.637953	0.994165	-4.63275
MAP3K5	NM_005923.3	0	-0.10371	7.690549	-0.47281	0.638584	0.994165	-4.63278
MAPK3	NM_001040056.1	0	0.109976	6.620495	0.470788	0.640018	0.994165	-4.63287
MAPK8	NM_002750.2	0.017	-0.1765	4.369522	-0.46766	0.642236	0.994165	-4.63301
IRF1	NM_002198.1	0	0.16504	10.24649	0.462067	0.646213	0.994165	-4.63325
BTLA	NM_181780.2	0.034	0.22183	4.925785	0.46024	0.647514	0.994165	-4.63333
ATG5	NM_004849.2	0	-0.11155	6.140491	-0.45925	0.648218	0.994165	-4.63337
KLRK1	NM_007360.3	0.007	0.218351	8.024475	0.457412	0.64953	0.994165	-4.63345
JAK2	NM_004972.2	0	0.096801	8.03719	0.45527	0.651058	0.994165	-4.63354
ICAM1	NM_000201.2	0	-0.18043	8.015949	-0.45222	0.653237	0.994165	-4.63367
HAVCR2	NM_032782.3	0.014	0.142535	5.163379	0.449725	0.655022	0.994165	-4.63377
CD3E	NM_000733.2	0.003	0.219274	7.823806	0.449455	0.655215	0.994165	-4.63378
ICAM2	NM_000873.3	0	-0.13245	6.481238	-0.4487	0.655757	0.994165	-4.63382
BCL6	NM_001706.2	0	-0.15977	9.634073	-0.44485	0.658518	0.994165	-4.63398
IFI16	NM_005531.1	0	-0.1188	6.92399	-0.44449	0.658772	0.994165	-4.63399
LAG3	NM_002286.5	0.065	-0.28283	4.236761	-0.43998	0.662011	0.994165	-4.63418
ATG7	NM_001136031.2	0.014	-0.16214	5.794977	-0.43628	0.664675	0.994165	-4.63433

GPI	NM_000175.2	0	-0.07737	7.703165	-0.4354	0.665307	0.994165	-4.63436
TAB1	NM_153497.2	0.027	0.218989	4.629758	0.43257	0.66735	0.994165	-4.63448
NOTCH1	NM_017617.3	0.007	-0.1265	6.291812	-0.43057	0.668795	0.994165	-4.63456
IFIT1	NM_001548.3	0.079	-0.2889	4.978397	-0.42993	0.669259	0.994165	-4.63458
OAS3	NM_006187.2	0.024	0.268216	5.89985	0.42982	0.669335	0.994165	-4.63459
NOD2	NM_022162.1	0	0.111861	6.654154	0.428658	0.670174	0.994165	-4.63463
IL15	NM_172174.1	0.024	0.143066	4.544888	0.427965	0.670675	0.994165	-4.63466
RORA	NM_134261.2	0.003	0.162211	6.734638	0.423973	0.673563	0.994165	-4.63482
ICOS	NM_012092.2	0.048	0.201361	5.329691	0.423305	0.674046	0.994165	-4.63485
CD6	NM_006725.3	0.014	-0.19574	6.224248	-0.42095	0.675756	0.994165	-4.63494
CXCR1	NM_000634.2	0.38	0.308861	2.902891	0.414014	0.680789	0.994165	-4.63521
CD247	NM_198053.1	0.007	0.189288	7.865711	0.411358	0.682722	0.994165	-4.63531
ISG20	NM_002201.4	0	0.112176	7.953218	0.407321	0.685663	0.994165	-4.63546
CCR3	NM_001837.2	0.455	-0.28673	2.209823	-0.40516	0.687243	0.994165	-4.63554
TNFSF4	NM_003326.2	0.007	0.189517	5.133709	0.398057	0.692431	0.994165	-4.63581
IL2RA	NM_000417.1	0.318	0.216989	2.036021	0.39782	0.692604	0.994165	-4.63582
POU2F2	NM_002698.2	0.003	0.096312	6.763863	0.397023	0.693188	0.994165	-4.63585
ST6GAL1	NM_003032.2	0.003	0.107017	7.072304	0.395042	0.694639	0.994165	-4.63592
CD2	NM_001767.3	0.072	0.245497	4.124985	0.392664	0.696382	0.994165	-4.63601
CXCL1	NM_001511.1	0.144	-0.27934	5.006703	-0.38697	0.700562	0.994165	-4.63622
HLA-DQA1	NM_002122.3	0.325	-0.39967	5.461318	-0.38114	0.704853	0.994165	-4.63642
YTHDF2	NM_001172828.1	0.01	-0.07365	5.948496	-0.36949	0.713463	0.994165	-4.63683
CD55	NM_000574.3	0.264	-0.25549	3.129697	-0.36925	0.71364	0.994165	-4.63684
PTPRC	NM_080921.3	0	0.11398	9.185927	0.368906	0.713891	0.994165	-4.63685
TRAF2	NM_021138.3	0.048	0.162844	3.805718	0.364361	0.717259	0.994165	-4.63701
HCK	NM_002110.2	0	0.117748	9.204422	0.362424	0.718696	0.994165	-4.63707
TAP2	NM_000544.3	0	0.073012	7.801693	0.357407	0.722423	0.994165	-4.63724
AMICA1	NM_153206.2	0	0.119384	9.092276	0.354876	0.724306	0.994165	-4.63732
JAM3	NM_032801.3	0.027	0.215841	5.043229	0.351663	0.726698	0.994165	-4.63743
LY9	NM_001033667.1	0	-0.09602	6.85593	-0.34827	0.729227	0.994165	-4.63754

CD84	NM_001184879.1	0	0.07828	6.851062	0.347664	0.72968	0.994165	-4.63756
FCGR2B	NM_001002273.1	0.048	0.162018	5.255136	0.344949	0.731706	0.994165	-4.63765
BST1	NM_004334.2	0	0.098235	8.513588	0.339743	0.735598	0.994165	-4.63781
IFI27	NM_005532.3	0.202	0.440872	4.230699	0.339638	0.735676	0.994165	-4.63782
IL21R	NM_021798.2	0.082	-0.19217	3.672715	-0.3371	0.737573	0.994165	-4.6379
LCK	NM_005356.2	0.003	0.155404	7.504193	0.331861	0.741502	0.994165	-4.63806
ILF3	NM_001137673.1	0	0.059715	8.590258	0.32966	0.743154	0.994165	-4.63813
TARP	NM_001003799.1	0.003	0.130511	6.786451	0.329066	0.743601	0.994165	-4.63815
MAPK1	NM_138957.2	0	0.074185	8.055641	0.325942	0.745948	0.994165	-4.63824
IFITM1	NM_003641.3	0	-0.11274	10.37725	-0.32429	0.747193	0.994165	-4.63829
TFRC	NM_003234.1	0	0.102544	7.865515	0.323011	0.748152	0.994165	-4.63833
ADA	NM_000022.2	0.007	-0.07747	5.661062	-0.31603	0.753411	0.994165	-4.63854
CD58	NM_001779.2	0	-0.06736	7.654274	-0.3144	0.754639	0.994165	-4.63859
CCND3	NM_001760.2	0	0.085204	8.792868	0.310473	0.757605	0.994165	-4.6387
LCP1	NM_002298.4	0	0.070607	11.29639	0.309731	0.758165	0.994165	-4.63872
EP300	NM_001429.2	0	-0.06709	8.483865	-0.30944	0.758388	0.994165	-4.63873
ICAM3	NM_002162.3	0	-0.05269	9.41513	-0.30697	0.760253	0.994165	-4.6388
OSM	NM_020530.4	0.014	-0.16948	7.354942	-0.30281	0.7634	0.994165	-4.63892
IL6	NM_000600.1	0.027	0.139568	5.404137	0.301084	0.764708	0.994165	-4.63897
MAP2K2	NM_030662.2	0	0.057615	8.313204	0.296004	0.76856	0.994165	-4.63911
IL7R	NM_002185.2	0.01	-0.15085	7.403287	-0.29546	0.768975	0.994165	-4.63913
DDX58	NM_014314.3	0.031	-0.14128	5.685544	-0.29337	0.770561	0.994165	-4.63918
CD28	NM_001243078.1	0.178	-0.18342	3.178077	-0.29093	0.772412	0.994165	-4.63925
SERPINB2	NM_002575.1	0.075	0.211213	5.698605	0.285585	0.776479	0.994165	-4.63939
PSMB8	NM_004159.4	0	0.057971	8.725179	0.280499	0.780353	0.994165	-4.63953
PDGFC	NM_016205.2	0.147	-0.15157	3.22263	-0.28022	0.780566	0.994165	-4.63954
RUNX3	NM_004350.1	0	-0.07754	8.801292	-0.27499	0.784554	0.994165	-4.63967
MARCO	NM_006770.3	0.031	0.163928	5.389415	0.269786	0.788532	0.994165	-4.6398
TAL1	NM_003189.2	0.058	-0.16669	4.597146	-0.26973	0.788573	0.994165	-4.63981
TNFRSF1B	NM_001066.2	0	-0.07258	9.713878	-0.26895	0.789169	0.994165	-4.63983

MYD88	NM_002468.3	0	0.064749	8.569967	0.266708	0.790887	0.994165	-4.63988
C1QBP	NM_001212.3	0	-0.06928	7.333467	-0.26583	0.791559	0.994165	-4.6399
MME	NM_000902.2	0.216	-0.24678	5.016458	-0.26266	0.793989	0.994165	-4.63998
SOCS1	NM_003745.1	0.024	0.112958	5.50065	0.258392	0.797258	0.994165	-4.64009
CR1	NM_000651.4	0	0.102214	8.329865	0.257305	0.798091	0.994165	-4.64011
SMAD2	NM_005901.5	0	0.055921	6.606409	0.256134	0.79899	0.994165	-4.64014
CD81	NM_004356.3	0	0.061223	8.858433	0.254246	0.800439	0.994165	-4.64018
TYK2	NM_003331.3	0	0.048579	6.846544	0.241974	0.809876	0.994165	-4.64047
IFIH1	NM_022168.2	0.003	0.078925	6.32183	0.241564	0.810192	0.994165	-4.64048
GZMM	NM_005317.2	0.01	0.087518	5.838119	0.240757	0.810814	0.994165	-4.6405
PDGFRB	NM_002609.3	0.349	0.12248	2.205225	0.239756	0.811585	0.994165	-4.64052
CASP3	NM_032991.2	0.007	0.069312	5.089648	0.236966	0.813736	0.994165	-4.64058
DPP4	NM_001935.3	0.366	-0.14936	1.607852	-0.23468	0.815502	0.994165	-4.64063
IRF4	NM_002460.1	0.003	-0.10634	6.239596	-0.22574	0.822405	0.994165	-4.64082
CKLF	NM_181640.2	0	0.081193	7.453505	0.223703	0.82398	0.994165	-4.64087
ISG15	NM_005101.3	0	-0.10876	7.06732	-0.22216	0.825172	0.994165	-4.6409
ITGA4	NM_000885.4	0	-0.04032	7.618351	-0.22153	0.825663	0.994165	-4.64091
C3AR1	NM_004054.2	0.147	0.154091	4.120067	0.219546	0.827197	0.994165	-4.64095
EWSR1	NM_013986.3	0	0.031071	8.259702	0.217981	0.828409	0.994165	-4.64098
TXK	NM_003328.1	0.202	0.113441	3.152687	0.217104	0.829088	0.994165	-4.641
MEF2C	NM_002397.3	0.01	0.104641	6.144537	0.214204	0.831335	0.994165	-4.64106
ITGB1	NM_033666.2	0	0.045089	9.265407	0.212294	0.832816	0.994165	-4.6411
IL6R	NM_000565.2	0	-0.0566	6.601497	-0.20632	0.837449	0.994165	-4.64122
IL2RB	NM_000878.2	0.014	0.104237	6.905447	0.204181	0.839113	0.994165	-4.64126
IL23A	NM_016584.2	0.291	0.122669	2.108989	0.197045	0.844661	0.994165	-4.64139
LILRA5	NM_181879.2	0	0.069625	9.380127	0.196983	0.844709	0.994165	-4.64139
TFE3	NM_006521.3	0.007	0.070751	4.812652	0.1948	0.846408	0.994165	-4.64143
ICAM4	NM_001039132.1	0.068	0.110414	4.095334	0.194104	0.84695	0.994165	-4.64145
SLAMF1	NM_003037.2	0.205	0.124256	2.548404	0.19392	0.847093	0.994165	-4.64145
CMKLR1	NM_004072.1	0.301	0.149533	2.694735	0.192876	0.847905	0.994165	-4.64147

CD8A	NM_001768.5	0	0.080208	7.690719	0.189225	0.85075	0.994165	-4.64153
CD86	NM_175862.3	0.024	0.095287	5.490634	0.185816	0.853407	0.994165	-4.64159
FEZ1	NM_005103.4	0.414	-0.10399	1.798337	-0.18142	0.856837	0.994165	-4.64167
FCER1A	NM_002001.2	0.12	0.136665	4.747797	0.179177	0.858586	0.994165	-4.64171
F13A1	NM_000129.3	0	0.079027	9.711828	0.175158	0.861725	0.994165	-4.64177
PPBP	NM_002704.2	0	-0.08526	11.92664	-0.17379	0.86279	0.994165	-4.64179
IRF2	NM_002199.3	0	-0.05405	6.138062	-0.17249	0.863807	0.994165	-4.64182
ITGA5	NM_002205.2	0	0.038109	7.247719	0.171759	0.864381	0.994165	-4.64183
CLEC4A	NM_194448.2	0	-0.04086	8.014145	-0.1714	0.86466	0.994165	-4.64183
THBD	NM_000361.2	0.003	0.079565	8.602248	0.168465	0.866956	0.994165	-4.64188
HLA-G	NM_002127.4	0	0.043825	7.116824	0.165659	0.869152	0.994165	-4.64192
ARG1	NM_000045.2	0.408	-0.11843	2.621635	-0.16285	0.87135	0.994165	-4.64197
CD200	NM_005944.5	0.226	-0.08549	2.747506	-0.16075	0.872991	0.994165	-4.642
TNFRSF8	NM_152942.2	0.373	-0.09138	1.852716	-0.15867	0.874623	0.994165	-4.64203
XCL2	NM_003175.3	0.038	0.088852	5.603926	0.158303	0.874912	0.994165	-4.64204
APP	NM_000484.3	0	0.033688	7.831006	0.157674	0.875404	0.994165	-4.64204
CD5	NM_014207.2	0.007	0.068243	7.011149	0.156417	0.87639	0.994165	-4.64206
S100A8	NM_002964.3	0	0.061241	13.06959	0.155946	0.876758	0.994165	-4.64207
ANP32B	NM_006401.2	0	-0.04245	8.323172	-0.15467	0.877761	0.994165	-4.64209
FCGR3A	NM_000569.6	0	0.062081	8.95422	0.151797	0.880011	0.994165	-4.64213
RELB	NM_006509.2	0	-0.04777	6.880603	-0.15047	0.881056	0.994165	-4.64215
NFATC3	NM_004555.2	0	-0.03314	6.823623	-0.14965	0.881693	0.994165	-4.64216
MEFV	NM_000243.2	0.003	0.062977	7.438844	0.149147	0.88209	0.994165	-4.64217
MAP4K2	NM_004579.2	0	-0.02642	6.850069	-0.14214	0.887587	0.994165	-4.64226
SBNO2	NM_014963.2	0.003	0.047515	6.650402	0.1405	0.888879	0.994165	-4.64228
STAT2	NM_005419.2	0	0.050234	7.744843	0.136264	0.892207	0.994165	-4.64234
TICAM1	NM_014261.1	0.003	-0.03577	6.484478	-0.13526	0.892998	0.994165	-4.64235
FPR2	NM_001462.3	0.041	-0.07024	5.454238	-0.13194	0.895607	0.994165	-4.64239
FLT3LG	NM_001459.3	0	-0.03383	7.123105	-0.12936	0.897636	0.994165	-4.64243
MAVS	NM_020746.3	0.031	0.048897	4.531542	0.129247	0.897726	0.994165	-4.64243

IFNG	NM_000619.2	0.007	-0.05665	6.698489	-0.12757	0.899049	0.994165	-4.64245
IRAK1	NM_001569.3	0	-0.02466	7.154834	-0.12701	0.899483	0.994165	-4.64245
CYLD	NM_015247.1	0	-0.02606	8.487604	-0.12384	0.901985	0.994165	-4.64249
BAX	NM_138761.3	0	0.019117	8.065971	0.120445	0.904656	0.994165	-4.64253
JAK3	NM_000215.2	0	0.03046	7.815328	0.118074	0.906523	0.994165	-4.64256
PIK3CD	NM_005026.3	0	0.044758	7.383324	0.117569	0.906922	0.994165	-4.64256
KIR3DL2	NM_006737.2	0.253	0.059186	2.403673	0.111139	0.91199	0.994281	-4.64263
ITGAX	NM_000887.3	0	0.031248	9.221799	0.110137	0.91278	0.994281	-4.64264
TNFRSF1A	NM_001065.2	0	0.026647	7.511156	0.106252	0.915845	0.994281	-4.64268
SLAMF6	NM_001184714.1	0.267	0.069651	2.349106	0.105125	0.916734	0.994281	-4.64269
VEGFA	NM_001025366.1	0	-0.03126	8.070343	-0.10457	0.917173	0.994281	-4.6427
TCF7	NM_003202.2	0.021	0.048109	6.956808	0.098442	0.92201	0.996051	-4.64275
CD99	NM_002414.3	0	0.027381	9.897383	0.096948	0.923189	0.996051	-4.64277
HLA-DQB1	NM_002123.3	0.315	-0.08819	5.047712	-0.09163	0.927389	0.996051	-4.64282
TANK	NM_004180.2	0	-0.01873	8.706135	-0.08385	0.933541	0.996051	-4.64288
ENG	NM_001114753.1	0.051	0.042011	4.250448	0.078181	0.938024	0.996051	-4.64292
TLR8	NM_016610.2	0.014	0.026934	5.419839	0.077204	0.938796	0.996051	-4.64293
LAMP1	NM_005561.3	0	-0.0116	8.732198	-0.07134	0.943434	0.996051	-4.64297
CXCL5	NM_002994.3	0.027	0.038895	5.848684	0.069412	0.944963	0.996051	-4.64298
IFI35	NM_005533.3	0.027	-0.04063	5.504062	-0.06924	0.945102	0.996051	-4.64298
CXCL3	NM_002090.2	0.267	0.067806	3.780252	0.066652	0.947148	0.996051	-4.643
IL17RA	NM_014339.6	0.003	-0.01907	7.20004	-0.06309	0.94997	0.996051	-4.64302
NT5E	NM_002526.2	0.137	-0.03996	2.662419	-0.06302	0.950024	0.996051	-4.64302
IKBKB	NM_001556.1	0.024	-0.02095	4.93791	-0.06256	0.950385	0.996051	-4.64303
ITGB3	NM_000212.2	0.003	-0.03475	7.078078	-0.06181	0.950982	0.996051	-4.64303
CXCL16	NM_001100812.1	0.003	-0.02418	7.176502	-0.05954	0.952777	0.996051	-4.64304
IRF7	NM_001572.3	0	-0.01686	7.693078	-0.05335	0.957687	0.996051	-4.64308
NFATC1	NM_172389.1	0.007	-0.01467	5.167577	-0.04863	0.961429	0.996051	-4.6431
JAK1	NM_002227.1	0	0.007636	9.217217	0.047975	0.961944	0.996051	-4.6431
TNFSF10	NM_003810.2	0	-0.01957	7.566405	-0.0479	0.962002	0.996051	-4.6431

STAT6	NM_003153.3	0	-0.0128	9.391131	-0.04758	0.96226	0.996051	-4.6431
IL13RA1	NM_001560.2	0	-0.01696	7.264141	-0.04525	0.964104	0.996051	-4.64311
IGF2R	NM_000876.1	0.003	-0.01115	7.211368	-0.0426	0.966205	0.996051	-4.64312
IRAK4	NM_016123.1	0.007	-0.01304	5.505175	-0.04243	0.966337	0.996051	-4.64312
SELPLG	NM_001206609.1	0	-0.01511	7.208536	-0.04085	0.967593	0.996051	-4.64313
SH2D1A	NM_001114937.2	0.024	0.014992	5.607209	0.029994	0.976202	0.998197	-4.64317
TP53	NM_000546.2	0.014	0.01142	5.297269	0.029161	0.976862	0.998197	-4.64317
CCL5	NM_002985.2	0	-0.0112	10.64813	-0.02847	0.977409	0.998197	-4.64317
IL18RAP	NM_003853.2	0.065	0.018658	5.650186	0.027946	0.977826	0.998197	-4.64317
CTSG	NM_001911.2	0.462	0.017642	1.970743	0.023724	0.981176	0.999535	-4.64318
TLR6	NM_006068.2	0.024	0.006344	5.028522	0.018612	0.985232	0.999899	-4.64319
PLAUR	NM_001005376.1	0	-0.006	9.602724	-0.01795	0.985757	0.999899	-4.64319
CHUK	NM_001278.3	0	-0.0036	6.830635	-0.01278	0.989862	0.999899	-4.6432
ETS1	NM_005238.3	0	-0.0031	9.049235	-0.0098	0.992226	0.999899	-4.6432
IKBKG	NM_003639.2	0.003	0.001145	6.422169	0.005787	0.995408	0.999899	-4.64321
ITK	NM_005546.3	0.007	-0.00237	7.125809	-0.00553	0.99561	0.999899	-4.64321
CAMP	NM_004345.3	0.253	-0.00146	3.309635	-0.00185	0.998534	0.999899	-4.64321
CSF1	NM_000757.4	0.236	-0.00059	3.534624	-0.00088	0.999304	0.999899	-4.64321
PIK3CG	NM_002649.2	0.003	-2.48E-05	7.102338	-0.00013	0.999899	0.999899	-4.64321

D-Rd, daratumumab, lenalidomide, and dexamethasone; C, Cycle; D, Day; Rd, lenalidomide and dexamethasone.

Table S4. Corresponding median values for Figures 2A, 2D, and 2E.
(A) Median ratio of $CD56^{dim}$ to $CD56^{bright}$ cells relative to the total number of NK cells at baseline and upon treatment. (B) Median percentage of $CD137^{+}CD56^{dim}$ and $CD56^{bright}$ NK cells relative to total $CD56^{dim}$ and $CD56^{bright}$ NK cells, respectively. (C) Median percentages of subtypes of CD27 and CD69 NK cells relative to total NK cells.

**A.**					
Treatment	log2ratio at C1D1	log2ratio at C3D1	Median ratio at C1D1	Median ratio at C3D1	Paired Wilcoxon test *P* value
D-Rd	4.41	2.31	21.2	4.97	5.72×10^{-6}
Rd	4.3	4.21	19.8	18.5	0.0684
**B.**					
Treatment	Channel	Population	Median % at C1D1	Median % at C3D1	Paired wilcoxon test *P* value
D-Rd	CD137	CD56dim NK	23.4	31.8	7.22×10^{-6}
D-Rd	CD137	CD56Br NK	19.8	22.8	
Rd	CD137	CD56dim NK	20.7	20.4	0.619
Rd	CD137	CD56Br NK	16.1	17.1	
**C.**					
Treatment	CD27	CD69	Median % at C1D1	Median % at C3D1	Paired wilcoxon test *p* value
DRd	CD27-neg	CD69-neg	63.7	29.8	2.93×10^{-5}
DRd	CD27-neg	CD69-pos	22.7	21.4	0.882
DRd	CD27-pos	CD69-neg	8.4	23.5	4×10^{-6}
DRd	CD27-pos	CD69-pos	4.05	18.2	4×10^{-6}
Rd	CD27-neg	CD69-neg	67.5	66	0.458
Rd	CD27-neg	CD69-pos	18.4	18.5	0.734
Rd	CD27-pos	CD69-neg	9.7	9.6	0.562
Rd	CD27-pos	CD69-pos	3.2	3.6	0.137

NK, natural killer; C, Cycle; D, Day; D-Rd, daratumumab, lenalidomide, and dexamethasone; Rd, lenalidomide and dexamethasone.

Table S5. Corresponding median values for Figures 3A and 3C.
Median proportion of total T cells, CD8 T cells, CD4 T cells, $T_{EM}RA$ CD38 T cells, and T_{EM} CD38 T cells of total cells.

Treatment	Median proportion of cells at C1D1	Median proportion of cells at C3D1	Paired Wilcoxon test *P* value
**Total T cells**			
D-Rd	0.550	0.630	9.04×10^{-4}
Rd	0.534	0.507	0.228
**CD8 T cells**			
D-Rd	0.202	0.322	2.86×10^{-7}
Rd	0.255	0.218	0.596
**CD4 T cells**			
D-Rd	0.273	0.236	0.011
Rd	0.227	0.244	0.376
**$T_{EM}RA$ CD8 T cells**			
D-Rd	0.0729	0.0905	0.163
Rd	0.0723	0.0354	1.61×10^{-5}
**T_{EM} CD8 T cells**			
D-Rd	0.012	0.0347	9.96×10^{-7}
Rd	0.0166	0.0258	0.00583

T_{EM}, effector memory T cells; $T_{EM}RA$, effector memory CD45RA^{+} T cells; C, Cycle; D, Day; D-Rd, daratumumab, lenalidomide, and dexamethasone; Rd, lenalidomide and dexamethasone.

Table S6. Corresponding median values for Figures 4A and 4B.
Median CD38 signal intensity of CD38^{+} T_{reg} cells and MDSCs.

Treatment	Median MSI of cells at C1D1	Median MSIof cells at C3D1	Paired Wilcoxon test *P* value
**CD38 T_{reg} cells**			
D-Rd	0.623	0.246	5.32×10^{-6}
Rd	0.54	0.597	0.944
**MDSCs**			
D-Rd	2.300603	1.654633	1.28×10^{-7}
Rd	2.196249	2.113935	0.465

MDSC, myeloid-derived suppressor cell; MSI, mean signal intensity; C, Cycle; D, Day; D-Rd, daratumumab, lenalidomide, and dexamethasone; Rd, lenalidomide and dexamethasone.

Table S7. Corresponding median values for Figure 5A.
Median T-cell repertoire clonality.

Treatment	Median at C1D1	Median at C3D1	Paired Wilcoxon test *P* value
**T-cell repertoire clonality**			
D-Rd	0.174	0.263	3.000265×10^{-12}
Rd	0.170	0.172	0.665267

C, Cycle; D, Day; D-Rd, daratumumab, lenalidomide, and dexamethasone; Rd, lenalidomide and dexamethasone.

Supplemental Figures

Figure S1. Quality control assessment of CyTOF® by (A) Earth mover's distance and (B) MEM analysis.

(A) Quality control of this sample set, by earth mover's distance analysis and inspection of the subsequent hierarchical clustering (UPGMA) tree structure, revealed the expected clustering of the control samples (shown in blue). (B) Per-channel MEM scores further confirmed the absence of batch effects. CyTOF, cytometry by time-of-flight; lympho, lymphocytes; MEM, marker enrichment modeling; mono, monocytes; SPADE, spanning-tree progression analysis of density-normalized events; UPGMA, unweighted pair group method with arithmetic mean.

A.

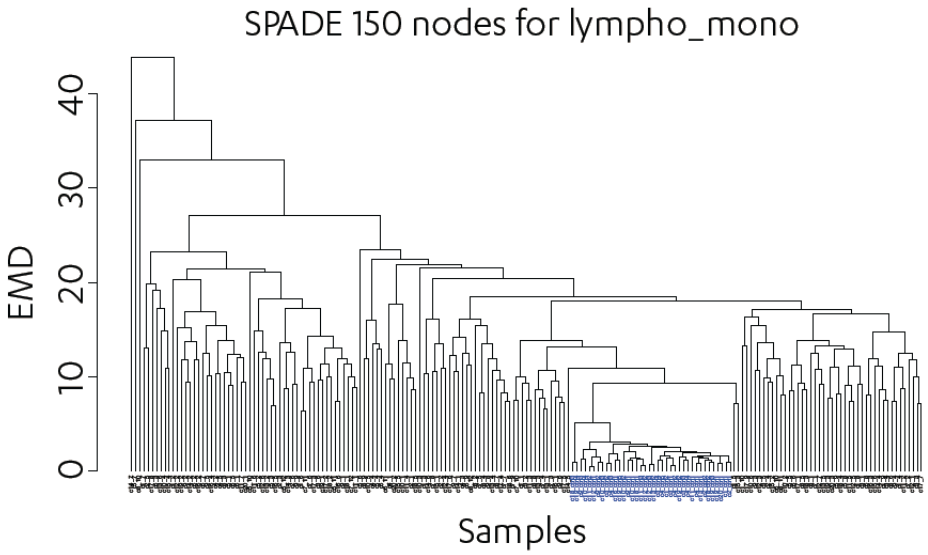

B.

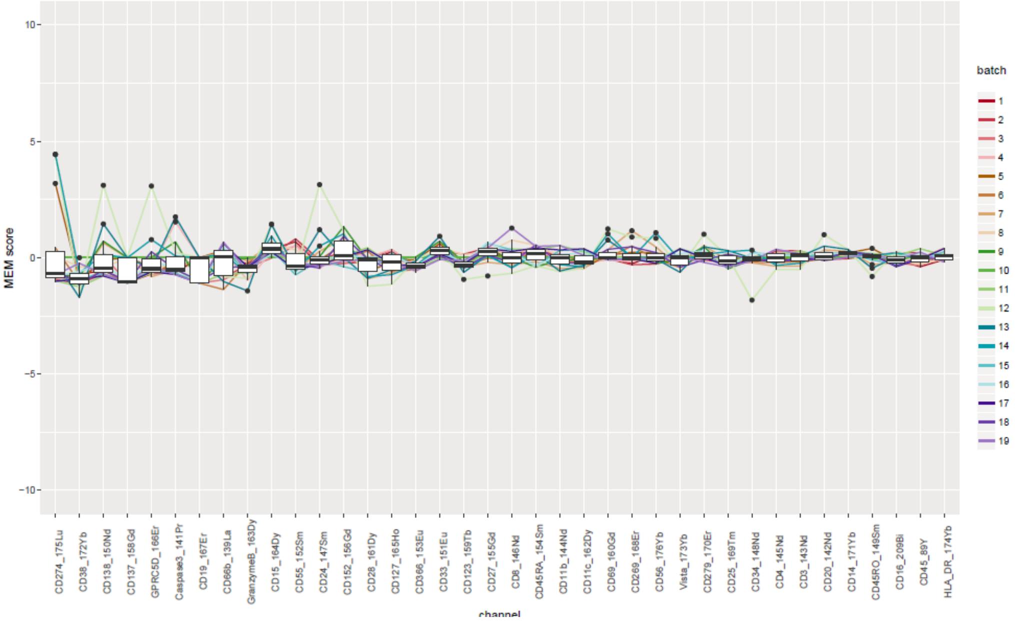

Figure S2. Quality control assessment of NanoString results.
(A) Sample log2(Count) median, interquartile range, and whiskers for each class of probe, grouped by analysis plate. Samples with median log2(Count) values ≤3 were removed from the analysis due to poor quantification. (B) Across that study, with 1 exception, positive control probes exhibited linear behavior and were confidently detected above the noise floor. IQR, interquartile range.

A.

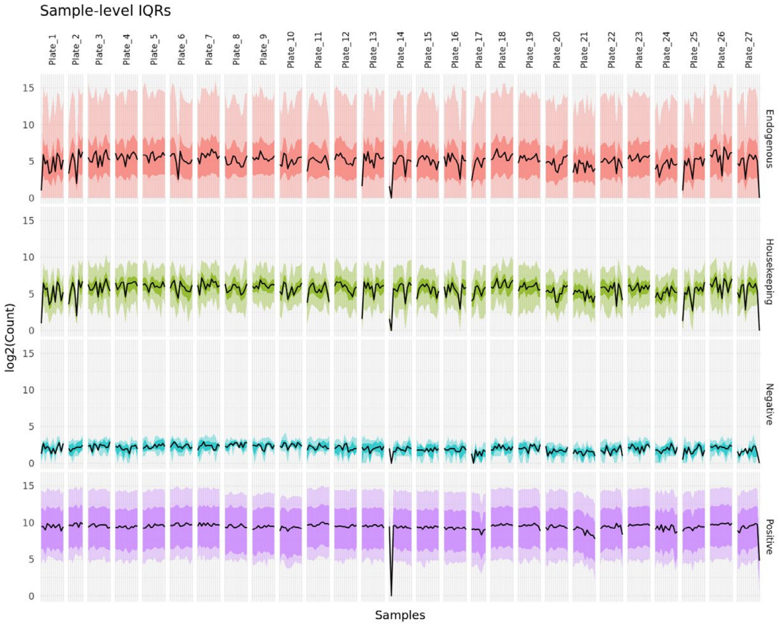

B.

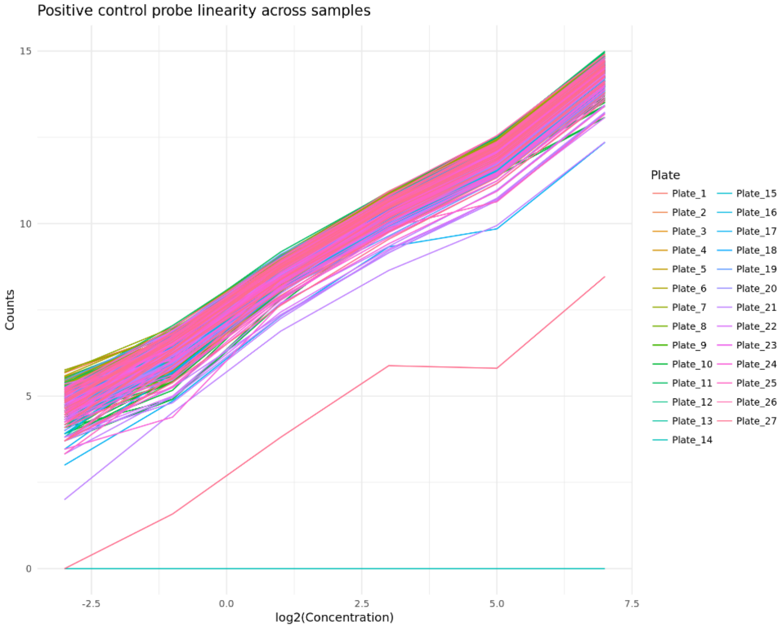

Figure S3. Differential expression testing results on the NanoString platform of baseline versus on-treatment samples of D-Rd– and Rd-treated patients, for the standard NanoString data with significantly regulated genes by both treatments highlighted (with adjusted P<0.05), and for the NanoString data corrected for the CyTOF-derived cellular abundances with the NK-cell and T-cell genes highlighted.

Baseline versus on-treatment samples of (A and B, left) D-Rd– and (A and B, right) Rd-treated patients, for (A) the standard NanoString data with significantly regulated genes by both treatments highlighted (with adjusted *P*<0.05; red dots), and (B) for the NanoString data corrected for the CyTOF-derived cellular abundances with the NK-cell and T-cell genes highlighted (blue dots). (C) Venn diagram with number of shared and unique differentially expressed genes, based on standard NanoString data analysis. C, Cycle; CyTOF, cytometry by time-of-flight; D, day.

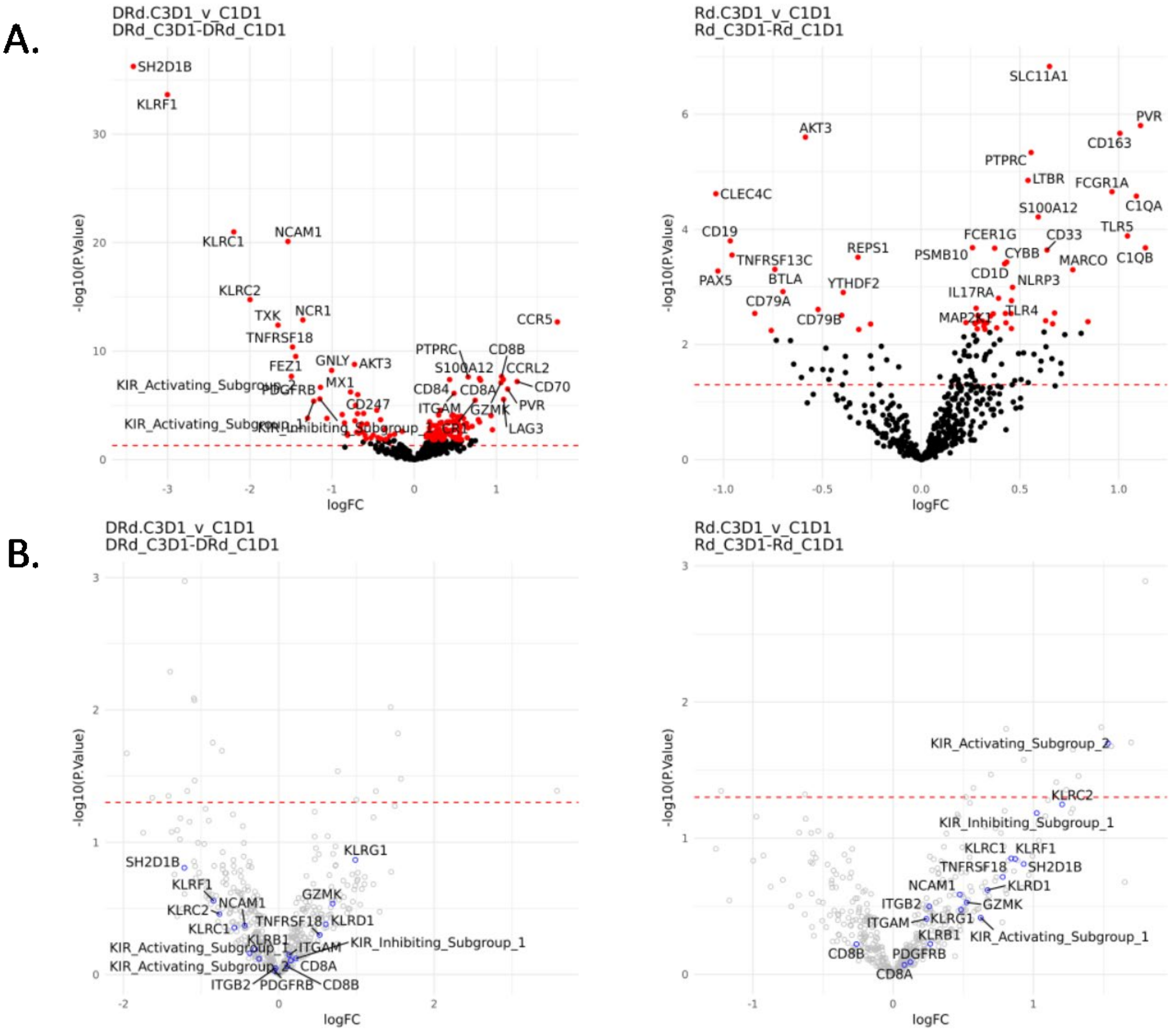

C.

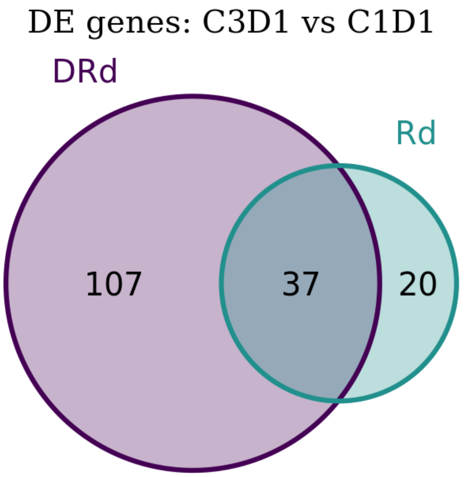

Figure S4. Phenotype of persistent NK cells were upon D-Rd treatment.
(A) Three patients treated with D-Rd had levels of NK cells that stayed stable or increased (NK increased) and were phenotypically distinct from the rest of the patient population (NK decreased). (B-E) Analysis on patient population including 3 patients with NK cell that stayed stable or increased: (B) On-treatment to baseline ratio of positive cell fraction levels in D-Rd and Rd in function of the signal intensity centiles for a subset of markers across CD56^{dim} and CD56^{bright} NK cells. (C) Radviz projection with contour plots representing marker expression levels of CD56^{bright} and CD56^{dim} cells at baseline and upon D-Rd and Rd treatment. (D) Percentages of CD137^{+} CD56^{dim}, and CD56^{bright} NK cells relative to total CD56^{dim} and CD56^{bright} NK cells, respectively. (E) Percentages of subtypes of CD27 and CD69 NK cells relative to total NK cells. C, Cycle; D, Day; D-Rd, daratumumab, lenalidomide, dexamethasone; NK, natural killer; Rd, lenalidomide, dexamethasone.

A.
CD56^{dim}

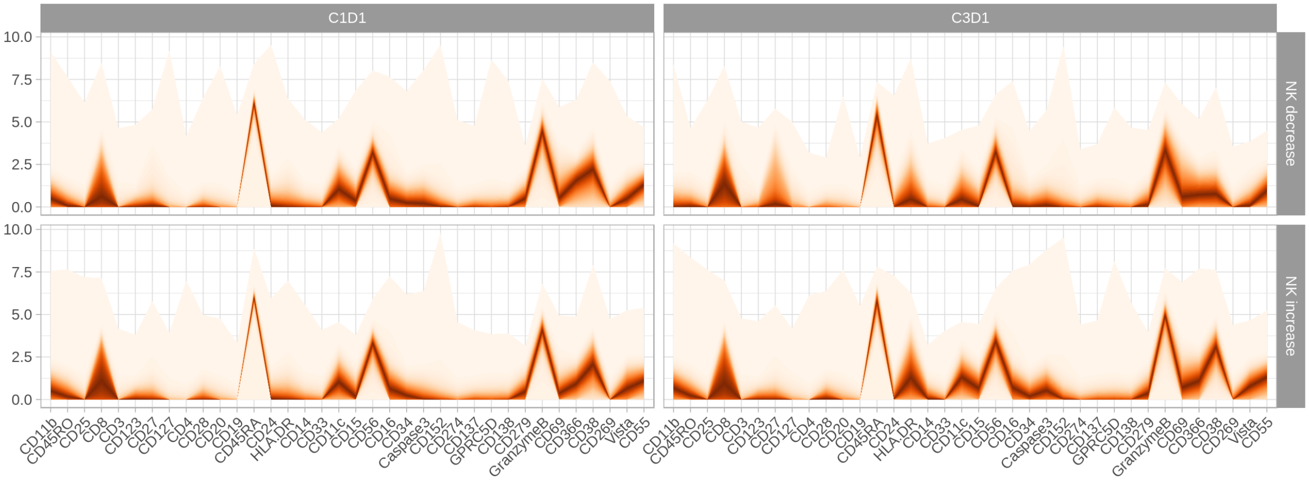

B.
CD56^{bright}

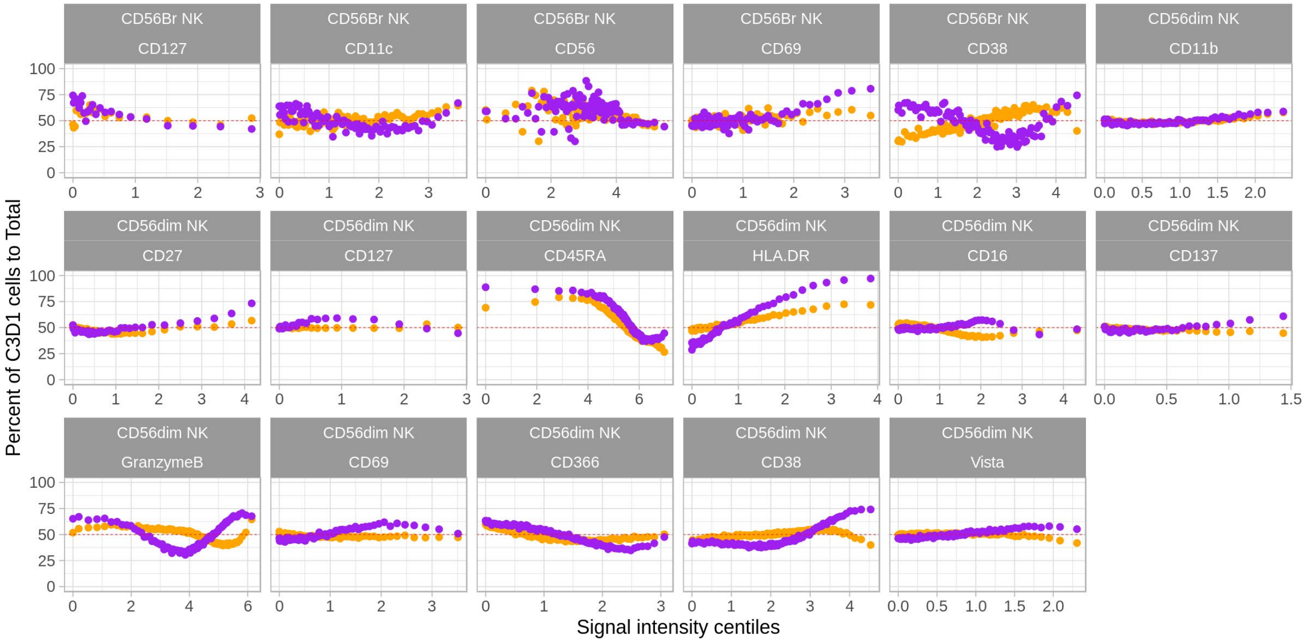

C.

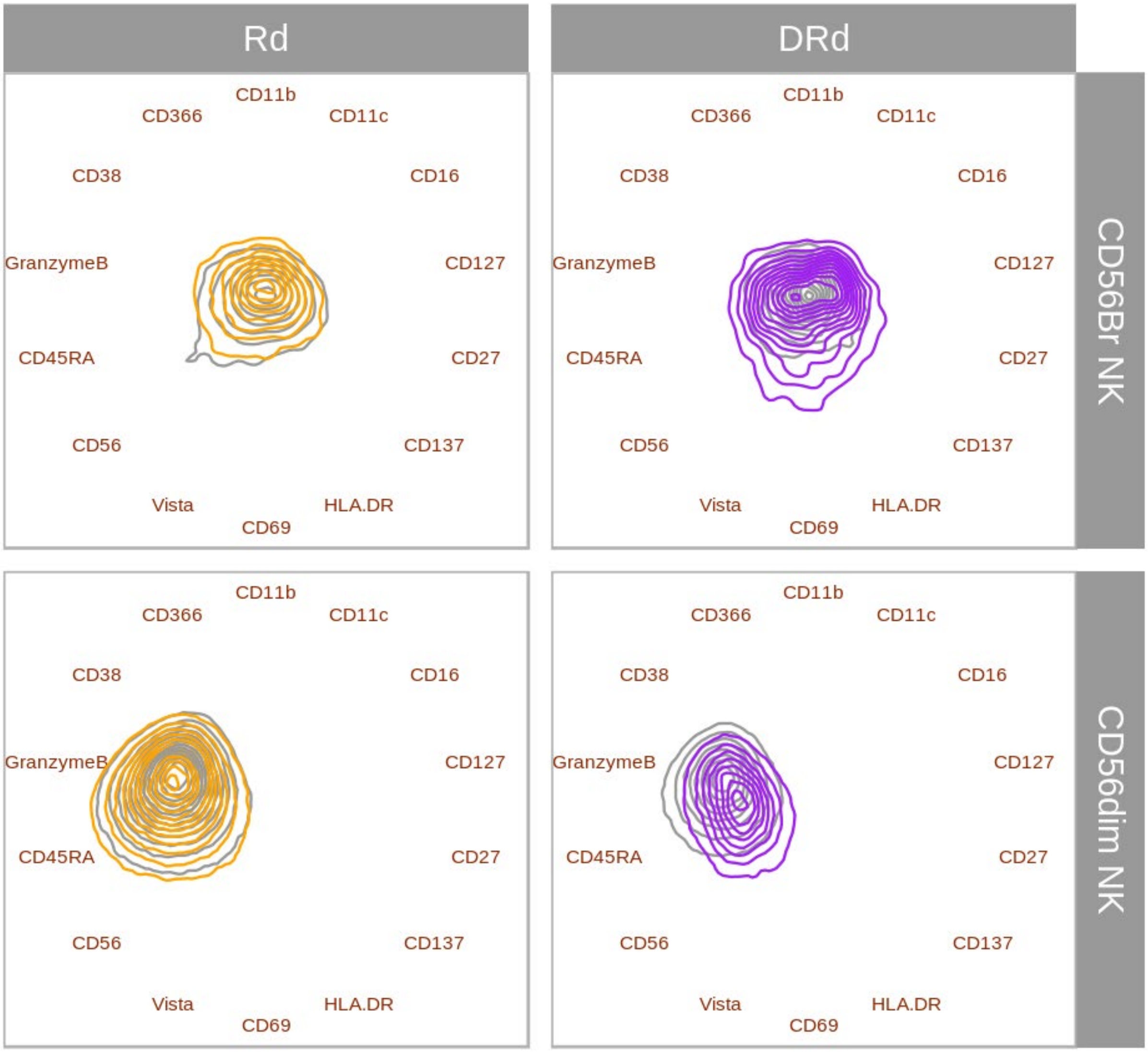

Rd
DRd
CD56Br NK
CD56dim NK
CD11b
CD366
CD11c
CD38
CD16
GranzymeB
CD127
CD45RA
CD27
CD56
CD137
Vista
HLA.DR
CD69

D.

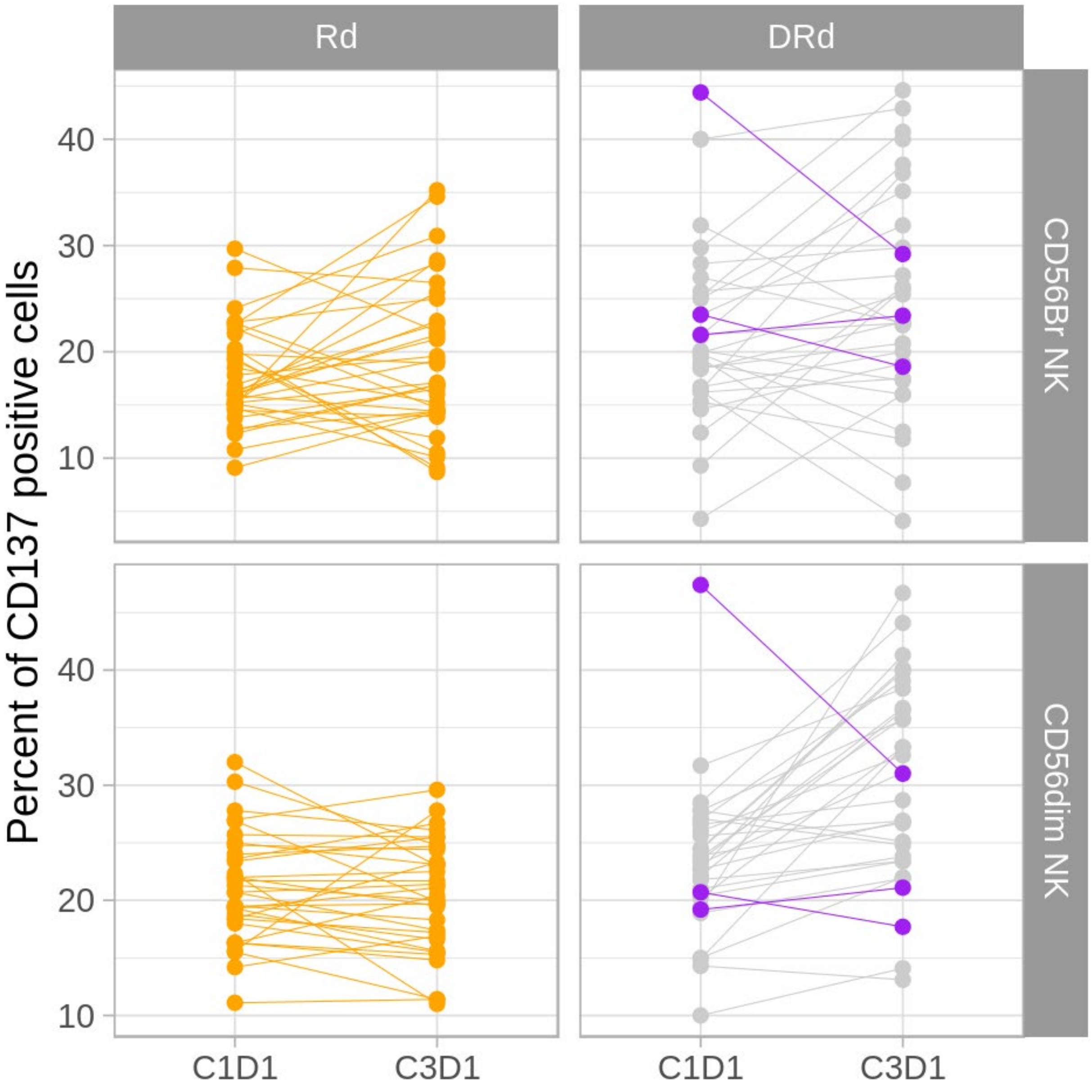

Rd
DRd
CD56Br NK
CD56dim NK
Percent of CD137 positive cells
40
30
20
10
C1D1
C3D1

E.

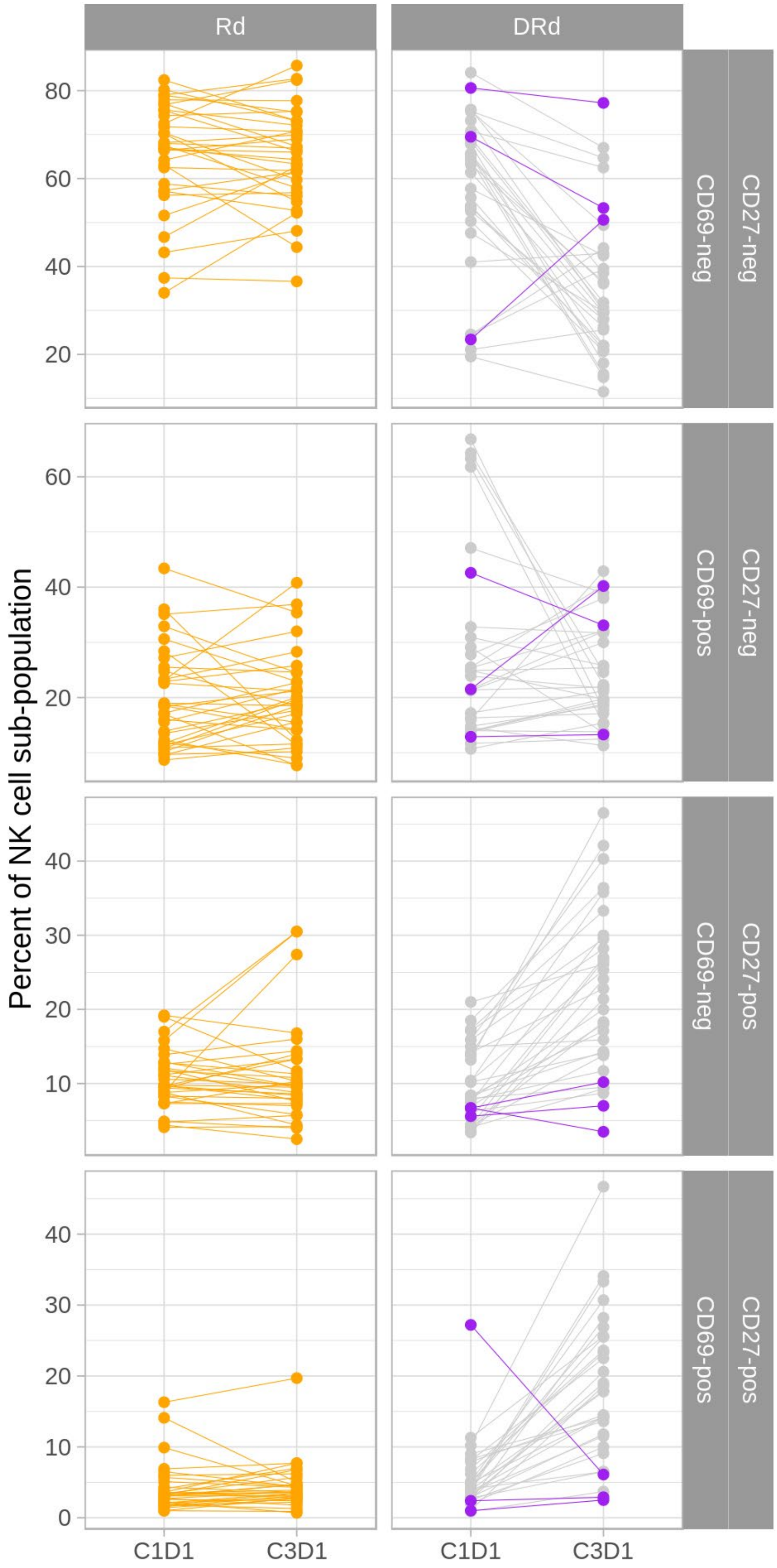

Rd
DRd
CD69-neg
CD27-neg
CD69-pos
CD27-neg
CD69-neg
CD27-pos
CD69-pos
CD27-pos
Percent of NK cell sub-population
80
60
40
20
60
40
20
40
30
20
10
40
30
20
10
0
C1D1
C3D1
C1D1
C3D1

Figure S5. On-treatment to baseline ratio of CD38 expression levels on PBMCs, lymphocytes, T_{reg}, and CD8 T-cell fractions in D-Rd and Rd treatment groups.
(A) PBMCs, (B) lymphocytes, (C) T_{reg}, (D) CD8 T-cell, and (E) basophil fractions in D-Rd and Rd treatment groups. C, Cycle; D, Day; D-Rd, daratumumab, lenalidomide, dexamethasone; MFI, median fluorescence intensity; NK, natural killer; PBMC, peripheral-blood mononuclear cell; Rd, lenalidomide, dexamethasone; T_{reg}, regulatory T cell.

A.

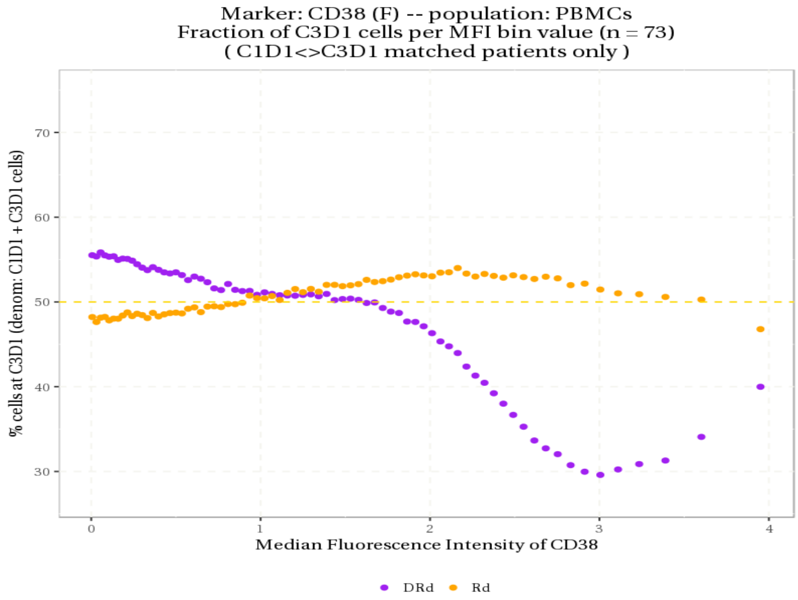

B.

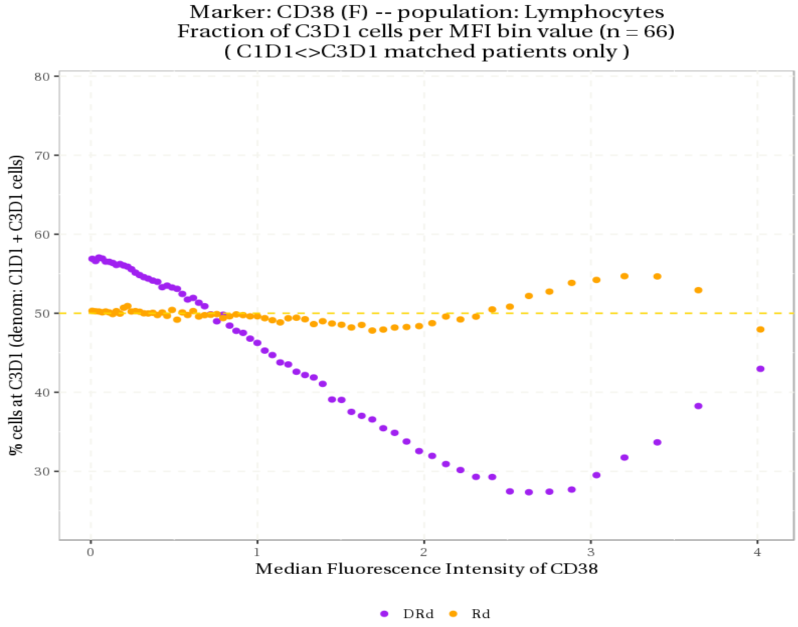

C.

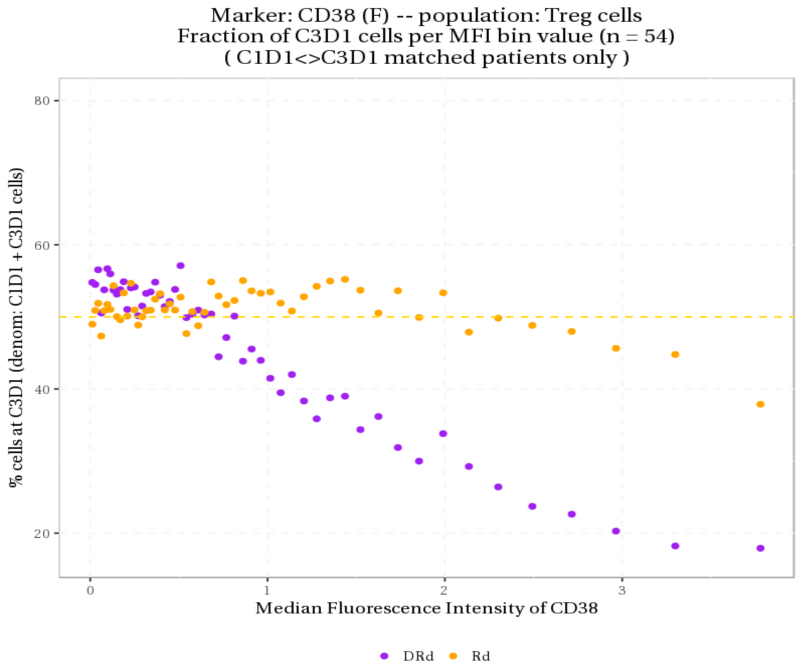
Marker: CD38 (F) -- population: Treg cells
Fraction of C3D1 cells per MFI bin value (n = 54)
(C1D1<>C3D1 matched patients only)
% cells at C3D1 (denom: C1D1 + C3D1 cells)
80
60
40
20
0
1
2
3
Median Fluorescence Intensity of CD38
DRd
Rd

D.

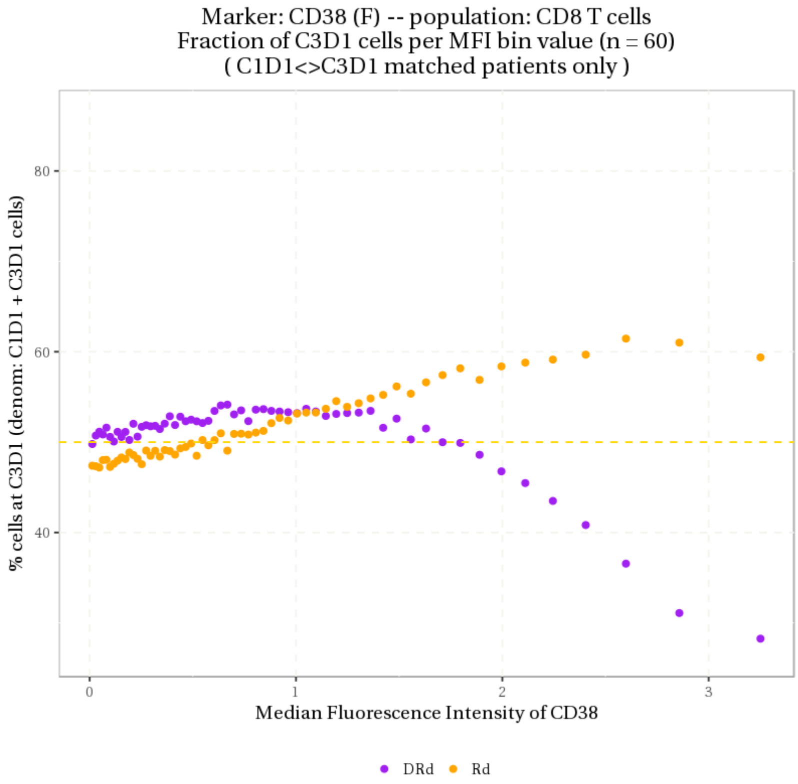
Marker: CD38 (F) -- population: CD8 T cells
Fraction of C3D1 cells per MFI bin value (n = 60)
(C1D1<>C3D1 matched patients only)
% cells at C3D1 (denom: C1D1 + C3D1 cells)
80
60
40
0
1
2
3
Median Fluorescence Intensity of CD38
DRd
Rd

E.

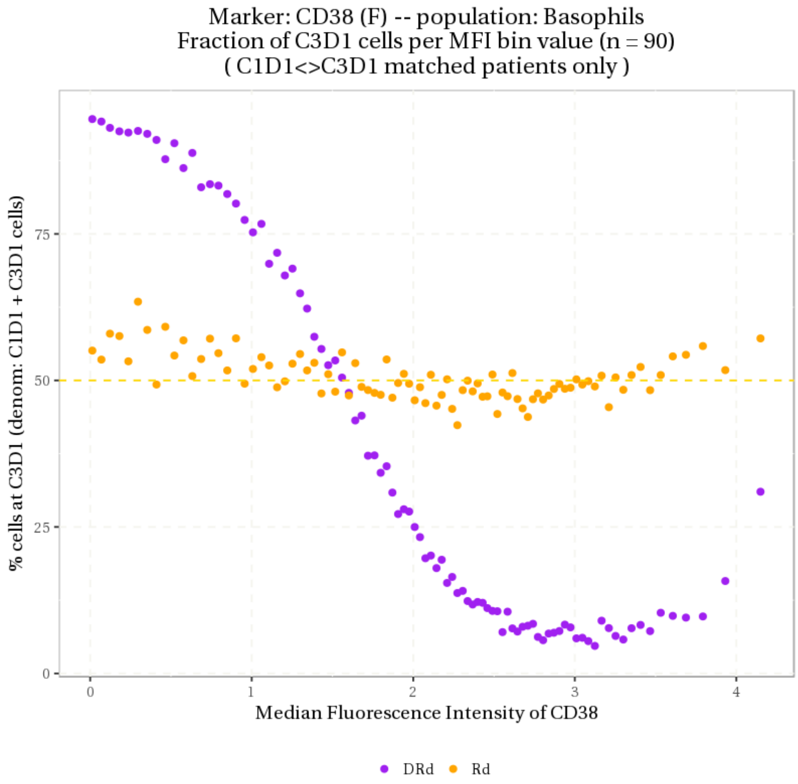

Marker: CD38 (F) -- population: Basophils
Fraction of C3D1 cells per MFI bin value (n = 90)
(C1D1<>C3D1 matched patients only)
% cells at C3D1 (denom: C1D1 + C3D1 cells)
75
50
25
0
0
1
2
3
4
Median Fluorescence Intensity of CD38
DRd
Rd

Figure S6. T-cell clonality, T-cell clonality per patient, and T-cell richness with D-Rd compared with Rd.

(A) T-cell clonality (B) T-cell clonality per patient, and (C) T-cell richness. D-Rd, daratumumab, lenalidomide, dexamethasone; Rd, lenalidomide, dexamethasone.

A.

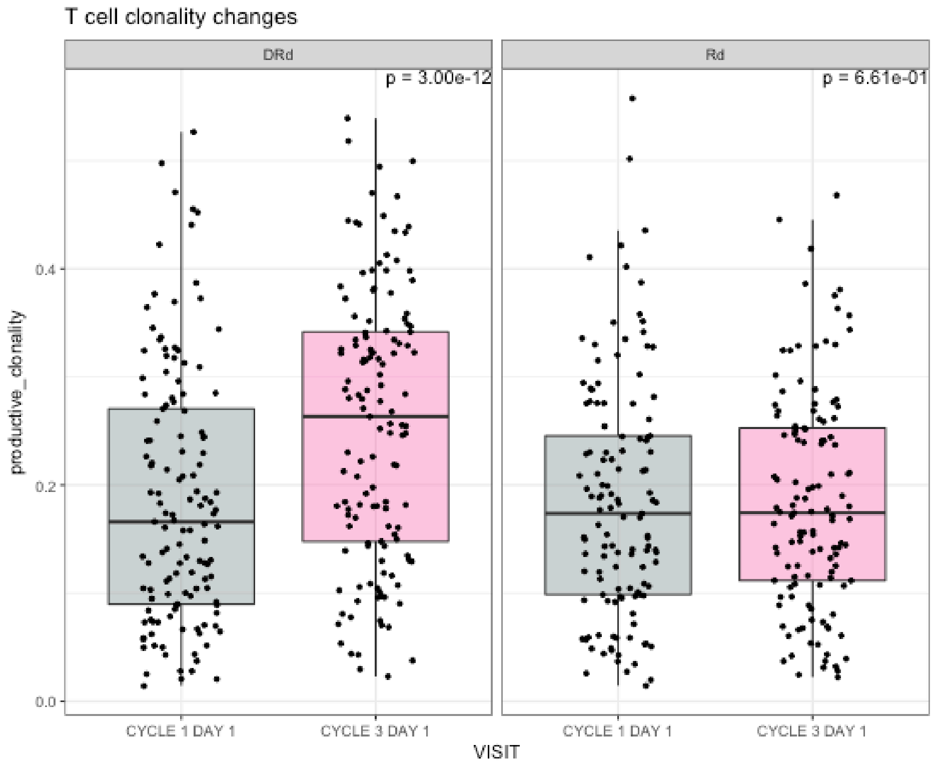

B.

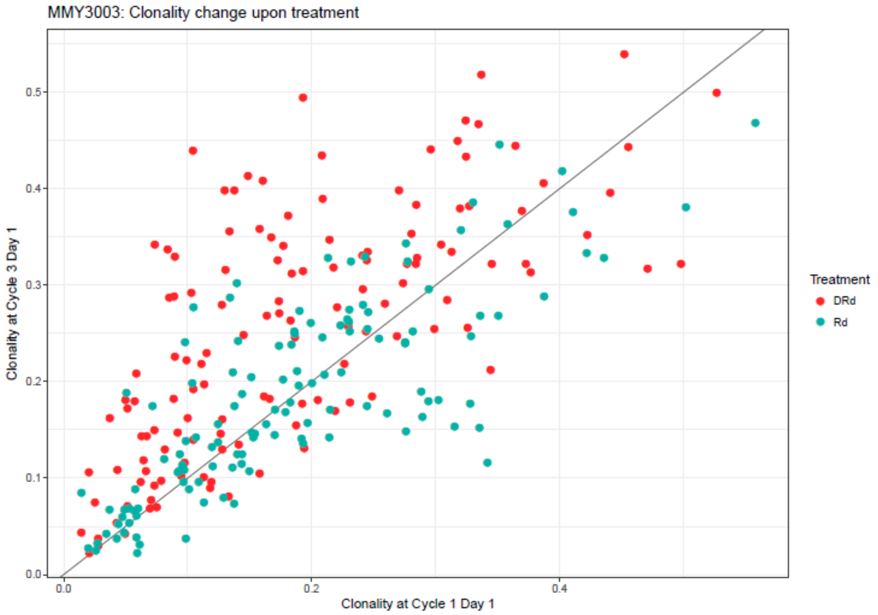

C.

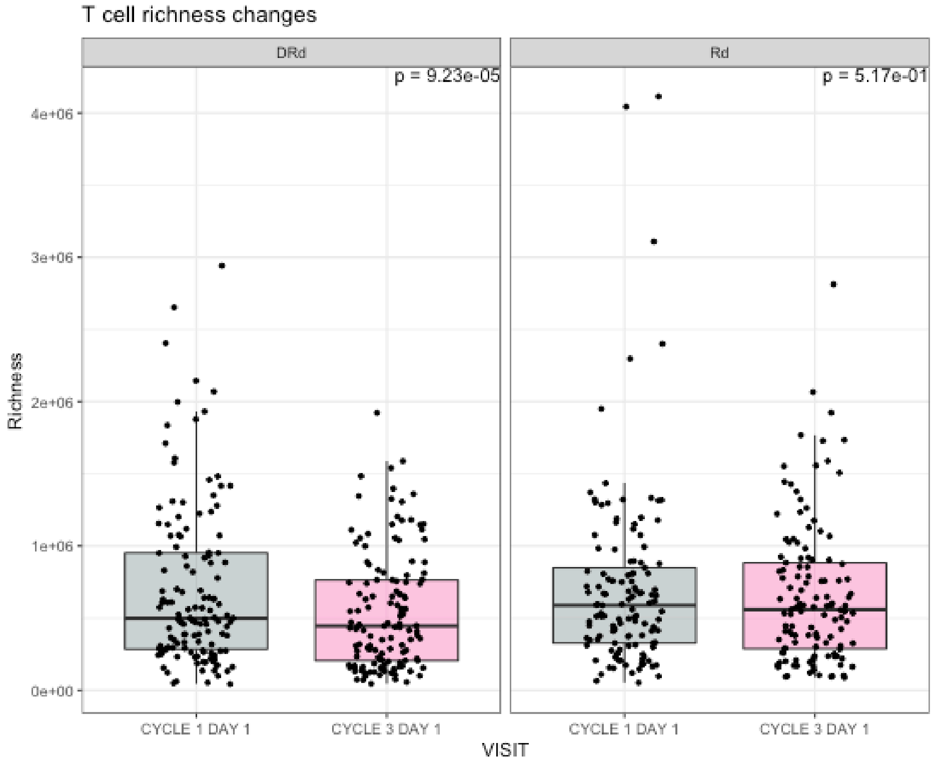
T cell richness changes
DRd
Rd
p = 9.23e-05
p = 5.17e-01
4e+06
3e+06
2e+06
1e+06
0e+00
Richness
CYCLE 1 DAY 1
CYCLE 3 DAY 1
CYCLE 1 DAY 1
CYCLE 3 DAY 1
VISIT
